# Most European countries will miss EU targets on antibacterial use by 2030: historical analysis of European and OECD countries, comparison of community and hospital sectors and forecast to 2040

**DOI:** 10.1007/s00210-025-03887-5

**Published:** 2025-02-17

**Authors:** Lilly Josephine Bindel, Roland Seifert

**Affiliations:** Institute of Pharmacology, Hannover Medical School, D-30625 Hannover, Germany

**Keywords:** Antimicrobial consumption, Antibiotic, Antibacterial drug, AMR, AMC, Antibiotic prescription, Europe, Surveillance, Antibiotic Stewardship, Rational prescribing behaviour, Irrational prescribing behaviour, Forecast, ARIMA, One Health, ECDC, EU, OECD

## Abstract

The rise of bacterial resistance threatens the treatment of infections and is closely linked to the consumption of antibacterial drugs. The EU’s ‘One Health’ approach aims to address this issue by requiring Member States to reduce consumption by 20% by 2030. This study analyses antibacterial consumption trends in the total care of European and non-European OECD countries, compares the community and hospital sector, uses ARIMA modelling and correlation analysis to provide long-term forecasts, assesses patterns of consumption and evaluates whether current trends are in line with the EU target. Projections to 2040 show increases for Spain (+36.6%; 30.7 DID), Greece (+31.4%; 37.5 DID), Czechia (+29.7%; 19.4 DID), Bulgaria (+28.3%; 33. 7 DID), Malta (+26.5%; 28.8 DID), Denmark (+25.7%; 19.8 DID), Croatia (+17.4%; 24.9 DID), Italy (+13.7%; 26.3 DID), Germany (+7.6%; 12.6 DID), Australia (+12.4%, 18.2 DID), Canada (+8.0%, 14.8 DID), Chile (+90.1%, 66.7 DID), Costa Rica (+0.4%, 19.7 DID), Japan (+22.7%, 12.8 DID) and Korea (+24.3%, 31.9 DID). Declines are forecast for Belgium (−0.5%; 20.5 DID), Romania (−0.6%; 27.2 DID), Cyprus (−1.0%; 33.2 DID), Luxembourg (−2.2%; 19.8 DID), Norway (−3.4%; 15.1 DID), Latvia (−5.5%; 14.1 DID), Lithuania (−6.4%; 17.5 DID), the Netherlands (−8.4%; 8. 8 DID), Portugal (−10.6%; 16.1 DID), Estonia (−12.1%; 11.2 DID), Slovakia (−16.1%; 16.8 DID), France (−17.7%; 19.8 DID), Hungary (−20.4%; 11.3 DID), Slovenia (−21.9%; 10.5 DID), Finland (−24.8%; 9.7 DID), Iceland (−24.9%; 13.9 DID), Sweden (−30.4%; 7.0 DID) and Israel (−70.7%, 4.7 DID). A significant positive correlation was found between current versus projected consumption levels and changes, highlighting stable prescribing patterns in many countries. Northern and Central Europe maintain low levels of consumption with decreasing trends, whereas Latin America, Eastern and Southern Europe show higher levels with projected increases. Western Europe and Asia shows a mixed pattern, with varying trends of increase and decrease. Alarmingly, only Sweden is projected to meet the 20% reduction target by 2030. Even in the best-case scenario, only a proportion of European countries are projected to meet the target, including Austria, Belgium, Croatia, Cyprus, Finland, France, Greece, Hungary, Italy, Iceland, Lithuania, Luxembourg, Latvia, Portugal, Slovenia, Slovakia and Spain, while 11 countries show no potential for a successful reduction. The reliability of the projections is considered good to moderate. Divergent trends between the community and hospital sectors further complicate the assessment of progress and underline the need for targeted interventions. Current trends suggest that the EU targets are unlikely to be met, highlighting the urgent need to strengthen stewardship programmes. Further research is needed to address other objectives of the ‘One Health’ approach, including the use of classes of antibacterial drugs and the development of bacterial resistance.

**Supplementary Information:**

The online version contains supplementary material available at 10.1007/s00210-025-03887-5.

## Introduction

Antibacterial resistance (AMR) is one of the major global health challenges (EMA 2024; WHO 2022). Despite intensified efforts to combat its spread, progress has been inconsistent, with some pathogens showing reduced resistance while others continue to increase at an alarming rate (ECDC 2024a, 2024b). A well-established driver of AMR is the high and often inappropriate consumption of antibacterial drugs (ECDC 2024a; Bindel and Seifert 2024a, 2024b, 2024c). As a result, reducing the consumption of antibacterial drugs has been a central focus of public health strategies in Europe to tackle AMR.

European action plans, such as the ‘One Health’ approach, have introduced comprehensive measures, aiming to reduce the consumption of antibacterial drugs, promoting rational prescribing practices and increasing the use of beneficial agents as classified in the 'Access' group by the WHO’s AWaRe framework. These plans also aim to reduce bloodstream infections caused by resistant bacterial strains, particular for methicillin-resistant *S. aureus* (MRSA) by −15%, cephalosporin-resistant *E. coli* by −10% and carbapenem-resistant *K. pneumoniae* by −5% (European Union 2023). Despite these efforts, the decline in antibacterial use has been slower than anticipated (ECDC 2024a), raising concerns about the likelihood of meeting the EU’s 2030 reduction targets. A more detailed, long-term analysis of antibacterial consumption patterns and a forecast of likely outcomes is therefore urgently needed.

This study addresses this deficit by systematically analysing antibacterial consumption trends in different health care sectors and projecting future developments using ARIMA modelling. Focusing primarily on European countries, but including data from non-European OECD countries for a global context, it assesses whether current prescribing patterns are consistent with EU reduction targets. A key objective is to assess the feasibility of achieving these targets and to explore the wider public health implications of consumption trends.

Previous research by our group has identified key factors influencing antibacterial consumption (Bindel and Seifert 2024a, 2024b, 2024c, 2024d) and revealed significant correlations between antibacterial consumption and AMR trends (Bindel and Seifert 2024b). Moreover, prior studies have highlighted persistent irrational prescribing practices (Bindel and Seifert 2024b, 2024c, 2024d) and successfully predicted future consumption of antibacterials and other drug classes using ARIMA methodology (Bindel and Seifert 2025a, 2025b). These results provide a basis for understanding the drivers of consumption and the potential impact of policy interventions.

While existing studies focus on retrospective analysis of past trends (ECDC 2024a), this study provides a forward-looking perspective by forecasting the consequences of current prescribing behaviours. By providing long-term projections, it aims to support efforts to strengthen rational prescribing practices and implementing targeted interventions to combat AMR. Ultimately, these insights contribute to the development of evidence-based approaches to antimicrobial stewardship and reinforce efforts to meet the EU’s 2030 targets.

## Methods and materials

### Setting and data

The analysis focuses on national consumption data for ATC J01 (antibacterials for systemic use). Data for European countries are based on the ECDC Antimicrobial Consumption Dashboard (https://qap.ecdc.europa.eu/public/extensions/AMC2_Dashboard/AMC2_Dashboard.html#eu-consumption-tab) (ESAC-net 2024a, 2024b), spanning from 1997–2023. Data for non-European countries are based on the OECD data explorer (https://data-explorer.oecd.org/) (OECD 2025a) and covers the period from 1990 to 2023. Data are expressed in DDD prescriptions per 1000 inhabitants per day (DID). Data availability varies in terms of available years and published care sectors (hospital, community, total care), as depicted in Tables 1 and 2.

**Table 1:** Overview of data availability for the European countries analysed. In addition to the years available for each sector, the sector chosen for the forecasting model is mentioned. In some cases, the community sector was modelled because no or insufficient data were available for the total care sector

Country	Community sector available years	Hospital sector available years	Total care sector available years	Chosen sector for forecast model (preferably total care sector)
Austria	1998–2023	2019–2023	2019–2023	community sector
Belgium	1997–2005; 2007–2023	1997–2023	1997–2023	total care sector
Bulgaria	2007–2023	2006–2023	1999–2023	total care sector
Croatia	2000–2023	2000–2023	2000–2023	total care sector
Cyprus	no data	no data	2006–2022	total care sector
Czechia	1998–1999; 2003–2015; 2021–2023	2021–2023	2019–2023	community sector
Denmark	1997–2023	1997–2023	1997–2023	total care sector
Estonia	2002–2023	2002–2023	2001–2023	total care sector
Finland	1997–2023	1997–2023	1997–2023	total care sector
France	1997–2023	1997–2023	1997–2023	total care sector
Germany	1997–2023	2023	2002–2004; 2023	community sector
Greece	1997–2004; 2010; 2012–2023	1997–2003; 2009; 2011–2023	1997–2023	total care sector
Hungary	1998–2024	2001–2023	2001–2023	total care sector
Ireland	1998–2023	2004–2023	2004–2023	total care sector
Italy	1999–2023	2007–2008; 2010–2023	2005; 2007–2008; 2010–2023	total care sector
Iceland	2006–2009; 2014–2023	2017–2023	1997–2005; 2010–2013; 2017–2023	total care sector
Lithuania	2012–2023	2012–2023	2006–2023	total care sector
Luxembourg	1997–2023	1997–2023	1997–2023	total care sector
Latvia	2002; 2004–2023	2002; 2004–2023	2002; 2004–2023	total care sector
Malta	2007–2023	1997–2023	2007–2023	total care sector
Netherlands	1997–2023	1997–2002; 2010–2023	1997–2002; 2010–2023	total care sector
Norway	1998; 2001–2023	1998; 2001–2023	1998; 2001–2023	total care sector
Poland	1998–2005; 2007–2023	1998–2002; 2014–2023	1998–2002; 2004; 2014–2023	community sector
Portugal	1997–2006; 2007–2023	2010–2023	2009–2023	total care sector
Romania	2009; 2019–2023	2009–2010; 2019–2023	2009; 2011–2023	community sector
Slovenia	1997–2023	1998–2023	1997–2023	total care sector
Slovakia	1999–2009; 2012–2023	1999–2002; 2004–2009; 2012–2023	1999–2009; 2010–2023	total care sector
Spain	1997–2023	2016–2023	2016–2023	community sector
Sweden	1997–2021	1997–2021	1997–2021	total care sector
UK	1997–2019	2013–2019	2013–2019	total care sector

**Table 2:** Overview of data availability for the non-European OECD countries analysed. Mentioned are the years with available data as well as the reported time breaks and differing definitions (OECD 2024b)

Country	Available years	Marked as definition differs	Main reasons for differing definition	Reported time series break	Reason for distortion	Definition of outliers in ARIMA(1,1,1)
Australia	1990–2022	yes	exclusion of hospital sector and OTC	yes, 2013	exclusion of private prescriptions	2013 level shift; 2020 transient
Canada	2007–2023	yes	only three provinces included (British Columbia, Manitoba, Saskatchewan); only certain drug programs included	yes, 2016	drug information system, therefore inclusion of all claims (public, private, OTC)	2016 level shift, 2020 transient
Chile	2011–2023	no	-	no	-	2020 transient
Costa Rica	2001–2023	no	-	yes, 2007	change in reporting institution	2007 level shift, 2020 transient
Israel	2012; 2013–2023	yes	not all regions included; only outpatient sector included	no	-	2020 transient
Japan	2011–2020	no	-	no	-	2020 transient
Korea	2008–2022	no	-	yes, 2011, 2016	change in methology	2011 level shift, 2016 level shift, 2020 transient

For the following 37 countries, consumption data was available: Austria, Belgium, Bulgaria, Croatia, Cyprus, Czechia, Denmark, Estonia, Finland, France, Germany, Greece, Hungary, Ireland, Italy, Iceland, Lithuania, Luxembourg, Latvia, Malta, the Netherlands, Norway, Poland, Portugal, Romania, Slovenia, Slovakia, Spain, Sweden, the UK, Australia, Canada, Chile, Costa Rica, Israel, Japan and Korea.

Not all sectors were available for each country, due to the methodologies used. To build a robust forecasting model, it is essential to have as many data points as possible. For European countries, it was decided to build a model based on the total care sector in order to include all developments and trends. However, not all countries published sufficient data for the total care sector, as some countries only considered the outpatient sector. For these countries, the community sector was used as a substitute. These countries are Austria, Czechia, Germany, Poland, Romania and Spain.

### Selection of method for time series analysis

The ARIMA model, which stands for “Auto Regressive Integrated Moving Average”, was selected for its versatility in handling various components of a time series, including autoregressive (AR) behaviour, differencing (I), and moving average (MA) effects (Bindel and Seifert 2025a). This model is particularly well-suited to data such as ours, where past observations influence future values. ARIMA models are widely used in forecasting medicine consumption trends (Hyndman and Athanasopoulos 2021).

It comprises three key components. The autoregressive (AR) part accounts for the influence of previous observations on the current value. The differencing (I) component helps in making the series stationary by removing trends or seasonality. Finally, the moving average (MA) component captures the dependency between an observation and past forecast errors (Nau 2020; Box et al. 2015).

The general form of an ARIMA(*p,d,q*) model is expressed as follows:

$$\widehat{y}t = \mu + \phi 1 yt-1 +\dots + \phi p yt-p - \theta 1et-1 -\dots - \theta qet-q$$

In this equation, $\widehat{y}t$ represents the value of the series at time, $t, p$ refers to the number of autoregressive terms, *d* is the degree of differencing applied to make the series stationary, and *q* is the number of lagged forecast errors in the moving average model. The error term $et$ captures the random shocks that cannot be explained by the model (Nau 2020; Box et al. 2015).

### Data preprocessing for time series analysis

A precondition for ARIMA modelling is data stationarity (Hyndman and Athanasopoulos 2021). Autocorrelation function (ACF) and partial autocorrelation function (PACF) tests were applied on each medicine in the dataset, revealing significant trends that confirmed the presence of non-stationarity in the original time series, which is also visible within the plotted development (Fig. S1–S29). Consequently, differencing was necessary to remove these trends, ensuring the necessary stationarity for accurate predictions (Fig. S30–S59). Differencing allows the ARIMA model to capture temporal dependencies without the distortion caused by persistent trends, enabling more reliable forecasting.

### Determination of parameters

The ARIMA model is shaped by its three parameters *p, d* and *q.* The autoregressive term *p* defines how many past observations influence the current prediction, with higher values increasing reliance on historical data and potentially adding complexity. The differencing order *d* helps achieve stationarity by removing trends in the data, but excessive differencing may lead to over-fitting and loss of important structure. Finally, the moving average term *q* determines how many past forecast errors affect current predictions, where larger values mean more past errors are incorporated, potentially raising the model’s complexity (Hyndman and Athanasopoulos 2021; NIST/SEMATECH 2012). The OECD data aims to cover the complete consumption (OECD 2025b), but did not provide data for different sectors. Deviating methodologies from were marked as ‘definition differs’ (Table 2).

Optimal values for *p*, *d*, and *q* were identified post-preprocessing, based on an analysis of the ACF and PACF plots, which highlighted significant lag structures in the data. The differencing parameter *d*=1 was selected to remove the trend and achieve stationarity in the time series. The autoregressive term *p* and the moving average term *q* were determined by analysing the autocorrelation function (ACF) and partial autocorrelation function (PACF) plots.

An important variation of the ARIMA model is ARIMA(1,1,1), which was used in our analysis. This model offers a moderate forecast with narrow ranges, balancing adjustments and low complexity. It employs first-order differencing to eliminate trends while including autoregressive and moving average components. As a comprehensive ARIMA model, it is able to handle complex data characterized by both trend and noise, but comes with a risk of overfitting (Nau 2020; Box et al. 2015). This model has proven effective in forecasting medicinal trends in several studies (Doroftei et al. 2022; Wang et al. 2019).

### Model adjustments

The ARIMA models were developed using SPSS, further analysis was conducted in Excel. For European countries, structural changes in definitions and methodological procedures were not explicitly mentioned by the ECDC (ESAC-net 2024b). A manual transient outlier was defined for the year 2020 due to the effects of the COVID pandemic, which severely distorted the projections if not implemented. The OECD provided a method section, where time breaks and differing definitions for each country were mentioned (OECD 2025b), enabling to define outlier for time breaks (Table 2).

ARIMA(1,1,1) models were successfully developed for 36 of 37 countries, with the exception of the UK, where the limited number of data points prevented model creation. While more complex models might better capture underlying regularities and offer more precise forecasts—indicated by higher stationary *R*-squared and *R*-squared values—there is a significant risk of overfitting, where random fluctuations are mistakenly identified as patterns. Given the relatively limited dataset, the model's capacity is restricted. Therefore, greater emphasis should be placed on the reliability of trends rather than the precise prediction of specific DDD values in long-term periods, while forecast for a shorter time period are considered to be more reliable (Bindel and Seifert 2025a).

### Forecast periods

Forecasts were extended to the year 2040, providing long-term projections of consumption trends for the total care sector for each country (Table S1–S6). In addition, confidence intervals were calculated to illustrate the uncertainty of these projections, indicating a range within which future consumption values are likely to fall.

For the forecast, the last provided consumption data, mostly around 2023, was compared with the projection for 2040 to provide a long-term assessment. In order to assess the EU’s ‘One Health’ objective, a further analysis considered the changes from 2019 to 2030.

### Assessment of fit metrics

To assess the performance of the ARIMA models, several fit metrics were used. This includes stationary *R*-squared, *R*-squared, RMSE (Root Mean Squared Error), MAPE (Mean Absolute Percentage Error), MAE (Mean Absolute Error) and normalised BIC (Bayesian Information Criterion) (Hyndman and Athanasopoulos 2021; NIST/SEMATECH 2012; Bindel and Seifert 2025a).

The Stationary *R*-squared and *R*-squared metrics assess how well the model explains the variance in the data. Stationary R-squared is particularly useful for differenced data, as it indicates how well the model fits on a stationary scale. Values of 0.65 and above are considered a good fit, while values between 0.4 and 0.64 are moderate, and anything below 0.4 is poor. On the other hand, *R*-squared measures the model’s overall explanatory power, with values above 0.85 considered good, between 0.6 and 0.84 as moderate and below 0.6 as poor.

RMSE and MAE are absolute error measures that assess prediction accuracy. RMSE, which emphasizes larger deviations, is more sensitive to outliers, while MAE provides the average of absolute deviations. Ideally, both metrics should approach zero. However, their interpretation depends on the scale of the data, making context decisive for evaluating whether an RMSE or MAE value is good, moderate or poor.

MAPE and MaxAPE quantify forecast accuracy as percentages, making them useful for comparing models across different scales. MAPE values under 6% indicate a good fit, between 7% and 20% are moderate, while values exceeding 20% are poor. MaxAPE focuses on the worst-case relative error, with values below 15% regarded as good, 16% to 40% as moderate and anything higher as poor.

MaxAE measures the largest single deviation observed in the model’s predictions, highlighting its worst-case performance. Lower values are preferred, indicating that the model avoids significant prediction errors.

Finally, the Normalized BIC assesses model fit in relation to complexity. Lower BIC values indicate a better balance between accuracy and simplicity, as the BIC penalizes models with unnecessary parameters to prevent overfitting.

Together, these metrics provide a comprehensive evaluation of both the accuracy and efficiency of the ARIMA models, enabling a balanced assessment of model performance across different countries and time series data.

### Correlation analysis

Bivariate correlation analysis was performed in SPSS. There was a focus on three particular aspects of the correlations: significance, direction (positive vs. negative), and strength. The Pearson coefficient indicates whether there is a linear relationship between the two variables. The correlation coefficient ranges from −1 to +1. A positive coefficient indicates that both variables influence each other in the same direction, while a negative coefficient indicates an inverse relationship. A value of 0 signifies no linear relationship, while a value of 1 indicates a very strong linear relationship with same proportions of growth (Mukaka 2012). We define a correlation above (+/−) 0.8 as strong, indicating a substantial influence between both factors. Values below 0.8 suggest a weaker relationship.

Beside the correlation coefficient, the significance of the correlation should be considered. The significance level indicates the extent to which the values can be generalized and considered reliable. Only significant values validate the correlation coefficient, allowing to draw conclusions. If there is non-significance, the values are only limited informative. A value with a significance level of 0.01 as well as 0.05 is determined to be considered significant.

The coefficient of determination (*R*^2^), calculated by squaring the Pearson correlation coefficient, indicates the proportion of the variance in the dependent variable that is predictable from the independent variable. Beside the significance, it can be used as an indicator whether the given correlation is valid.

### Comparison of care sectors

In order to gain a deeper insight into the trends of the specific sectors, the community and hospital sectors were compared for European countries where data were available for both sectors. To allow comparison between countries, the range of relative change was set for the years around 2000–2023, as this is when most countries have data available and it covers a long period. For some countries, data collection started later and they are excluded from this comparison. To provide a long-term assessment for each country, the relative change from the first to the last data point was compared.

For 28 countries, at least one data point was available for both sectors, including Austria, Belgium, Bulgaria, Croatia, Czechia, Denmark, Estonia, Finland, France, Germany, Greece, Hungary, Ireland, Italy, Iceland, Lithuania, Luxembourg, Latvia, Malta, the Netherlands, Norway, Poland, Portugal, Slovenia, Slovakia, Spain, Sweden and the UK.

The direction of change was assessed, whether increasing or decreasing. If both sectors have the same direction, the country is considered to have similar sectoral trends, while if one sector increases and the other decreases, the country is considered to have opposite trends.

**Fig. 1:**
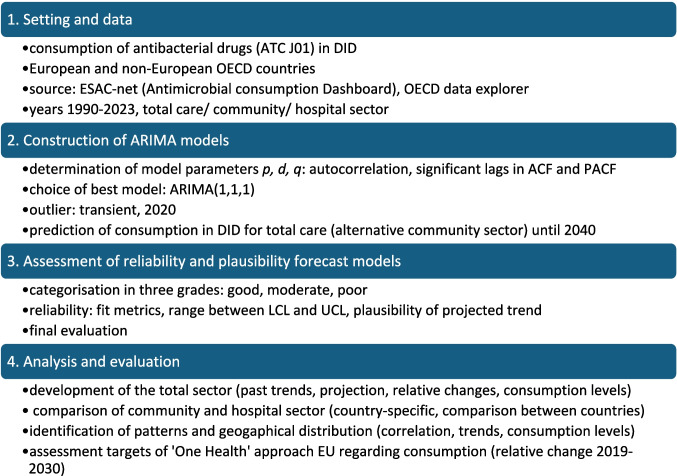
Methodical approach for time series analysis in SPSS with the ARIMA-model to predict futural development of DDD-prescriptions for antibacterial drugs

The methodological procedure is illustrated in Fig. 1.

## Results

### Development of antibacterial drug consumption in the total care sector in European countries

**Fig. 2:**
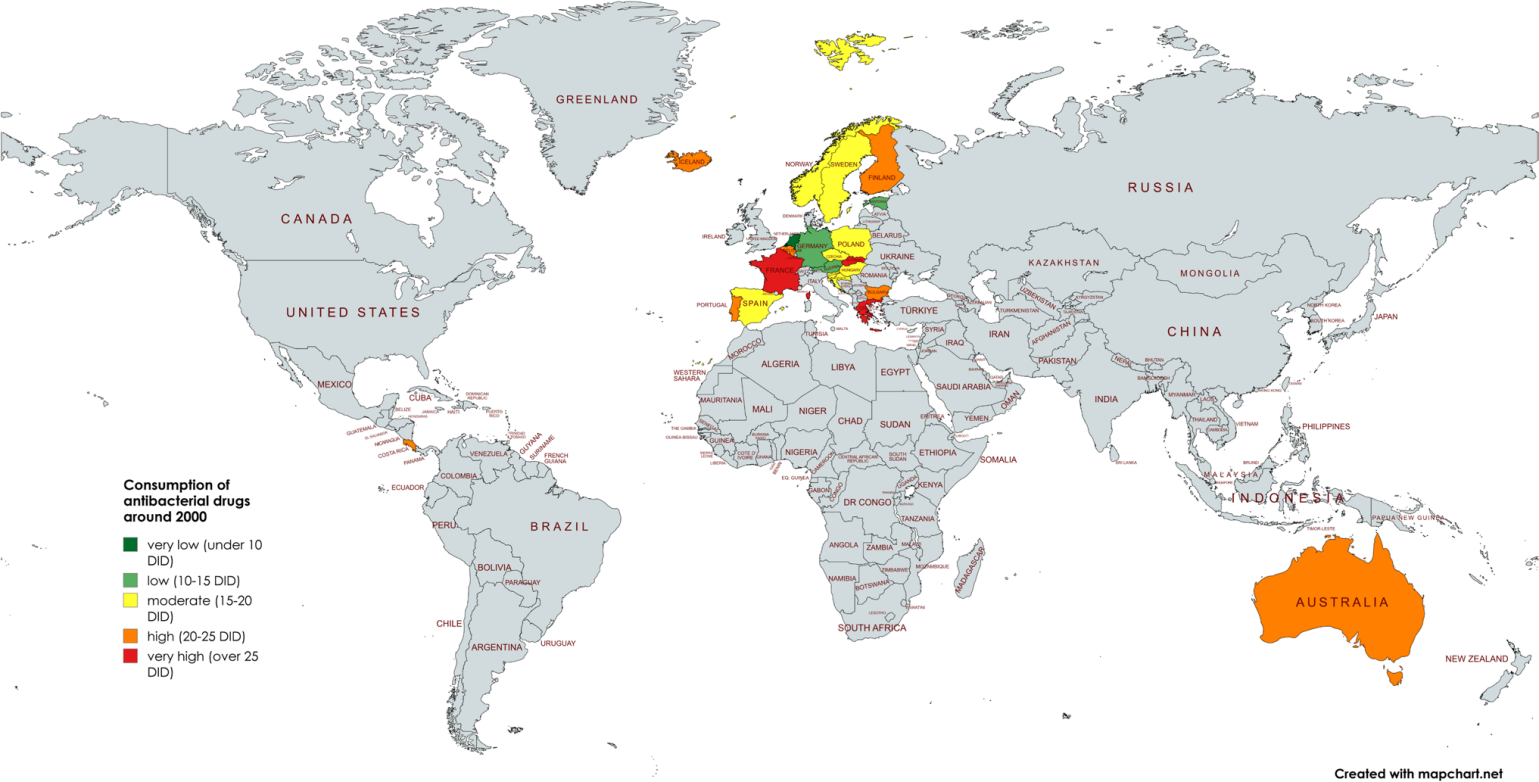
Development of the consumption of antibacterial drugs around 2000. The total care sector is shown where available. A consumption level below 10 DID is coloured dark green, a level between 10 and 15 DID is coloured light green, a moderate level between 15 and 20 DID is coloured yellow, a high level between 20 and 25 DID is coloured orange and a very high level exceeding 25 DID is coloured red. Countries where no data was available are grey-coloured. The map was created using mapchart.net

**Fig. 3:**
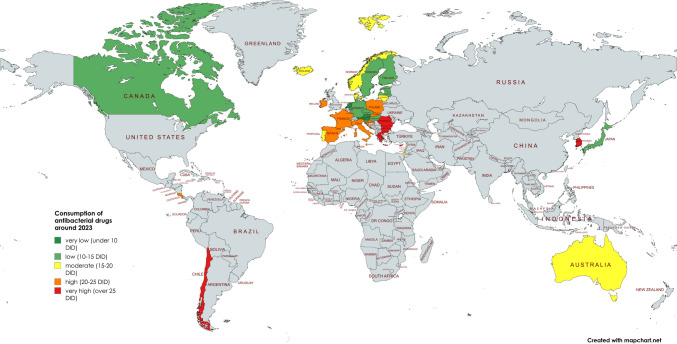
Development of the consumption of antibacterial drugs around 2023. The total care sector is shown where available. A consumption level below 10 DID is coloured dark green, a level between 10 and 15 DID is coloured light green, a moderate level between 15 and 20 DID is coloured yellow, a high level between 20 and 25 DID is coloured orange and a very high level exceeding 25 DID is coloured red. Countries where no data was available are grey-coloured. The map was created using mapchart.net

In order to gain an initial understanding of the trends and characteristics of each country, the development over the last few years is observed. After an assessment of the volume of consumption from the early years to the present, countries are compared in terms of their level of consumption and its changes. As data became available at different times, it was decided to make the comparison with data points from 2000/2003 to 2021/2023 to ensure a robust comparison. The aim was to include as many years as possible, but to avoid misleading consumption data from different years, as there is likely to be an underlying trend, both nationally and internationally. Table 3, Figs. 2 and 3 depict the development of consumption.

**Table 3:**
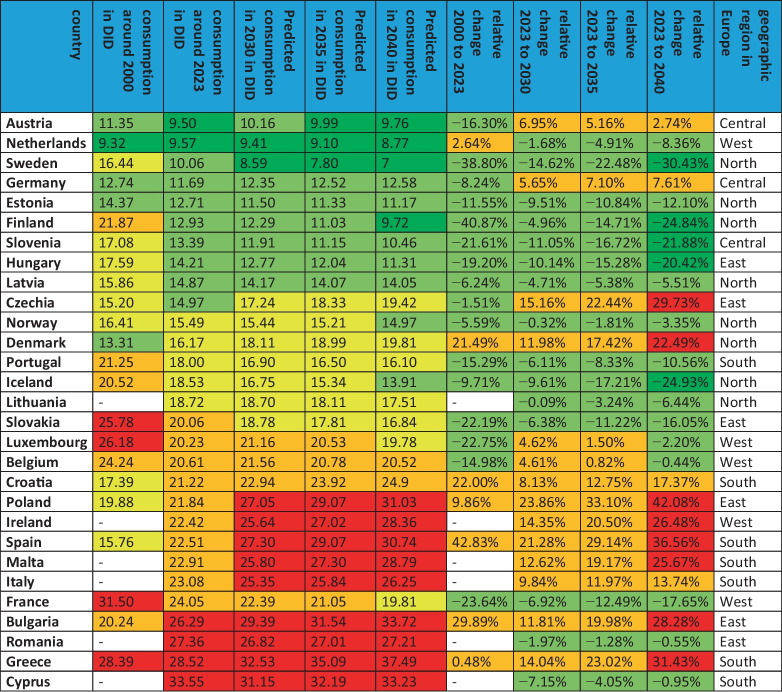
Consumption of antibacterial drugs for European countries around 2000 and 2023 and projections for 2030, 2035 and 2040. The volume of consumption is expressed in DID (DDD prescriptions per 1000 inhabitants per day), favouring the total care sector (in the absence of data, alternatively the community sector). In addition, the sorted geographical region is indicated. Countries are sorted in ascending order of consumption volume in 2021/2023. DID volumes are highlighted in colour. Dark green are countries with less than 10 DID, light green countries with 10 and 15 DID. Yellow are countries with a moderate level of consumption between 15 and 20 DID, orange are countries with a DID between 20 and 25. Red are countries with a DID above 25. Relative changes are also highlighted. A large decrease of more than −20% is shown in dark green, while a decrease between −20% and +−0% is shown in light green. An increase between +−0% and +20% is orange, while a strong increase above +20% is red

Countries where consumption decreased from the first to the last available data were Spain (−43.1%), Finland (−40.9%), Sweden (−38.8%), France (−23.6%), Luxembourg (−22.8%), Slovakia (−22. 2%), Slovenia (−21.6%), Hungary (−19.2%), Austria (−16.3%), Portugal (−15.0%), Poland (−15.3%), Belgium (−15.0%), Estonia (−11.6%), Iceland (−9.7%), Germany (−8,2%), Latvia (−6.2%) and Norway (−5.6%). Consumption was more or less stable in Czechia (−1.5%), Greece (+0.5%) and the Netherlands (+2.64%). Countries with increasing consumption are Poland (+9.9%), Denmark (+21.5%), Croatia (+22.0%), Bulgaria (+29.9%) and Spain (+42,8%). For some countries, data were available only after a few years, so that the analysis of relative changes was excluded. These include Lithuania, Romania, Malta, Italy, Cyprus and Ireland.

Around 2000, very low levels of use, defined as DIDs below 15, are reported for the Netherlands (9.3 DID), Austria (11.4 DID), Germany (12.7 DID), Denmark (13.3 DID) and Estonia (14.4 DID). Low consumption, with DIDs between 15 and 20, includes Czechia (15.2 DIDs), Spain (15.8 DID), Latvia (15.9 DIDs), Norway and Sweden (16.4 DIDs), Slovenia (17.1 DIDs), Croatia (17.4 DIDs), Hungary (17.6 DIDs) and Poland (19.9 DID). Moderate consumption, with DIDs between 20 and 25, is found in Bulgaria (20.2 DID), Iceland (20.5 DID), Portugal (21.2 DID), Finland (21.9 DID) and Belgium (24.2 DID). A high consumption volume of more than 25 DID is found in Slovakia (25.8 DID), Luxembourg (26.2 DID), Greece (28.4 DID) and France (31.5 DID).

Around 2023, low levels of consumption are reported for Austria (9.5 DID), the Netherlands (9.6 DID), Sweden (10.1 DID), Germany (11.7 DID), Estonia (12.7 DID), Finland (12.9 DID), Slovenia (13.4 DID), Hungary (14.2 DID), Latvia (14.9 DID) and Czechia (15.0 DID). Moderate consumption levels are present in Norway (15.5 DID), Denmark (16.2 DID), Portugal (18.0 DID), Iceland (18.5 DID) and Lithuania (18.7 DID). A high consumption level is depicted for Slovakia (20.1 DID), Luxembourg (20.2 DID), Belgium (20.6 DID), Croatia (21.2 DID), Ireland (22.4 DID), Spain (22.5 DID), Malta (22.9 DID), Italy (23.1 DID) and France (24.1 DID). Very high levels of consumption are recorded in Bulgaria (26.3 DID), Romania (27.4 DID), Greece (28.5 DID) and Cyprus (33.6 DID).

In summary, 16 countries reported a decrease and 7 countries an increase in the use of antibacterial drugs. For 6 countries, it was not possible to assess past trends because no data were published for years around 2000. However, these countries published data for the year around 2023, allowing an assessment of the current level of consumption. A low level of consumption exists for countries around 2000 and for 5 countries around 2023. A medium volume of consumption includes 9 countries around 2000 and 5 countries around 2023. A high volume is present for 9 countries around 2000 and for 14 countries in 2023.

### Development of antibacterial drug consumption in the hospital sector in European countries

**Fig. 4:**
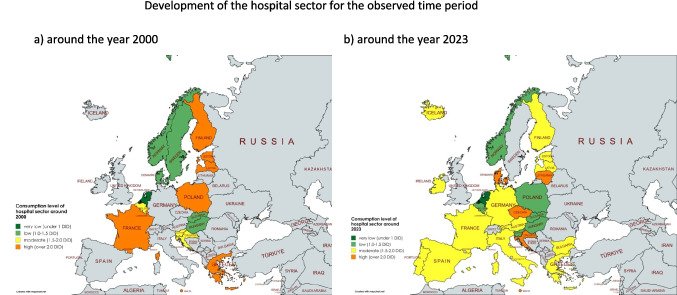
Consumption level of the hospital sector around 2000 and 2023. In (a) the consumption around the year 2000 is shown, while in (b) the consumption around the year 2023 is shown. A consumption level below 1 DID is coloured dark green, a level between 1.0 and 1.5 DID is coloured light green, a moderate level between 1.5 and 2.0 DID is coloured yellow, and a high level over 2.0 DID is coloured orange. Countries where no data was available are grey-coloured. The map was created using mapchart.net

A sufficient number of data points for both the hospital and the community sectors are available for 28 countries to assess trends and compare the sectors, including all countries except Cyprus. The following section analyses the trend over the whole period available and the trend in recent years. As the number of years available varies between countries, the overall trend gives an insight into the trend in a single country, but is not well suited to comparisons between countries. To allow comparisons of trends, consumption levels for the years around 2000 and 2023 are compared (Table 4 and Fig. 4).

**Table 4:**
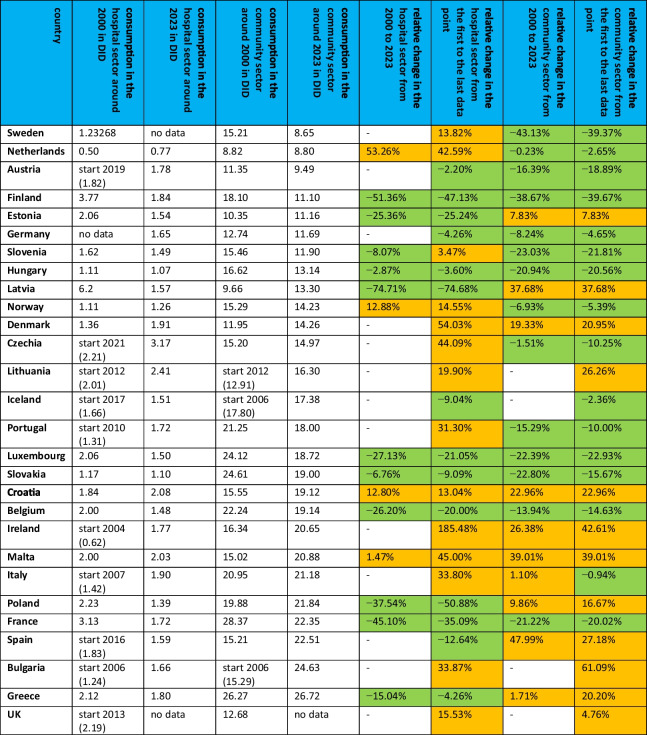
Comparison of the development of the hospital and community sectors. Consumption in DID around 2000 and 2023 is shown, as well as the relative changes for each sector between 2000 and 2023 and from the first to the last data point. If data collection started later than 2000, the starting year is mentioned and the respective DID is given in parentheses. A decrease is highlighted in green and an increase in orange. The countries are sorted in ascending order of consumption in the community sector around 2023

Looking at the whole period for each country, including all available data, decreases are recorded for Latvia (−74.7%), Poland (−50.9%), Finland (−47.1%), France (−35.1%), Estonia (−25.2%), Luxembourg (−21.1%), Belgium (−20.0%), Spain (−12.6%), Slovakia (−9.1%), Iceland (−9.0%), Greece (−4.3%), Germany (−4.3%), Hungary (−3.6%) and Austria (−2.2%). An upward trend is observed in Slovenia (+3.5%), Croatia (+13.0%), Sweden (+13.8%), Norway (+14.6%), the UK (+15.5%), Lithuania (+19.9%), Portugal (+31.3%), Italy (+33.8%), Bulgaria (+33.9%), the Netherlands (+42.6%), Czechia (+44.1%), Malta (+45.0%), Denmark (+45.0%) and Ireland (+185.5%).

From around 2000 to 2023, a downward trend is observed for Latvia (−74.7%), Finland (−51.4%), France (−45.1%), Poland (−37.5%), Luxembourg (−27.1%), Belgium (−26.2%), Estonia (−25.4%), Greece (−15.0%), Slovenia (−8.1%), Slovakia (−6.8%) and Hungary (−2.9%). On the other hand, an upward trend can be observed in Malta (+1.5%), Croatia (+12.8%), Norway (+12.9%) and the Netherlands (53.26%). For several countries, data were available several years later than 2000 and are therefore only analysed for the whole period.

As for the level of use in the hospital sector around 2000, low levels (below 1.5 DID) are reported by the Netherlands (0.5 DID), Hungary (1.1 DID), Norway (1.1 DID), Slovakia (1.2 DID), Sweden (1.2 DID) and Denmark (1.5 DID). Consumption levels considered to be moderate (between 1.5 and 2.0 DID) include Slovenia (1.6 DID), Croatia (1.8 DID) and Belgium (2.0 DID). Consumption levels considered to be high (above 2.0 DID) are reported by Malta (2.0 DID), Estonia (2.1 DID), Luxembourg (2.2 DID), Greece (2.1 DID) and Poland (2.2 DID). Very high levels of consumption (over 2.5 DID) are found in France (3.1 DID), Finland (3.8 DID) and Latvia (6.2 DID).

As consumption levels were not available for some years in 2000, due to the later start of data collection, they cannot be compared with the other countries, but provide an insight into individual levels and trends. Ireland (0.6 DID in 2004), Bulgaria (1.2 DID in 2006), Portugal (1.3 DID in 2010) and Italy (1.4 DID in 2007) are considered to have low levels of consumption. A medium level is present for Iceland (1.7 DID in 2016), Spain (1.8 DID in 2016) and Austria (1.8 DID in 2019). Consumption is considered to be high in Lithuania (2.0 DID in 2012), the UK (2.2 DID in 2019) and Czechia (2.2 DID in 2021).

Around 2023, the level of consumption in the hospital sector is low for the Netherlands (0.8 DID), Hungary (1.1 DID), Slovakia (1.1 DID), Norway (1.3 DID), Poland (1.4 DID), Belgium (1.5 DID) and Slovenia (1.5 DID). A medium level is reported by Luxembourg (1.5 DID), Iceland (1.5 DID), Estonia (1.5 DID), Latvia (1.6 DID), Spain (1.6 DID), Bulgaria (1.7 DID), France (1. 7 DID), Portugal (1.7 DID), Ireland (1.8 DID), Austria (1.8 DID), Greece (1.8 DID), Finland (1.8 DID), Italy (1.9 DID) and Denmark (1.9 DID). High levels of use are found in Malta (2.0 DID), Croatia (2.1 DID), Lithuania (2.4 DID) and Czechia (3.2 DID). No data were published for Sweden and the UK around 2023.

In summary, 14 countries reported a decrease in the hospital sector from the first to the last data point. It is noteworthy that the increasing or decreasing trend over the whole period analysed continues in most countries, with the exception of Slovenia, which increased from the first to the last data point but decreased from 2000 to 2023. In contrast, 14 countries increased their consumption over the whole period. Consumption levels around 2000 are considered low for 6 countries, moderate for 3 countries and high for 8 countries. The consumption levels of 11 countries were not compared because data collection started in later years, but were categorised into levels individually. Around the year 2023, consumption levels were low for 7 countries, moderate for 15 countries and high for 4 countries, while no data were available for 2 countries.

### Development of antibacterial drug consumption in the community sector in European countries

**Fig. 5:**
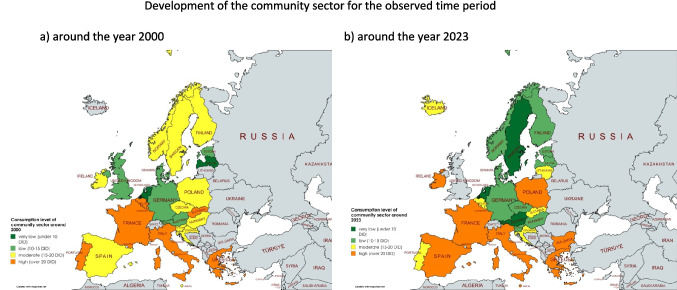
Consumption level of the community sector around 2000 and 2023. In (a) the consumption around the year 2000 is shown, while in (b) the consumption around the year 2023 is shown. A consumption level below 10 DID is coloured dark green, a level between 10 and 15 DID is coloured light green, a moderate level between 15 and 20 DID is coloured yellow, and a high level over 20 DID is coloured orange. Countries where no data was available are grey-coloured. The map was created using mapchart.net

A sufficient number of data points for both the hospital and the community sectors are available for 28 countries to assess trends and compare the sectors, including all countries except Cyprus. The following section analyses the trend over the whole period available and the trend in recent years. As the number of years available varies between countries, the overall trend gives an insight into the trend in a single country, but is not well suited to comparisons between countries. In order to compare the trends, the consumption levels for the years around 2000 and 2023 are compared (Table 4, Fig. 5).

Between 2000 and 2023, community consumption is projected to decrease in Sweden (−43.1%), Finland (−38.7%), Slovenia (−23.0%), Slovakia (−22.8%), Luxembourg (−22.4%), France (−21.2%), Hungary (−20.9%), Austria (−16.4%), Portugal (−15.3%), Belgium (−13.9%), Germany (−8.2%), Norway (−7.0%), Czechia (−1.5%) and the Netherlands (−0.2%). Increases were recorded in Italy (+1.1%), Greece (+1.7%), Estonia (+7.8%), Poland (+9.9%), Denmark (+19.3%), Croatia (+23.0%), Ireland (+26.4%), Latvia (+37.7%), Malta (+39.0%) and Spain (+48.0%). Data collection started later for Iceland, Bulgaria and Lithuania, while the UK did not provide any recent data.

A downward trend from the first to the latest data was observed in Finland (−39.7%), Sweden (−39.4%), Luxembourg (−22.9%), Slovenia (−21.8%), Hungary (−20.6%), France (−20.0%), Austria (−18. 9%), Slovakia (−15.7%), Belgium (−14.6%), Czechia (−10.3%), Portugal (−10.0%), Norway (−5.4%), Germany (−4.7%), the Netherlands (−2.7%), Iceland (−2.4%) and Italy (−0.9%). There were increases in the UK (+4.8%), Estonia (+7.8%), Poland (+16.7%), Greece (+20.2%), Denmark (+21.0%), Croatia (+23.0%), Lithuania (+26.3%), Spain (+27.2%), Latvia (+37.7%), Malta (+39.0%), Ireland (+42.6%) and Bulgaria (+61.1%).

Concerning the level of consumption in the community sector around 2000, a low level (less than 15 DID) is reported for the Netherlands (8.8 DID), Latvia (9.7 DID), Estonia (10.4 DID), Austria (11.4 DID), Denmark (12.0 DID), the UK (12.7 DID) and Germany (12.7 DID). A medium level (15–20 DID) is reported by Malta (15.0 DID), Czechia (15.2 DID), Sweden (15.2 DID), Spain (15.2 DID), Norway (15.3 DID), Slovenia (15.5 DID), Croatia (15.6 DID), Ireland (16.4 DID), Hungary (16.2 DID), Finland (18.1 DID) and Poland (19.9 DID). A high level of more than 20 DID is recorded for Italy (21.0 DID), Portugal (21.3 DID), Belgium (22.2 DID), Luxembourg (24.1 DID), Slovakia (24.6 DID), Greece (26.3 DID) and France (28.4 DID). Data were not available for Bulgaria, Iceland and Lithuania.

As the level of consumption is not available for some years in 2000, because recording started later, it cannot be compared with the other countries, but it does give an insight into the individual level and trend. Lithuania is considered to have a low level (12.9 DID in 2012), while Bulgaria (15.3 DID in 2006) and Iceland (17.8 DID in 2006) have moderate levels.

Around 2023, low levels (below 15 DID) are found in Sweden (8.7 DID), the Netherlands (8.8 DID), Austria (9.5 DID), Finland (11.1 DID), Estonia (11.2 DID), Germany (11.7 DID), Slovenia (11.9 DID), Hungary (13.1 DID), Latvia (13.3 DID), Norway (14.2 DID), Denmark (14.3 DID) and Czechia (15.0 DID). Moderate levels between 15 and 20 DID are found in Lithuania (16.3 DID), Iceland (17.4 DID), Portugal (18.0 DID), Luxembourg (18.7 DID), Slovakia (19.0 DID), Croatia (19.1 DID) and Belgium (19.1 DID). High levels of over 20 DID are found in Ireland (20.7 DID), Malta (20.9 DID), Italy (21.2 DID), Poland (21.8 DID), France (22.4 DID), Spain (22.5 DID), Bulgaria (24.6 DID) and Greece (26.7 DID). No data were available for the UK.

In summary, 16 countries reported a decrease in the community sector from the first to the last data point. It is noteworthy that the increasing or decreasing trend over the whole period analysed continues in most countries, with the exception of Italy, which decreased from the first to the last data point, but increased from 2000 to 2023. On the other hand, 12 countries increased their consumption over the whole period. Consumption levels around 2000 are considered low for 7 countries, moderate for 11 countries and high for 7 countries. Consumption levels for 3 countries were not compared because data collection started in later years, but were categorised into levels individually. Around the year 2023, consumption levels were low for 12 countries, moderate for 7 countries and high for 8 countries, while no data were available for one country.

### Prediction of consumption in the total care sector until 2040: European countries with an increasing trend

**Fig. 6:**
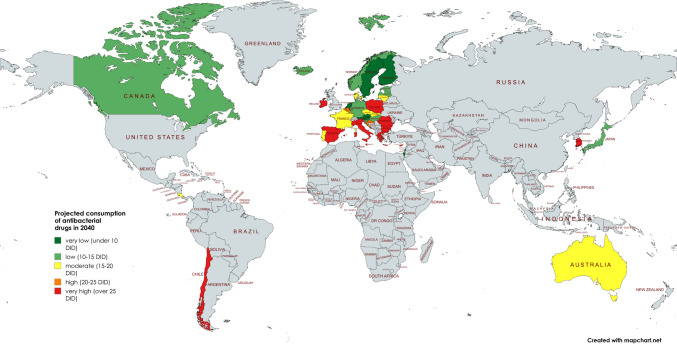
Projection of the consumption of antibacterial drugs in 2040. The total care sector is shown where available. A consumption level below 10 DID is coloured dark green, a level between 10 and 15 DID is coloured light green, a moderate level between 15 and 20 DID is coloured yellow, a high level between 20 and 25 DID is coloured orange and a very high level exceeding 25 DID is coloured red. Countries where no data was available are grey-coloured. The map was created using mapchart.net

The ARIMA(1,1,1) model was used to project all European countries, except the UK, for the years following the latest reported data up to 2040. An increasing trend is projected for 12 of the 28 countries. For some of these, the confidential interval offers the possibility of a decline, but for others this is not the case. The relative increases and projected consumption volumes are shown below, in Table 3 and in Fig. 6.

Increases are projected for Poland with +42.1% (reaching 31.0 DID in 2040), Spain with +36.6% (30.7 DID), Greece with +31.4% (37.5 DID), Czechia with +29.7% (19.4 DID), Bulgaria with +28.3% (33.7 DID), Malta with +26.5% (28.8 DID), Denmark with +25.7% (19.8 DID), Croatia with +17.4% (24.9 DID), Italy with +13.7% (26.3 DID) and Germany with +7.6% (12.6 DID).

For 8 countries, an opposite trend could be possible, expressed by a decrease within their LCL. These are Austria with −68.9% (LCL of 3.0 DID in 2040), Bulgaria with −40.4% (15.7 DID), Croatia with −45.5% (11.6 DID), Czechia with −23.1% (11.0 DID), Denmark with −20.4% (12.9 DID), Germany with −27.9% (8.4 DID), Greece with −16.4% (15.3 DID), Italy with −51.0% (11.3 DID) and Spain with −33.0% (15.1 DID).

For 4 countries, there is no possibility of a decrease in the respective LCL. These include Czechia with +21.1% (LCL of 18.1 DID in 2040), Ireland with +15.4% (25.9 DID), Malta with +6.7% (24.4 DID) and Poland with +30.6% (28.5 DID).

The range of the UCL and LCL, which gives an indication of the uncertainty of the forecast, is considered narrow or moderate for countries with an increasing trend. A narrow range, defined as less than 100%, is shown for Czechia (range 13.2%), Denmark (70.0%), Germany (65.9%), Ireland (17.5%), Malta (30.2%) and Poland (16.1%). A moderate range, defined by a relative variability between 100 and 200%, includes Bulgaria (107.0%), Croatia (107.1%), Greece (110.3%), Italy (113.9%) and Spain (101.9%).

In terms of consumption levels in 2040, 2 countries have a predicted level that can be considered as low (less than 15 DID), including Austria and Germany. A moderate level (15–20 DID) is predicted for 2 countries, including Denmark and Czechia. A high level (over 20 DID) is forecast for 8 countries, including Poland, Spain, Greece, Bulgaria, Ireland, Malta, Croatia and Italy.

In summary, about one-third of the countries analysed are predicted to experience an increase by 2040. For many of them, the ARIMA(1,1,1) model prediction offers the possibility of a decrease. The range between LCL and UCL, which expresses the uncertainty of the forecasts, is very narrow in most cases. 4 of the countries with an upward trend are considered to have a low or moderate level of consumption, while the other 8 will have a high level. In order to assess the reliability of these forecasts, further analysis of the fit metrics is necessary.

### Prediction of consumption in the total care sector until 2040: European countries with a decreasing trend

For 16 countries, the ARIMA(1,1,1) model predicts a decline by 2040 (Table 3, Fig. 6). As for the countries with an upward trend, there are differences in the possibility of a reverse trend, as indicated by the value of the UCL.

Declines are projected for Belgium with −0.5% (reaching 20.5 DID in 2040), Romania with −0.6% (27.2 DID), Cyprus with −1.0% (33.2 DID), Luxembourg with −2.2% (19.8 DID), Norway with −3.4% (15.1 DID), Latvia with −5.5% (14.1 DID), Lithuania with −6.4% (17.5 DID), the Netherlands with −8.4% (8.8 DID), Portugal with −10.6% (16.1 DID), Estonia with −12.1% (11.2 DID), Slovakia with −16.1% (16.8 DID), France with −17.7% (19.8 DID), Hungary with −20.4% (11.3 DID), Slovenia with −21.9% (10.5 DID), Finland with −24.8% (9.7 DID), Iceland with −24.9% (13.9 DID) and Sweden with −30.4% (7.0 DID).

An upward trend is possible for almost all countries on the basis of their UCL. These include Cyprus with an UCL up to +21.7% (reaching 40.8 DID in 2040), Belgium with +39.7% (28.8 DID), Estonia with +1.2% (12.9 DID), Finland with +27.5% (16.5 DID), France with +1.1% (24.3 DID), Lithuania with +77.0% (33.1 DID), Luxembourg with +32.6% (26.8 DID), the Netherlands with +21. 2% (11.6 DID), Norway with +9.2% (16.9 DID), Portugal with +4.2% (18.8 DID), Romania with +13.6% (31.1 DID), Slovakia with +2.4% (20.5 DID) and Sweden with +29.8% (13.1 DID).

Only 3 countries are excluded from a downward trend by the forecast model. These are Hungary with an UCL of −3.1% (13.8 DID), Iceland with −13.6% (16.0 DID) and Slovenia with −1.7% (13.2 DID).

In most cases, the range between LCL and UCL, which expresses the uncertainty of the forecasts, is narrow. There are only a few countries where the range is considered to be moderate or even large. For 12 countries the range is less than 100%, including Belgium (range 80.6%), Cyprus (45.7%), Estonia (30.3%), France (45.7%), Hungary (43.5%), Iceland (30.2%), Luxembourg (71.1%), Latvia (54.2%), the Netherlands (64.4%), Norway (25.9%), Portugal (33.1%), Romania (28.5%), Slovenia (51.6%) and Slovakia (44.0%). A moderate range between 100 and 200% is shown by the three countries Finland (139.3%), Lithuania (178.4%) and Sweden (173.1%).

In terms of consumption levels in 2040, 9 countries are projected to have low levels (less than 15 DID), including Sweden, Iceland, Finland, Slovenia, Hungary, Estonia, the Netherlands, Latvia and Norway. A moderate level (15–20 DID) is predicted for 5 countries, including France, Slovakia, Portugal, Lithuania and Luxembourg. A high level (over 20 DID) is predicted for 3 countries, including Cyprus, Romania and Belgium.

In summary, around two-thirds of the countries analysed are projected to experience a decline by 2040. However, for most of them the LCL allows for the possibility of an increase, although in many cases only a slight decrease is possible. The relative range between LCL and UCL, which expresses the uncertainty of the forecast, is in most cases very narrow, although some countries show a moderate or even wide range. Further analysis of the fit metrics is necessary to assess the reliability of each forecast.

### Development of antibacterial drug consumption in non-European countries

The OECD provided sufficient data for seven countries. These are Australia, Canada, Costa Rica, Chile, Israel, Korea and Japan. All these countries are OECD members. The available period is between 1990 and 2023. Details can be found in Table 5, Figs. 2 and 3.

**Table 5:**
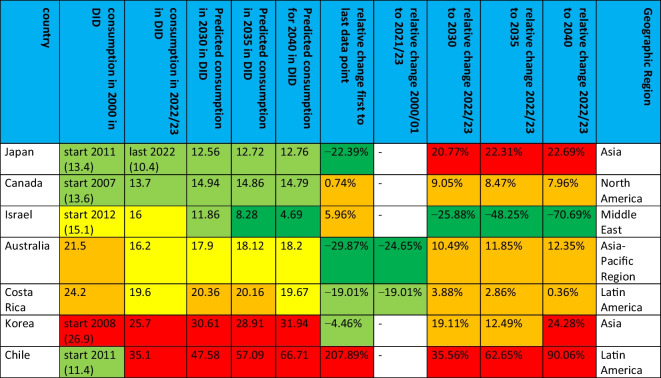
Consumption of antibacterial drugs for non-European OECD countries around 2000 and 2023 and projections for 2030, 2035 and 2040. The volume of consumption is expressed in DID (DDD prescriptions per 1000 inhabitants per day), favouring the total care sector (in the absence of data, alternatively the community sector). In addition, the sorted geographical region is indicated. If data collection started later than 2000, the starting year is mentioned and the respective DID is given in parentheses. Countries are sorted in ascending order of consumption volume in 2021/2023. DID volumes are highlighted in colour. Dark green are countries with less than 10 DID, light green countries with 10–15 DID. Yellow are countries with a moderate level of consumption between 15 and 20 DID, orange are countries with a DID between 20 and 25. Red are countries with a DID above 25. Relative changes are also highlighted. A large decrease of more than −20% is shown in dark green, while a decrease between −20% and + −0 % is shown in light green. An increase between + −0 % and +20% is orange, while a strong increase above +20% is red

From the first to the last data point, there is a downward trend for four countries: Australia (−29.9%), Japan (−22.4%), Costa Rica (−19.0%) and Korea (−4.5%). On the other hand, an increasing trend is observed for the three countries Canada (+0.7%), Israel (+6.0%) and Chile (+207.9%). In terms of relative change between 2000 and 2023, data were available for two countries, Costa Rica and Australia, which both show a decrease of −19.0% and −24.7% respectively. The other five countries started to report in later years.

In terms of levels of use around 2000, the two countries with reported data have very high levels of consumption, including Australia with 21.5 DID and Costa Rica with 24.2 DID. The other four countries started reporting later. Low levels under 15 DID are found in Canada (13.6 DID in 2007), Chile (11.4 DID in 2011) and Japan (13.4 DID in 2011). Moderate levels between 15–20 DID are considered for Israel (15.1 DID in 2012) and a high level exceeding 25 DID for Korea (26.9 DID in 2008).

Around 2023, low levels are found for Canada (13.7 DID) and Japan (10.4 DID in 2020). Moderate levels are shown for Israel (16.0 DID), Australia (16.2 DID) and Costa Rica (19.6 DID). High levels are reported for Korea (25.7 DID) and Chile (35.1 DID).

In summary, 2 countries reported a decrease between 2000 and 2023, while no data were available for 5 countries. Looking at the longest period available for each country, 4 countries reported a decrease and 3 an increase. In an outstanding way, Chile experienced a strong increase of more than +200%. A low level of consumption at the first data point exists for 3 countries and for 2 countries around 2023. A medium volume of consumption is present for 1 country at the beginning and for 3 countries around 2023. A high volume is present for one country at the start and for 2 countries around 2023.

### Prediction of consumption for non-European countries

By 2040, the ARIMA(1,1,1) model predicts an increase for almost all the non-European countries analysed, with the exception of Israel. As for the European countries, there are differences in the possibility of a reversal of the trend, as indicated by the LCL and UCL. Detailed information can be found in Table 5, Figs. 3 and 6.

Israel's decrease will be −70.7% between 2022 and 2040, reaching 4.7 DID. The model excludes the possibility of an increasing trend, as indicated by the UCL of −33.5%.

An increase is projected for Australia (+12.4%, reaching 18.2 DID), Canada (+8.0%, reaching 14.8 DID), Chile (+90.1%, reaching 66.7 DID), Costa Rica (+0.4%, reaching 19.7 DID), Japan (+22.7%, reaching 12.8 DID) and Korea (+24.3%, reaching 31.9 DID). It is striking that some of the countries with an upward trend will increase only very slightly, such as Costa Rica and Canada, while others, such as Chile, are expected to increase dramatically. Five of these countries show the possibility of a decrease in LCL. These are Australia (−11.0%, LCL of 14.4 DID), Canada (−100.0%, 0.0 DID), Costa Rica (−66.9%, 26.4 DID), Japan (−85.7%, 12.8 DID) and Korea (−16.1%, 21.6 DID). Only for Chile (+44.1%, 32.3 DID) does the model see the possibility of an increase.

The range of the UCL and LCL expresses the uncertainty of the forecasts. There are narrow, moderate and wide ranges. A narrow range under 100% is shown for Australia (41.5%), Chile (48.4%) and Korea (65.0%). A moderate range between 100–200% is shown by Costa Rica (134.0%) and Japan (178.5%). A wide range above 200% is shown for Israel (226.9%) and Canada (230.4%).

In terms of consumption levels in 2040, three countries are considered to have low levels. These are Israel (4.7 DID), Japan (12.8 DID) and Canada (14.8 DID). A moderate level is predicted for Australia (18.1 DID). In contrast, high levels are predicted for Costa Rica (20.2 DID), Korea (32.0 DID) and Chile (66.7 DID).

In summary, six of seven countries analysed are expected to experience an increase by 2040. For most of them, however, the LCL allows for the possibility of an increase. The exceptions are Chile, with a strong increase, and Israel, the only country with a decrease. The relative range between LCL and UCL varies from narrow to wide. Further analysis of the fit metrics is necessary to assess the reliability of each forecast.

## Discussion

### Reliability of forecasts

Assessing the reliability of the forecasts for the total care sector to 2040 requires a comprehensive evaluation of the ability of the models to fit the data. This analysis considers fit metrics, focusing on stationary R-squared, R-squared and MAPE, categorised as good, moderate or poor. For a conclusive assessment of reliability, the fit metrics are combined with the plausibility of the forecasts and the range of upper (UCL) and lower control limits (LCL).

In countries with increasing trends, the fit metrics are generally moderate, although there are exceptions with good or poor fits. For example, Austria, Bulgaria, Croatia, Czechia, Denmark, Germany, Italy, Malta, Poland, Spain, Chile and Costa Rica are classified as having a moderate fit. On the other hand, Greece has a poor fit, while Australia, Canada, Japan and Korea are considered to have a good fit. Detailed assessments for parameters and countries are given in Tables 6, 7 and 8.

**Table 6:**
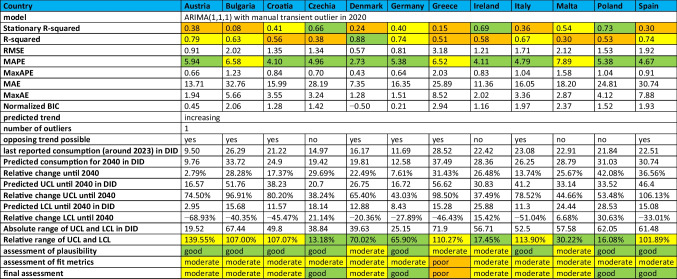
Prediction of the ARIMA(1,1,1) model for European countries with an increasing trend. Fit metrics, predictions until 2040 and relative changes are shown. The reliability of the model is also assessed. Characteristics indicating a good fit are coloured green, those indicating a moderate fit are coloured yellow and those indicating a poor fit are coloured orange

**Table 7:**
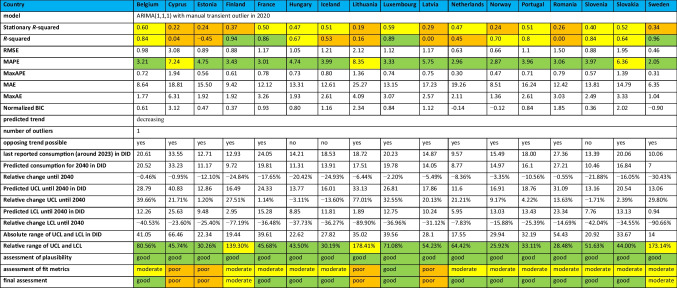
Prediction of the ARIMA(1,1,1) model for European countries with a decreasing trend. Fit metrics, predictions until 2040 and relative changes are shown. The reliability of the model is also assessed. Characteristics indicating a good fit are coloured green, those indicating a moderate fit are coloured yellow and those indicating a poor fit are coloured orange

**Table 8:**
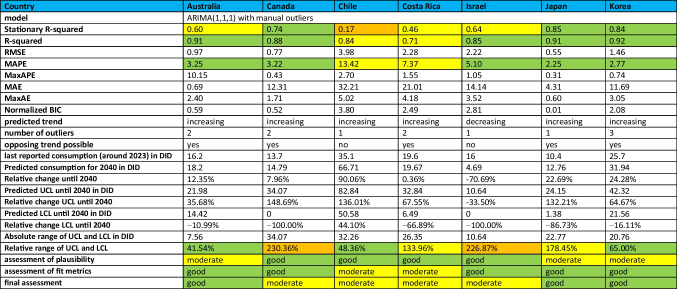
Prediction of the ARIMA(1,1,1) model for non-European OECD countries. Fit metrics, predictions until 2040 and relative changes are shown. The reliability of the model is also assessed. Characteristics indicating a good fit are coloured green, those indicating a moderate fit are coloured yellow and those indicating a poor fit are coloured orange

In the countries with a declining trend, most of the fit measures also fall into the moderate category, with a few exceptions that are classified as good or poor. Countries with a moderate fit include Belgium, Finland, France, Hungary, Iceland, the Netherlands, Norway, Portugal, Romania, Slovenia, Slovakia and Sweden. Luxembourg and Israel have good fit metrics, while Cyprus, Estonia, Lithuania and Latvia are considered to have poor fit metrics.

Goodness of fit reflects the ability of the model to capture the evolution of the data. Robust models show high R-squared and stationary *R*-squared values, coupled with low MAPE and BIC values. Conversely, poor models show low *R*-squared values and higher MAPE and RMSE values, indicating limited accuracy in capturing the dynamics of the data. Poor model fits are often due to poor data quality, limited data availability or significant fluctuations. Dynamic or unstable trends introduce additional uncertainty as external influences may change over time. A good model fit increases the reliability of forecasts, while poor fit metrics reduce the confidence in forecasts (Bindel and Seifert 2025b).

The final reliability assessment integrates the fit metric categories, the relative range of the UCL and LCL, and subjective plausibility assessments. Plausibility assesses whether the 2040 projections are consistent with observed trajectories. The final assessment classifies projections as good, moderate or poor. Good ratings suggest accurate projections. Moderate scores suggest correct trends, but acknowledge potential variation in the exact values. Poor scores question the direction of the forecast or include cases where robust models could not be built.

Countries with good reliability include Belgium, Czechia, France, Germany, Hungary, Iceland, Luxembourg, the Netherlands, Norway, Portugal, Romania, Slovenia, Slovakia, Spain, Australia, Japan and Korea. These countries have narrow or moderate confidence intervals and reasonable forecasts that are in line with past developments.

Moderate reliability applies to Austria, Bulgaria, Croatia, Denmark, Finland, Ireland, Italy, Malta, Sweden, Canada, Chile, Costa Rica and Israel. These countries have moderate fit metrics and a moderate range or good fit metrics with a wide range. Their plausibility ranges from good to moderate. The projected trends in these countries are generally accurate, although the magnitude of future developments may be more uncertain, as indicated by the wider confidence interval.

Low confidence is assigned to countries with poor fit metrics, including Cyprus, Estonia, Greece, Lithuania and Latvia. While these models may appear plausible with narrow UCL and LCL ranges, models that fit the actual trajectory poorly may still produce plausible-looking but unreliable forecasts. Such models should be interpreted with caution.

In summary, the fit metrics are considered good for six countries, moderate for 25 countries and poor for 5 countries. The confidence interval, expressed by the range of the UCL and LCL, is narrow (below 100%) for 23 countries, moderate (100–200%) for 11 countries and (exceeding 200%) for two countries. Overall, the forecast reliability is good for 17 countries, moderate for 13 countries and poor for 5 countries. The ability to produce reliable forecasts depends on the quality and consistency of the data. Low data availability, significant fluctuations, time breaks or external influences such as the COVID pandemic are reflected in poorer fit metrics.

### Relationship between prescription level and trend

An analysis of past consumption levels and projected developments shows a clear pattern: countries with low levels of consumption tend to maintain or further reduce them, while countries with high levels of consumption continue to show high levels of consumption and, in some cases, increases (Tables 3 and 5). This suggests a growing divergence in consumption trends, with countries polarising into very low and exceptionally high consumption groups, resulting in more extreme values than in the past.

**Fig. 7:**
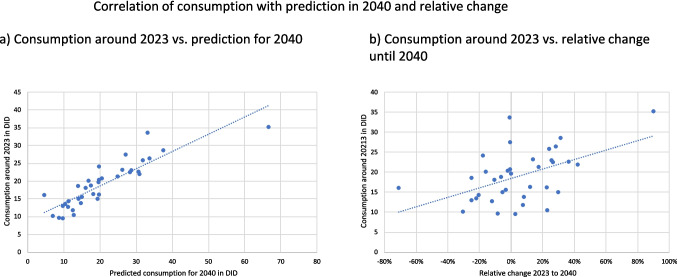
Correlation panels for the relationship between the consumption of antibacterial drugs around 2023 and (a) the predicted consumption for 2040, as well as (b) the relative change between 2023 and 2040. A regression line is plotted in each graph

In order to quantify this behaviour, a correlation analysis was carried out, examining consumption levels in 2023 compared to projected levels in 2040 and the relative change between 2023 and 2040. Significant positive correlations were observed in both cases (Table 9, Fig. 7), highlighting the relationship between current and future consumption and the relative change by 2040. The correlation coefficient between 2023 consumption and relative change was moderate (0.49), while the correlation between 2023 and 2040 consumption was strong (0.88).

**Table 9:**
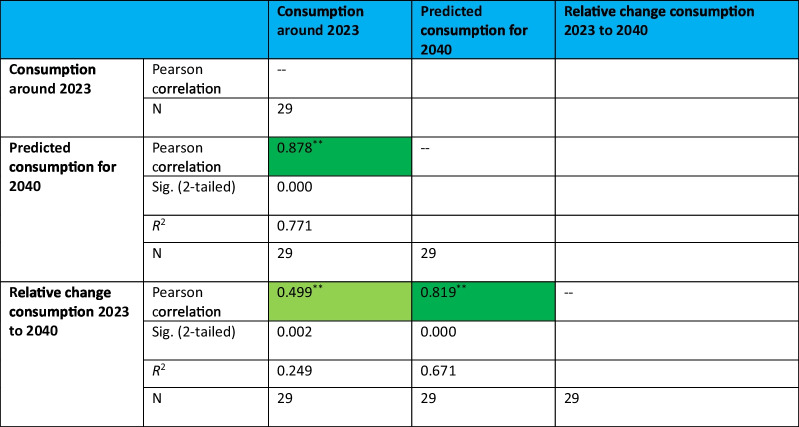
Correlation between consumption around 2023 and the forecast for 2040, as well as for the relative change between 2023 and 2040. A significant correlation at the 0.01 level is highlighted in dark green, while a significant correlation at the 0.05 level is highlighted in light green

** Correlation is significant at the 0.01 level (2-tailed) * Correlation is significant at the 0.05 level (2-tailed)

Countries with higher consumption levels in 2023 tend to show smaller decreases or even increases by 2040 compared to countries with lower consumption levels. For example, Sweden and the Netherlands, which are characterised by historically low consumption levels, show minimal changes. Conversely, Greece and Cyprus, which have always had high levels of consumption, maintain these trends into the future. This pattern is consistent with the likelihood that countries with low current consumption will implement more effective public health interventions aimed at further reducing antibacterial drug use (Andersson et al. 2022; Mölstad et al. 2017; De Greef et al. 2021). On the other hand, countries with high levels of consumption 2023 are often less successful in implementing such interventions, which may explain the persistence or increase in their consumption levels (ECDC 2024a; Karakonstantis and Kalemaki 2019; Spernovasilis and Tsioutis 2024). In addition, countries with already low consumption have limited potential for further reductions as their consumption is likely to have plateaued, contributing to the moderate correlation with relative change. External factors such as health policies, cultural behaviours and levels of bacterial resistance play a dominant role in shaping relative changes in consumption (Bindel and Seifert 2024b, 2025a; Karakonstantis and Kalemaki 2019; Spernovasilis and Tsioutis 2024). Thus, while baseline consumption levels are important, they reflect the outcomes of these external factors and implemented policies rather than being the sole drivers of change.

The strong correlation between higher consumption in 2023 and higher predicted consumption in 2040 underlines the predictive value of historical patterns. Countries with persistently high levels of consumption often have structural factors, such as cultural practices or challenges related to antimicrobial resistance, that maintain such levels. While the predictive model reinforces this relationship by relying on historical data, countries with low current consumption reflect established restrictive policies or rational prescribing behaviours that are expected to persist, resulting in consistently lower consumption levels in 2040.

In summary, these correlations imply that low consumption in 2023 does not lead directly to a sharp decline in 2040, but to a lower absolute level of consumption in 2040. The moderate relationship with relative change highlights that changes in antibacterial consumption are influenced by broader dynamics, including public health interventions or socio-cultural behaviour, rather than just baseline consumption levels. These findings highlight the importance of considering both baseline consumption levels and external factors, such as policy and public health strategies, when interpreting trends in antibacterial drug use.

### Geographical distribution

The geographical distribution shows common patterns of antibacterial drug use across Europe, which can be divided into five geographic regions: Central, North, South, West and East. An evaluation of these trends highlights the stark contrasts and striking similarities in prescribing behaviour within and between these regions. Also in a global perspective, a distinction between regions with a high and a low consumption is possible (Figs. 2, 3 and 6).

Most countries in Northern and Central Europe have low levels of consumption and are projected to continue a downward trend in antibacterial drug use. Conversely, countries in Eastern and Southern Europe as well as in South America generally have higher levels of consumption, which are projected to increase over time. Western Europe and Asia shows a mixed pattern, with both low and high levels of consumption and different trends of increase and decrease. This variation can be further clarified by evaluating the mean defined daily dose (DID) within each region (Table 10). Around 2023, the lowest DID was reported in Central Europe (11.5), followed by Northern Europe (14.7), Western Europe (19.8), Eastern Europe (20.8), Southern Europe (24.4) and Latin America (27.4). Projections to 2040 maintain this ranking, with notable decreases in Central Europe (−5.2%, 10.9 DID) and Northern Europe (−9.7%, 13.3 DID). In contrast, consumption is expected to increase in Western Europe (+0.4%, 19.9 DID), Eastern Europe (+15.1%, 23.9 DID), Southern Europe (+16.2%, 28.4 DID) and Latin America (+45.0%, 43.2 DID). Central and Northern Europe will remain at relatively low levels, while Eastern and Southern Europe will remain high-consumption regions (ECDC 2024b).

**Table 10:**
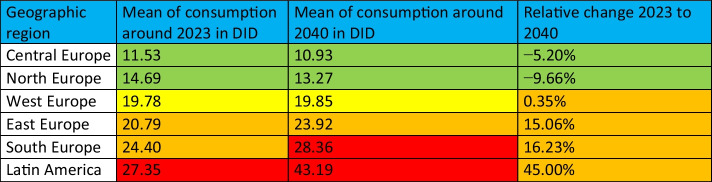
Consumption of antibacterial drugs by geographical region. The average for each region is calculated to allow comparison of consumption in 2023, projection for 2040 and relative change. Green-coloured is a low consumption level under 15 DID and a decreasing trend. Yellow-coloured is a moderate consumption level between 15- and 20 DID. Orange-coloured is a high consumption level over 20 DID and an increasing trend. Red-coloured is a very high consumption over 25 DID

Among the Northern European countries, Denmark shows a distinct behaviour compared to the other Nordic countries. This is characterised by the highest level of consumption within the group, an increasing trend in all sectors over the period covered with data and a projected increase in consumption until 2040. Several factors may contribute to this pattern. In the community sector, there has been an increase in the use of extended-spectrum agents and combination products such as pivmecillinam and amoxicillin-clavulanic acid, accompanied by a decrease in the use of beta-lactamase-sensitive penicillins. This trend is considered to be due to updated guidelines for the treatment of respiratory infections and a shift in physicians’ prescribing practices towards broader-spectrum antibacterial drugs (SSI 2015; DANMAP 2023). In the hospital setting, the growing use of combination penicillins is likely to be driven by the widespread adoption of piperacillin-tazobactam for empirical treatment of sepsis in most hospitals (SSI 2015).

This geographical variation in antibacterial consumption has been observed for many years (ECDC 2024b) and highlights differences in factors influencing prescribing behaviour. For example, overuse of antibacterial drugs in Latin America and Greece, particularly broad-spectrum agents such as amoxicillin-clavulanic acid, is associated with self-medication, insufficient knowledge among physicians, fear of treatment failure and patient expectations (Karakonstantis and Kalemaki 2019; Fabre et al. 2022). Similarly, self-medication through over-the-counter sales in pharmacies is widespread in Latin America and Southern Europe, contributing to high consumption in the Mediterranean region (Karakonstantis and Kalemaki 2019; Wolff 1993). In Cyprus, the lack of national treatment guidelines further exacerbates inappropriate prescribing practices, highlighting the role of limited training in antimicrobial stewardship and the influence of social and cultural factors (Spernovasilis and Tsioutis 2024).

Socio-economic and cultural determinants also shape these trends. Factors such as education, healthcare organisation, agriculture, religion and socioeconomic inequalities have a significant impact on antibacterial consumption and resistance development (Blommaert et al. 2014, Fabre et al. 2022). For example, Northern European countries with Protestant populations often have lower antibacterial consumption due to cultural scepticism and stricter health policies, in contrast to Catholic-majority Southern Europe, where higher use rates prevail (Harbarth and Monnet 2008). The prevalence of over-the-counter sales and self-medication is also higher in Eastern and Southern Europe, with countries such as Romania and Greece reporting non-prescription use rates of 20% and 16% respectively, while the lowest rates are reported for Sweden and Slovenia (European Commission 2017a, 2017b).

Despite these differences, the incidence of bacterial infections does not differ significantly between countries and cannot fully explain the substantial differences in antibacterial consumption (Harbarth and Monnet 2008). Instead, regional differences are mainly driven by prescribing practices, patient demand, cultural pressures and the regulatory environment. In Belgium, for example, a ‘no risk’ attitude among doctors has led to higher prescribing rates than in the Netherlands, where a more conservative approach is taken (Harbarth and Monnet 2008). Such variations underline the importance of targeted interventions to address the specific drivers of high consumption and to promote prudent use, particularly in high consumption regions such as Southern and Eastern Europe.

This apparent shift therefore represents a structural problem that is expected to worsen in the coming years. This challenge is compounded by the increase in bacterial resistance associated with high consumption (Karakonstantis and Kalemaki 2019; Bindel and Seifert 2024b), particularly in high consumption regions such as Latin America, Southern and Eastern Europe (ECDC 2024c, Fabre et al. 2022). Moreover, the consequences of inappropriate use are expected to spill over into neighbouring regions, influencing resistance rates and undermining the effectiveness of treatments.

### Progress towards the EU targets on AMC until 2030

In 2023, the European Union launched action to combat antimicrobial resistance through a ‘One Health’ approach (EU 2023; ECDC 2024a). Three main targets have been set for Member States to achieve by 2030, using 2019 as a baseline: to reduce total antimicrobial consumption (AMC) in humans by 20%, to ensure that at least 65% of antimicrobial consumption from agents classified in the 'Access' group of the WHO AWaRe classification and to reduce the incidence of bloodstream infections caused by three key resistant pathogens (*S. aureus*, *E. coli*, *K. pneumoniae*) (EU 2023).

In order to prove the potential to reach this target, all projected countries are analysed with their projections up to 2030. These projections are compared with the 2019 data, as this is the baseline year, and an assessment is made of the projected relative change between the projection and the LCL in order to assess whether Member States are likely to achieve this approach. For each country, the consumption of the total care sector is used, with some exceptions where only sufficient data were available for the community sector, which was used as a substitute (see the ‘Methods and materials’ section).

**Fig. 8:**
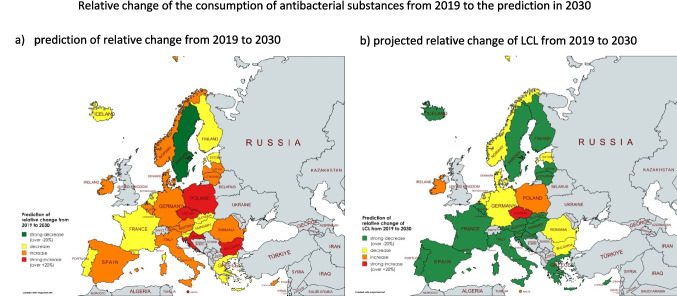
Assessment of the achievement of the target of a −20% reduction in antimicrobial consumption from 2019 to 2030 through the EU’s One Health approach. In (a) the relative change of the forecast is shown, while in (b) the relative change for the LCL is shown. Relative changes above −20% are coloured dark green, decreases between −20% and +−0% are coloured light green, increases between +−0% and +20% are coloured orange and large increases above +20% are coloured red. Countries for which no data were available are shown in grey. The map was produced using mapchart.net

Alarmingly, only one of the 29 countries analysed, Sweden, is projected to meet the 20% reduction target by 2030 (Table 11, Fig. 8). Sweden’s forecast shows a reduction of −27.2%, reaching 8.6 DID by 2030. In contrast, all other countries are projected to fall short, with 10 countries showing a reduction but not meeting the target, and 18 countries projected to experience an increase in antimicrobial consumption. These trends indicate not only a failure to meet the target, but also a worsening situation in many countries.

**Table 11:**
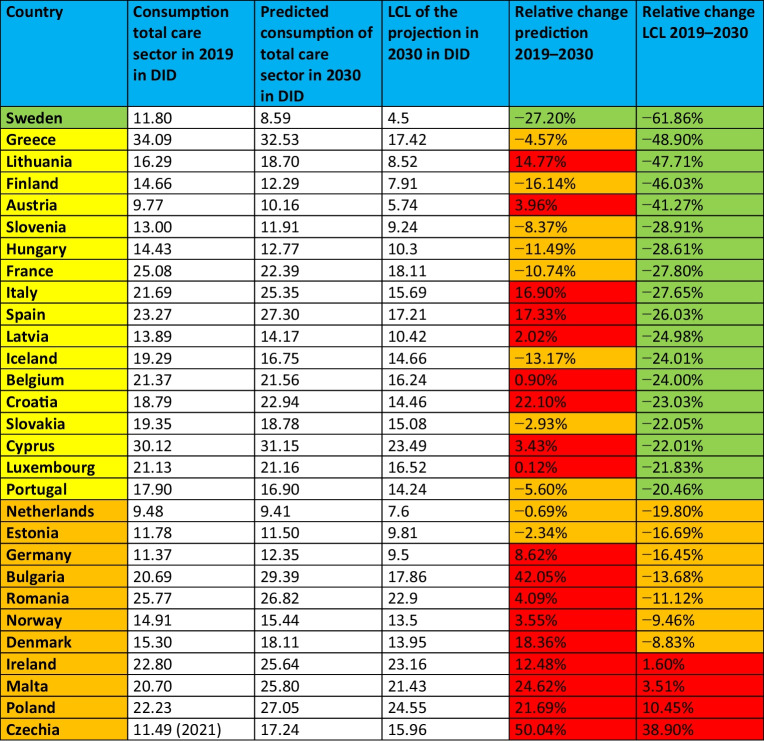
Assessment of the potential to achieve the One Health target of a −-20% reduction in antimicrobial consumption between 2019 and 2030. Green is the projected relative change where the reduction is successful, orange is where the reduction is insufficient, and red is where the consumption is increasing. Consumption in 2019, projection and LCL for 2023 and relative changes are shown. Countries that are projected to meet the target are shown in green. Countries that have the potential to meet the target according to their LCL but do not meet it in the projection are coloured yellow. Countries for which the forecast model excludes the possibility of reaching the target are coloured red. Countries are sorted ascending by the projected relative change

Considering the ‘best case’ scenario, represented by the LCL of the ARIMA(1,1,1) model, a few other countries might have the potential to reach the target. These include Austria, Belgium, Croatia, Cyprus, Finland, France, Greece, Hungary, Italy, Iceland, Lithuania, Luxembourg, Latvia, Portugal, Slovenia, Slovakia and Spain. However, for 11 countries the model does not predict any possibility of reaching the target. Of these, 7 countries, including Bulgaria, Denmark, Estonia, Germany, Malta, the Netherlands, Norway and Romania, show some potential to achieve partial reductions. Conversely, in others, such as Czechia, Ireland, Malta and Poland, an increase in antimicrobial consumption is still predicted.

In summary, the likelihood of achieving the EU’s One Health targets appears minimal if current trends continue. Developments from 2019 to 2023 has already shown poor progress, with no significant reductions observed in any country or across Europe (ECDC 2024a). If this trajectory continues, it is highly unlikely that the targets will be met, exacerbating the already critical problem of bacterial resistance. This underlines the urgent need for increased efforts and strategic interventions to reduce antimicrobial consumption and improve stewardship practices in all EU Member States.

### Comparison of the development of the community and hospital sector in European countries

**Fig. 9:**
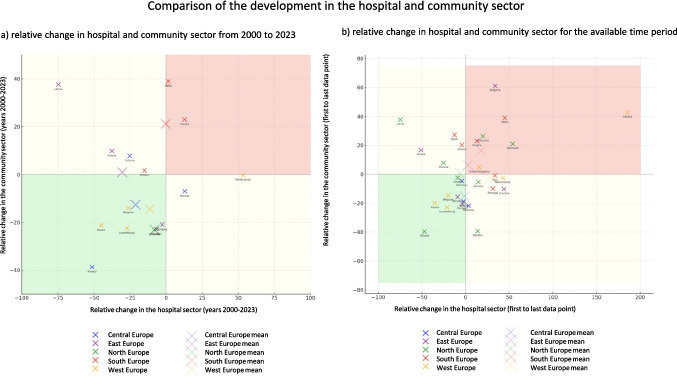
Comparison of developments for the two sectors of community and hospital care. The relative changes are shown (a) for the period 2000–2023, to allow comparison between countries, and (b) for the period covered by a single country, to allow country-specific assessment. For each region, the average development is shown. The graphs are divided into four sectors. Areas where both sectors show a decline are shown in green. Yellow is where one sector is increasing and the other is decreasing. Red is the area where both sectors are increasing

In addition to the total consumption of antibacterial drugs, many countries provide data for individual sectors. This makes it possible to analyse and assess the development of the community care and hospital sectors in countries where both sets of data are available (Table 3). This study covers 28 countries: Austria, Belgium, Bulgaria, Croatia, Czechia, Denmark, Estonia, Finland, France, Germany, Greece, Hungary, Ireland, Italy, Iceland, Lithuania, Luxembourg, Latvia, Malta, the Netherlands, Norway, Poland, Portugal, Slovenia, Slovakia, Spain, Sweden and the UK. Within each country, trends (either increasing or decreasing) are compared (similar vs. opposite) and consumption levels are analysed between countries within each sector (Fig. 9).

This comparison aims to identify problematic trends in sectors where additional efforts are needed to reduce consumption. A similar decreasing trend is positive and indicates that effective measures are in place, whereas a similar increasing trend indicates that interventions have failed. Divergent trends, where one sector is increasing while the other is decreasing, require an investigation of the underlying causes in order to effectively address the problematic sector.

A similar decreasing trend from the first to the last data point is observed in Finland, France, Luxembourg, Belgium, Iceland, Germany, Greece, Hungary and Austria. These countries appear to have implemented comprehensive measures in both sectors, resulting in successful reductions in consumption (ECDC 2024a). For example, Finland has reported sustained reductions due to robust antimicrobial stewardship programmes and public awareness campaigns (Hakanen et al. 2017).

Conversely, Croatia, the UK, Lithuania, Bulgaria, Malta, Denmark and Ireland show a similar upward trend from the first to the last data point. These developments are worrying, as they suggest that measures to reduce consumption in both sectors have failed. However, Denmark is a special case. Although it reported increases in both sectors, the base consumption in 2000 was exceptionally low, making the increases less alarming than in other countries, although still unfavourable.

An opposite trend, where one sector increases while the other decreases, is observed in 12 countries. A decrease in the community sector combined with an increase in the hospital sector is observed in Slovenia, Sweden, Norway, Portugal, Italy, Czechia and Malta. In Sweden, the relative decrease in the community sector outweighs the increase in the hospital sector, suggesting a net decrease in total consumption. In other countries, however, small decreases in the community sector are overshadowed by substantial increases in the hospital sector. Possible reasons for this include increasing hospital-acquired infections, limited diagnostic capacity in hospitals leading to the use of broader-spectrum agents, inadequate infection prevention measures in hospitals or the prophylactic use of antibacterial drugs (ECDC 2024d).

The opposite combination, where the hospital sector decreases and the community sector increases, is observed in Latvia, Poland, Estonia, Spain and Germany. This could be due to insufficient public health interventions targeting community prescribing, higher patient demand for antibacterial drugs or a lack of strict prescribing rules in outpatient care. In Spain and Germany, for example, irrational prescribing behaviour or cultural factors may contribute to persistent overprescribing in the community, despite efforts to reduce hospital use (Karakonstantis and Kalemaki 2019; Spernovasilis and Tsioutis 2024; Bindel and Seifert 2024b).

In summary, sectors within a country do not necessarily evolve in parallel. This divergence complicates the assessment of future trends, as the dynamics of antibacterial consumption depend on sector-specific policies, health infrastructure and societal behaviours. Effective interventions need to take these differences into account and ensure tailored strategies for each sector to achieve sustainable reductions in consumption.

## Limitations

This study is based on data extracted from the ECDC Antimicrobial Consumption Dashboard (ESAC-net 2024), which aggregates information provided by national authorities in each country. Differences in methodologies, data collection procedures and the inclusion of different medical prescription fields between countries were not explicitly described in a common methodological section. These differences may have led to breaks in time series and potential data bias. To address a specific bias, we manually defined a temporary outlier for the year 2020, as the COVID pandemic strongly affected most countries. Without this adjustment, the ARIMA model incorrectly interpreted the pandemic-induced fluctuations as the start of a new trend rather than a temporary disturbance. This adjustment was necessary to reflect reality, as consumption levels in 2023 exceeded pre-pandemic levels from 2019 onwards, showing no clear downward trend either for individual countries or for Europe as a whole (ECDC 2024a).

Data availability was an additional challenge, particularly for the total care sector. In some countries, such as Austria, Czechia, Germany, Poland, Romania and Spain, data were only available for the outpatient sector. In these cases, the community care sector was used as a substitute for the total care sector because of its dominant share in total consumption, as indicated by the ratio of DID in the comparison of hospital and community sectors. While this substitution allowed robust forecasting models for these countries, it slightly underestimated total consumption, as the hospital sector, which typically accounts for 1–3 DID, was excluded. In addition, the development of the hospital sector could not be taken into account.

The feasibility of predictive modelling depended heavily on the quality and completeness of the data for each country. In some cases, limited data quality resulted in poor fit metrics or the inability to construct a viable model, as was the case for the UK. In addition, not all countries provided data for the full period under analysis, limiting comparative assessments. For example, while trends from 2000 to 2023 were often analysed, countries that only started reporting data in 2008 could not be included in these comparisons. To mitigate this, analyses often took into account both the full period available for each country and the broader period 2000–2023 to allow meaningful international and country-specific comparisons.

While ARIMA models are widely regarded as effective for forecasting time series (Hyndman and Athanasopoulos 2021; Bindel and Seifert 2025a), they have inherent limitations. A key limitation is the assumption of linear relationships between time points, which may fail to capture non-linear patterns in the data (Bindel and Seifert 2025a, 2025b). In addition, ARIMA models do not take into account external factors that may influence outcomes but were not included in the analysis. The impact of such unaccounted variables remains uncertain and predictions are inherently constrained by the current state of knowledge. As a result, unanticipated future developments cannot be accurately predicted (Bindel and Seifert 2025a). Short-term projections are generally reliable for countries with well-fitting models. However, long-term projections to 2040 are subject to greater uncertainty and should be interpreted as indicative of trends rather than precise values.

This study followed strict criteria for statistical methods and model evaluation, as detailed in the ‘Methods and materials’ section. A single ARIMA models was selected as the most appropriate approach based on these predefined criteria. However, alternative statistical methods, modifications to the ARIMA model or changes in the criteria used to evaluate the fit metrics could lead to different conclusions, highlighting the influence of methodological choices on forecasting results.

## Conclusions and further perspectives

Analysis of antimicrobial consumption trends across Europe and OECD countries reveals significant regional disparities and highlights the complexity of addressing antibacterial resistance (AMR) through targeted interventions. Northern and Central Europe continue to have relatively low levels of consumption and are projected to decrease, reflecting effective antimicrobial stewardship and public health strategies. Conversely, Latin America, Southern and Eastern Europe show persistently high levels of consumption, often with projected increases, highlighting the structural challenges in these regions.

The shift in consumption trends between regions has far-reaching implications. The widening gap between low and high consumption regions threatens the achievement of the EU’s ‘One Health’ targets for 2030, which aim to reduce antimicrobial consumption by 20%, increase the proportion of antibacterial classified in the 'Access' group of the AWaRe framework with at least 65% and reduce the incidence of bloodstream infections caused by major resistant pathogens. Alarmingly, only one of the 29 countries analysed, Sweden, is on track to meet the reduction target, while the majority are projected to show either minimal decreases or even increases. This trend not only highlights the challenges of meeting EU targets, but also risks exacerbating AMR in high consumption regions, with potential spillover effects on neighbouring countries and the wider European healthcare landscape.

The persistence of high levels of consumption in regions such as Latin America Southern and Eastern Europe is due to various factors, including cultural practices, lack of guidance and restriction programmes and the prevalence of over-the-counter sales (Karakonstantis and Kalemaki 2019; Spernovasilis and Tsioutis 2024; Blommaert et al. 2014; Harbarth and Monnet 2008; European Commission 2017b; Fabre et al. 2022). These structural problems of high consumption are accompanied by increasing AMR (Bindel and Seifert 2024a; Karakonstantis and Kalemaki 2019), particularly in these high-consumption regions, where resistance rates are already high (ECDC 2024b). Such developments threaten the effectiveness of current treatment regimens and could lead to increased morbidity and mortality associated with resistant bacteria (ECDC 2024a).

The analysis also highlights the importance of addressing inequalities within countries, particularly between the community and hospital sectors. Divergent trends in these sectors complicate the assessment of national progress and highlight the need for targeted interventions. For example, rising consumption in the hospital sector in countries such as Italy and Portugal may be related to hospital-acquired infections and prophylactic use of antibacterials (ECDC 2024d), while rising consumption in the community sector in countries such as Spain and Germany reflect ongoing challenges in outpatient prescribing practices (Karakonstantis and Kalemaki 2019; Bindel and Seifert 2024b).

In summary, the current trends in antimicrobial consumption in Europe and OECD countries pose significant challenges to the achievement of EU targets and the reduction of AMR in general. The observed shift from favourable trends in low consumption regions to problematic trends in high consumption regions underlines the urgency of strengthening antimicrobial stewardship programmes, ensuring coordination of policies across sectors, and addressing the socio-cultural and structural determinants of consumption. Without intensified and coordinated efforts, the continued rise of AMR will undermine the effectiveness of treatments and put additional strain on healthcare systems and public health outcomes.

Further research is needed to refine these findings and to explore additional aspects of antibacterial consumption and resistance. Beyond total consumption, analyses should specifically address the other objectives of the ‘One Health’ approach, including the use of antibacterial classes classified under the AWaRe framework and trends in bacterial resistance. Both aspects require comprehensive assessment and long-term forecasting to align with strategic objectives. In addition, detailed assessments at country level are essential to identify targeted interventions to promote rational prescribing practices and achieve sustainable reductions in antibacterial consumption.

## Supplementary Information

Below is the link to the electronic supplementary material.

Supplementary file1 (DOCX 30668 KB)

## Data Availability

All source data for this work (or generated in this study) are available upon reasonable request.

## Abbreviations

ACF: Autocorrelation function
ARIMA: Auto Regressive Integrated Moving Average model (mathematical model for time series analysis)
AMC: Antimicrobial consumption
AMR: Antimicrobial resistance
AWaRe: Classification of antibacterial substances to support rational use and monitoring antibacterial consumption (divided into groups Access, Watch, Reserve) (WHO 2023)
DDD: Defined Daily Dose
DID: Defined Daily Dose prescriptions per 1000 inhabitants per day
ECDC: European Centre for Disease Prevention and Control (public health agency of the EU)
EU: European Union
LCL: Lower control limit
MRSA: Methicillin resistant *Staphylococcus aureus*
OECD: Organisation for economic cooperation and development
PACF: Partial autocorrelation function
SPSS: Statistical Product and Service Solutions (software for statistical analyses, IBM)
UCL: Upper control limit
WHO: World Health Organization
