# Supplementary material for: Most European countries will miss EU targets on antibacterial use by 2030: historical analysis of European and OECD countries, comparison of community and hospital sectors and forecast to 2040
Source: Naunyn Schmiedebergs Arch Pharmacol. 2025 Feb 17;398(8):10195–220. doi: 10.1007/s00210-025-03887-5 (PMC12350445; doi:10.1007/s00210-025-03887-5)

**Most European countries will miss EU targets on antibacterial use by 2030: historical analysis of European and OECD countries, comparison of community and hospital sectors and forecast to 2040**

**Lilly Josephine Bindel and Roland Seifert**

Supplemental Tables

***Table S1:*** *Forecast of antibacterial drug consumption from 2024 to 2040 for Austria, Belgium, Bulgaria, Croatia, Cyprus and Czechia. The predicted value, the UCL and the LCL are reported in DID.*

| **country** | **Austria** | | | **Belgium** | | | **Bulgaria** | | | **Croatia** | | | **Cyprus** | | | **Czechia** | | |
| --- | --- | --- | --- | --- | --- | --- | --- | --- | --- | --- | --- | --- | --- | --- | --- | --- | --- | --- |
| **para-meter** | **forecast** | UCL | LCL | **forecast** | UCL | LCL | **forecast** | UCL | LCL | **forecast** | UCL | LCL | **forecast** | UCL | LCL | **forecast** | UCL | LCL |
| **2022** | **.** | . | . | **.** | . | . | **.** | . | . | **.** | . | . | **.** | . | . | **.** | . | . |
| **2023** | **.** | . | . | **.** | . | . | **.** | . | . | **.** | . | . | **31.44** | 38.11 | 24.77 | **.** | . | . |
| **2024** | **9.83** | 11.72 | 7.94 | **21.35** | 23.41 | 19.29 | **26.80** | 30.95 | 22.64 | **21.52** | 24.24 | 18.79 | **30.65** | 38.06 | 23.23 | **15.63** | 17.09 | 14.17 |
| **2025** | **9.93** | 12.32 | 7.54 | **21.23** | 24.06 | 18.40 | **27.17** | 33.34 | 20.99 | **21.89** | 26.39 | 17.40 | **30.43** | 38.03 | 22.83 | **16.02** | 17.29 | 14.75 |
| **2026** | **10.08** | 13.06 | 7.11 | **21.67** | 25.17 | 18.17 | **27.66** | 35.13 | 20.18 | **22.09** | 27.51 | 16.68 | **30.45** | 38.11 | 22.80 | **16.31** | 17.59 | 15.04 |
| **2027** | **10.11** | 13.46 | 6.76 | **21.37** | 25.38 | 17.37 | **28.04** | 36.78 | 19.3 | **22.35** | 28.73 | 15.96 | **30.58** | 38.25 | 22.91 | **16.56** | 17.84 | 15.29 |
| **2028** | **10.16** | 13.92 | 6.40 | **21.68** | 26.19 | 17.18 | **28.52** | 38.24 | 18.81 | **22.53** | 29.66 | 15.41 | **30.76** | 38.43 | 23.08 | **16.8** | 18.07 | 15.52 |
| **2029** | **10.15** | 14.24 | 6.07 | **21.32** | 26.22 | 16.41 | **28.92** | 39.62 | 18.21 | **22.75** | 30.6 | 14.90 | **30.95** | 38.62 | 23.28 | **17.02** | 18.29 | 15.74 |
| **2030** | **10.16** | 14.57 | 5.74 | **21.56** | 26.88 | 16.24 | **29.39** | 40.91 | 17.86 | **22.94** | 31.42 | 14.46 | **31.15** | 38.82 | 23.49 | **17.24** | 18.52 | 15.96 |
| **2031** | **10.13** | 14.83 | 5.43 | **21.17** | 26.83 | 15.50 | **29.79** | 42.16 | 17.43 | **23.14** | 32.23 | 14.05 | **31.36** | 39.02 | 23.70 | **17.46** | 18.73 | 16.18 |
| **2032** | **10.10** | 15.09 | 5.12 | **21.38** | 27.41 | 15.36 | **30.25** | 43.34 | 17.16 | **23.33** | 32.98 | 13.68 | **31.57** | 39.22 | 23.91 | **17.68** | 18.95 | 16.40 |
| **2033** | **10.07** | 15.31 | 4.82 | **20.99** | 27.32 | 14.65 | **30.66** | 44.49 | 16.83 | **23.53** | 33.72 | 13.35 | **31.77** | 39.42 | 24.13 | **17.89** | 19.17 | 16.62 |
| **2034** | **10.03** | 15.53 | 4.53 | **21.18** | 27.84 | 14.52 | **31.12** | 45.61 | 16.63 | **23.72** | 34.41 | 13.04 | **31.98** | 39.62 | 24.34 | **18.11** | 19.39 | 16.84 |
| **2035** | **9.99** | 15.72 | 4.25 | **20.78** | 27.72 | 13.85 | **31.54** | 46.69 | 16.38 | **23.92** | 35.09 | 12.75 | **32.19** | 39.82 | 24.56 | **18.33** | 19.61 | 17.05 |
| **2036** | **9.94** | 15.91 | 3.98 | **20.96** | 28.20 | 13.73 | **31.99** | 47.75 | 16.22 | **24.12** | 35.75 | 12.48 | **32.40** | 40.02 | 24.77 | **18.55** | 19.83 | 17.27 |
| **2037** | **9.90** | 16.08 | 3.71 | **20.58** | 28.07 | 13.08 | **32.41** | 48.78 | 16.04 | **24.31** | 36.39 | 12.23 | **32.61** | 40.22 | 24.99 | **18.77** | 20.04 | 17.49 |
| **2038** | **9.85** | 16.25 | 3.45 | **20.75** | 28.51 | 12.98 | **32.85** | 49.79 | 15.91 | **24.51** | 37.02 | 11.99 | **32.81** | 40.42 | 25.20 | **18.98** | 20.26 | 17.71 |
| **2039** | **9.81** | 16.41 | 3.20 | **20.37** | 28.38 | 12.35 | **33.28** | 50.78 | 15.77 | **24.70** | 37.63 | 11.77 | **33.02** | 40.63 | 25.42 | **19.20** | 20.48 | 17.92 |
| **2040** | **9.76** | 16.57 | 2.95 | **20.52** | 28.79 | 12.26 | **33.72** | 51.76 | 15.68 | **24.90** | 38.23 | 11.57 | **33.23** | 40.83 | 25.63 | **19.42** | 20.70 | 18.14 |

***Table S2:*** *Forecast of antibacterial drug consumption from 2024 to 2040 for Denmark, Estonia, Finland, France, Germany and Greece. The predicted value, the UCL and the LCL are reported in DID.*

| **country** | **Denmark** | |  |  | **Estonia** |  |  | **Finland** |  |  | **France** |  |  | **Germany** |  |  | **Greece** |  |  |
| --- | --- | --- | --- | --- | --- | --- | --- | --- | --- | --- | --- | --- | --- | --- | --- | --- | --- | --- | --- |
| **para-meter** | **forecast** | UCL | | LCL | **forecast** | UCL | LCL | **forecast** | UCL | LCL | **forecast** | UCL | LCL | **forecast** | UCL | LCL | **forecast** | UCL | LCL |
| **2022** | **.** | . | | . | **.** | . | . | **.** | . | . | **.** | . | . | **.** | . | . | **.** | . | . |
| **2023** | **.** | . | | . | **.** | . | . | **.** | . | . | **.** | . | . | **.** | . | . | **.** | . | . |
| **2024** | **16.61** | 17.79 | | 15.42 | **11.75** | 13.43 | 10.07 | **13.16** | 14.99 | 11.34 | **23.98** | 26.37 | 21.59 | **11.37** | 13.05 | 9.68 | **29.14** | 35.74 | 22.53 |
| **2025** | **16.95** | 18.8 | | 15.10 | **11.65** | 13.35 | 9.96 | **12.95** | 15.29 | 10.60 | **23.78** | 26.91 | 20.65 | **11.73** | 13.62 | 9.84 | **29.73** | 38.84 | 20.63 |
| **2026** | **17.24** | 19.65 | | 14.82 | **11.62** | 13.32 | 9.93 | **13.01** | 15.95 | 10.07 | **23.53** | 27.09 | 19.96 | **11.93** | 14.05 | 9.80 | **30.32** | 41.19 | 19.44 |
| **2027** | **17.49** | 20.41 | | 14.57 | **11.60** | 13.29 | 9.90 | **12.75** | 16.05 | 9.44 | **23.25** | 27.10 | 19.39 | **12.08** | 14.41 | 9.76 | **30.89** | 43.15 | 18.63 |
| **2028** | **17.71** | 21.08 | | 14.34 | **11.56** | 13.26 | 9.87 | **12.7** | 16.43 | 8.97 | **22.96** | 27.00 | 18.91 | **12.20** | 14.72 | 9.69 | **31.45** | 44.83 | 18.07 |
| **2029** | **17.92** | 21.70 | | 14.13 | **11.53** | 13.22 | 9.84 | **12.42** | 16.46 | 8.38 | **22.67** | 26.85 | 18.49 | **12.29** | 14.98 | 9.60 | **32.00** | 46.31 | 17.68 |
| **2030** | **18.11** | 22.27 | | 13.95 | **11.50** | 13.19 | 9.81 | **12.29** | 16.68 | 7.91 | **22.39** | 26.67 | 18.11 | **12.35** | 15.21 | 9.50 | **32.53** | 47.64 | 17.42 |
| **2031** | **18.30** | 22.81 | | 13.79 | **11.47** | 13.16 | 9.77 | **12.00** | 16.66 | 7.34 | **22.11** | 26.46 | 17.76 | **12.41** | 15.41 | 9.40 | **33.06** | 48.85 | 17.27 |
| **2032** | **18.48** | 23.32 | | 13.64 | **11.43** | 13.13 | 9.74 | **11.83** | 16.79 | 6.87 | **21.84** | 26.24 | 17.44 | **12.44** | 15.60 | 9.29 | **33.58** | 49.96 | 17.20 |
| **2033** | **18.65** | 23.80 | | 13.50 | **11.4** | 13.09 | 9.71 | **11.53** | 16.74 | 6.33 | **21.57** | 26.01 | 17.14 | **12.48** | 15.77 | 9.18 | **34.09** | 50.99 | 17.20 |
| **2034** | **18.82** | 24.26 | | 13.38 | **11.37** | 13.06 | 9.68 | **11.33** | 16.80 | 5.86 | **21.31** | 25.78 | 16.85 | **12.50** | 15.93 | 9.07 | **34.60** | 51.94 | 17.25 |
| **2035** | **18.99** | 24.71 | | 13.28 | **11.33** | 13.03 | 9.64 | **11.03** | 16.73 | 5.34 | **21.05** | 25.54 | 16.57 | **12.52** | 16.08 | 8.96 | **35.09** | 52.84 | 17.35 |
| **2036** | **19.16** | 25.14 | | 13.18 | **11.30** | 12.99 | 9.61 | **10.8** | 16.74 | 4.87 | **20.80** | 25.30 | 16.30 | **12.53** | 16.22 | 8.85 | **35.58** | 53.67 | 17.49 |
| **2037** | **19.32** | 25.56 | | 13.09 | **11.27** | 12.96 | 9.58 | **10.51** | 16.66 | 4.36 | **20.55** | 25.06 | 16.04 | **12.55** | 16.35 | 8.74 | **36.07** | 54.47 | 17.67 |
| **2038** | **19.49** | 25.97 | | 13.01 | **11.24** | 12.93 | 9.55 | **10.27** | 16.63 | 3.90 | **20.30** | 24.82 | 15.78 | **12.56** | 16.48 | 8.63 | **36.55** | 55.22 | 17.87 |
| **2039** | **19.65** | 26.36 | | 12.94 | **11.20** | 12.89 | 9.51 | **9.97** | 16.54 | 3.40 | **20.05** | 24.57 | 15.53 | **12.57** | 16.60 | 8.53 | **37.02** | 55.94 | 18.10 |
| **2040** | **19.81** | 26.75 | | 12.88 | **11.17** | 12.86 | 9.48 | **9.72** | 16.49 | 2.95 | **19.81** | 24.33 | 15.28 | **12.58** | 16.72 | 8.43 | **37.49** | 56.62 | 18.35 |

***Table S3:*** *Forecast of antibacterial drug consumption from 2024 to 2040 for Hungary, Ireland, Italy, Iceland, Lithuania and Luxembourg. The predicted value, the UCL and the LCL are reported in DID.*

| **country** | **Hungary** | | | **Ireland** | | | **Italy** | | | **Iceland** | | | **Lithuania** | | | **Luxembourg** | | |
| --- | --- | --- | --- | --- | --- | --- | --- | --- | --- | --- | --- | --- | --- | --- | --- | --- | --- | --- |
| **parameter** | **forecast** | UCL | LCL | **forecast** | UCL | LCL | **forecast** | UCL | LCL | **forecast** | UCL | LCL | **forecast** | UCL | LCL | **forecast** | UCL | LCL |
| **2022** | **.** | . | . | **.** | . | . | **.** | . | . | **.** | . | . | **.** | . | . | **.** | . | . |
| **2023** | **.** | . | . | **.** | . | . | **.** | . | . | **.** | . | . | **.** | . | . | **.** | . | . |
| **2024** | **13.84** | 15.95 | 11.73 | **23.27** | 25.73 | 20.80 | **24.01** | 28.03 | 19.98 | **18.25** | 20.30 | 16.21 | **19.20** | 23.80 | 14.61 | **20.78** | 23.08 | 18.48 |
| **2025** | **13.59** | 15.96 | 11.22 | **23.83** | 26.31 | 21.35 | **24.14** | 29.35 | 18.93 | **18.03** | 20.10 | 15.97 | **19.04** | 24.69 | 13.39 | **21.03** | 23.63 | 18.42 |
| **2026** | **13.39** | 15.83 | 10.95 | **24.30** | 26.78 | 21.81 | **24.66** | 31.12 | 18.19 | **17.80** | 19.89 | 15.72 | **19.11** | 26.03 | 12.19 | **21.22** | 24.49 | 17.95 |
| **2027** | **13.22** | 15.68 | 10.76 | **24.69** | 27.17 | 22.20 | **24.76** | 32.09 | 17.43 | **17.56** | 19.65 | 15.47 | **18.98** | 26.79 | 11.17 | **21.27** | 24.85 | 17.69 |
| **2028** | **13.07** | 15.53 | 10.60 | **25.03** | 27.51 | 22.55 | **25.07** | 33.28 | 16.85 | **17.30** | 19.39 | 15.21 | **18.92** | 27.61 | 10.23 | **21.29** | 25.30 | 17.27 |
| **2029** | **12.92** | 15.39 | 10.45 | **25.35** | 27.83 | 22.86 | **25.16** | 34.10 | 16.21 | **17.03** | 19.12 | 14.94 | **18.80** | 28.25 | 9.35 | **21.23** | 25.54 | 16.92 |
| **2030** | **12.77** | 15.24 | 10.30 | **25.64** | 28.12 | 23.16 | **25.35** | 35.02 | 15.69 | **16.75** | 18.84 | 14.66 | **18.70** | 28.87 | 8.52 | **21.16** | 25.81 | 16.52 |
| **2031** | **12.62** | 15.09 | 10.15 | **25.93** | 28.41 | 23.45 | **25.44** | 35.74 | 15.13 | **16.47** | 18.56 | 14.38 | **18.58** | 29.42 | 7.74 | **21.06** | 25.98 | 16.14 |
| **2032** | **12.47** | 14.94 | 10.01 | **26.21** | 28.69 | 23.73 | **25.57** | 36.50 | 14.65 | **16.19** | 18.28 | 14.10 | **18.46** | 29.93 | 6.99 | **20.94** | 26.15 | 15.74 |
| **2033** | **12.33** | 14.80 | 9.86 | **26.48** | 28.96 | 24.00 | **25.65** | 37.15 | 14.15 | **15.91** | 18.00 | 13.81 | **18.34** | 30.41 | 6.28 | **20.81** | 26.27 | 15.35 |
| **2034** | **12.18** | 14.65 | 9.72 | **26.75** | 29.23 | 24.27 | **25.76** | 37.82 | 13.71 | **15.62** | 17.72 | 13.53 | **18.23** | 30.86 | 5.59 | **20.68** | 26.39 | 14.96 |
| **2035** | **12.04** | 14.50 | 9.57 | **27.02** | 29.50 | 24.54 | **25.84** | 38.42 | 13.26 | **15.34** | 17.43 | 13.24 | **18.11** | 31.29 | 4.93 | **20.53** | 26.49 | 14.58 |
| **2036** | **11.89** | 14.36 | 9.43 | **27.29** | 29.77 | 24.81 | **25.93** | 39.02 | 12.84 | **15.05** | 17.15 | 12.96 | **17.99** | 31.69 | 4.29 | **20.39** | 26.57 | 14.21 |
| **2037** | **11.75** | 14.21 | 9.28 | **27.56** | 30.03 | 25.08 | **26.01** | 39.59 | 12.43 | **14.77** | 16.86 | 12.67 | **17.87** | 32.08 | 3.66 | **20.24** | 26.64 | 13.83 |
| **2038** | **11.60** | 14.06 | 9.14 | **27.82** | 30.30 | 25.35 | **26.09** | 40.14 | 12.05 | **14.48** | 16.58 | 12.38 | **17.75** | 32.44 | 3.06 | **20.09** | 26.71 | 13.47 |
| **2039** | **11.46** | 13.92 | 8.99 | **28.09** | 30.57 | 25.61 | **26.17** | 40.68 | 11.67 | **14.19** | 16.29 | 12.10 | **17.63** | 32.80 | 2.47 | **19.93** | 26.76 | 13.11 |
| **2040** | **11.31** | 13.77 | 8.85 | **28.36** | 30.83 | 25.88 | **26.25** | 41.20 | 11.30 | **13.91** | 16.01 | 11.81 | **17.51** | 33.13 | 1.89 | **19.78** | 26.81 | 12.75 |

***Table S4:*** *Forecast of antibacterial drug consumption from 2024 to 2040 for Latvia, Malta, the Netherlands, Norway, Poland and Portugal. The predicted value, the UCL and the LCL are reported in DID.*

| **country** | **Latvia** | | | **Malta** | | | **Netherlands** | | | **Norway** | | | **Poland** | | | **Portugal** | | |
| --- | --- | --- | --- | --- | --- | --- | --- | --- | --- | --- | --- | --- | --- | --- | --- | --- | --- | --- |
| **para-meter** | **forecast** | UCL | LCL | **forecast** | UCL | LCL | **forecast** | UCL | LCL | **forecast** | UCL | LCL | **forecast** | UCL | LCL | **forecast** | UCL | LCL |
| **2022** | **.** | . | . | **.** | . | . | **.** | . | . | **.** | . | . | **.** | . | . | **.** | . | . |
| **2023** | **.** | . | . | **.** | . | . | **.** | . | . | **.** | . | . | **.** | . | . | **.** | . | . |
| **2024** | **14.70** | 17.30 | 12.10 | **23.46** | 27.74 | 19.17 | **9.58** | 10.23 | 8.93 | **15.54** | 16.90 | 14.17 | **23.98** | 26.36 | 21.59 | **17.61** | 19.89 | 15.33 |
| **2025** | **14.55** | 17.71 | 11.39 | **24.07** | 28.43 | 19.72 | **9.59** | 10.55 | 8.63 | **15.56** | 17.23 | 13.89 | **24.35** | 26.83 | 21.88 | **17.39** | 19.92 | 14.86 |
| **2026** | **14.43** | 17.88 | 10.99 | **24.48** | 28.86 | 20.11 | **9.57** | 10.74 | 8.40 | **15.55** | 17.36 | 13.75 | **25.15** | 27.64 | 22.65 | **17.25** | 19.87 | 14.63 |
| **2027** | **14.34** | 17.93 | 10.75 | **24.85** | 29.22 | 20.48 | **9.54** | 10.90 | 8.18 | **15.54** | 17.41 | 13.67 | **25.65** | 28.15 | 23.15 | **17.15** | 19.79 | 14.50 |
| **2028** | **14.27** | 17.94 | 10.59 | **25.18** | 29.55 | 20.81 | **9.50** | 11.03 | 7.98 | **15.51** | 17.42 | 13.60 | **26.16** | 28.66 | 23.67 | **17.06** | 19.71 | 14.40 |
| **2029** | **14.21** | 17.93 | 10.49 | **25.49** | 29.86 | 21.13 | **9.46** | 11.13 | 7.79 | **15.48** | 17.41 | 13.55 | **26.61** | 29.11 | 24.12 | **16.98** | 19.64 | 14.32 |
| **2030** | **14.17** | 17.92 | 10.42 | **25.80** | 30.17 | 21.43 | **9.41** | 11.21 | 7.60 | **15.44** | 17.38 | 13.50 | **27.05** | 29.55 | 24.55 | **16.90** | 19.56 | 14.24 |
| **2031** | **14.14** | 17.91 | 10.37 | **26.10** | 30.47 | 21.74 | **9.35** | 11.28 | 7.42 | **15.40** | 17.34 | 13.46 | **27.47** | 29.97 | 24.97 | **16.82** | 19.48 | 14.16 |
| **2032** | **14.12** | 17.89 | 10.34 | **26.40** | 30.77 | 22.04 | **9.29** | 11.34 | 7.24 | **15.36** | 17.30 | 13.41 | **27.88** | 30.37 | 25.38 | **16.74** | 19.40 | 14.08 |
| **2033** | **14.10** | 17.88 | 10.31 | **26.70** | 31.06 | 22.34 | **9.23** | 11.39 | 7.07 | **15.31** | 17.26 | 13.36 | **28.28** | 30.78 | 25.78 | **16.66** | 19.32 | 14.00 |
| **2034** | **14.08** | 17.88 | 10.29 | **27.00** | 31.36 | 22.64 | **9.17** | 11.44 | 6.90 | **15.26** | 17.21 | 13.31 | **28.67** | 31.17 | 26.18 | **16.58** | 19.24 | 13.92 |
| **2035** | **14.07** | 17.87 | 10.28 | **27.30** | 31.66 | 22.94 | **9.10** | 11.47 | 6.73 | **15.21** | 17.16 | 13.27 | **29.07** | 31.57 | 26.57 | **16.50** | 19.16 | 13.84 |
| **2036** | **14.07** | 17.87 | 10.27 | **27.60** | 31.96 | 23.24 | **9.04** | 11.51 | 6.57 | **15.17** | 17.11 | 13.22 | **29.46** | 31.96 | 26.96 | **16.42** | 19.08 | 13.76 |
| **2037** | **14.06** | 17.86 | 10.26 | **27.90** | 32.25 | 23.54 | **8.97** | 11.53 | 6.41 | **15.12** | 17.06 | 13.17 | **29.85** | 32.35 | 27.36 | **16.34** | 19.00 | 13.68 |
| **2038** | **14.05** | 17.86 | 10.25 | **28.19** | 32.55 | 23.84 | **8.91** | 11.56 | 6.26 | **15.07** | 17.01 | 13.12 | **30.24** | 32.74 | 27.75 | **16.26** | 18.92 | 13.59 |
| **2039** | **14.05** | 17.86 | 10.24 | **28.49** | 32.85 | 24.14 | **8.84** | 11.58 | 6.10 | **15.02** | 16.96 | 13.08 | **30.64** | 33.13 | 28.14 | **16.18** | 18.84 | 13.51 |
| **2040** | **14.00** | 17.86 | 10.24 | **28.79** | 33.14 | 24.44 | **8.77** | 11.60 | 5.95 | **14.97** | 16.91 | 13.03 | **31.03** | 33.52 | 28.53 | **16.10** | 18.76 | 13.43 |

***Table S5:*** *Forecast of antibacterial drug consumption from 2024 to 2040 for Romania, Slovenia, Slovakia, Spain, Sweden and Portugal. The predicted value, the UCL and the LCL are reported in DID.*

| **country** | **Romania** | | | **Slovenia** | | | **Slovakia** | | | **Spain** | | | **Sweden** | | | **Portugal** | | |
| --- | --- | --- | --- | --- | --- | --- | --- | --- | --- | --- | --- | --- | --- | --- | --- | --- | --- | --- |
| **para-meter** | **forecast** | UCL | LCL | **forecast** | UCL | LCL | **forecast** | UCL | LCL | **forecast** | UCL | LCL | **forecast** | UCL | LCL | **forecast** | UCL | LCL |
| **2022** | **.** | . | . | **.** | . | . | **.** | . | . | **.** | . | . | **9.86** | 10.80 | 8.93 | **.** | . | . |
| **2023** | **.** | . | . | **.** | . | . | **.** | . | . | **.** | . | . | **9.70** | 11.34 | 8.06 | **.** | . | . |
| **2024** | **26.93** | 30.35 | 23.50 | **13.14** | 14.91 | 11.37 | **19.72** | 23.55 | 15.89 | **23.85** | 27.84 | 19.86 | **9.54** | 11.70 | 7.38 | **17.61** | 19.89 | 15.33 |
| **2025** | **26.77** | 30.57 | 22.97 | **12.90** | 15.10 | 10.69 | **19.66** | 23.36 | 15.95 | **24.31** | 29.70 | 18.92 | **9.38** | 11.97 | 6.80 | **17.39** | 19.92 | 14.86 |
| **2026** | **26.72** | 30.61 | 22.83 | **12.67** | 15.09 | 10.25 | **19.52** | 23.22 | 15.81 | **25.29** | 31.96 | 18.62 | **9.22** | 12.17 | 6.28 | **17.25** | 19.87 | 14.63 |
| **2027** | **26.72** | 30.64 | 22.80 | **12.46** | 15.00 | 9.92 | **19.35** | 23.05 | 15.64 | **25.64** | 33.26 | 18.03 | **9.07** | 12.33 | 5.80 | **17.15** | 19.79 | 14.50 |
| **2028** | **26.75** | 30.67 | 22.82 | **12.26** | 14.87 | 9.66 | **19.16** | 22.87 | 15.46 | **26.39** | 34.94 | 17.84 | **8.91** | 12.47 | 5.34 | **17.06** | 19.71 | 14.40 |
| **2029** | **26.78** | 30.71 | 22.86 | **12.08** | 14.73 | 9.43 | **18.97** | 22.68 | 15.27 | **26.70** | 36.02 | 17.38 | **8.75** | 12.58 | 4.91 | **16.98** | 19.64 | 14.32 |
| **2030** | **26.82** | 30.74 | 22.90 | **11.91** | 14.58 | 9.24 | **18.78** | 22.49 | 15.08 | **27.30** | 37.39 | 17.21 | **8.59** | 12.68 | 4.50 | **16.90** | 19.56 | 14.24 |
| **2031** | **26.86** | 30.77 | 22.94 | **11.75** | 14.43 | 9.06 | **18.59** | 22.29 | 14.88 | **27.58** | 38.33 | 16.82 | **8.43** | 12.76 | 4.11 | **16.82** | 19.48 | 14.16 |
| **2032** | **26.90** | 30.81 | 22.98 | **11.59** | 14.29 | 8.90 | **18.39** | 22.10 | 14.69 | **28.08** | 39.50 | 16.66 | **8.27** | 12.82 | 3.72 | **16.74** | 19.40 | 14.08 |
| **2033** | **26.93** | 30.84 | 23.03 | **11.44** | 14.14 | 8.74 | **18.20** | 21.91 | 14.49 | **28.36** | 40.38 | 16.33 | **8.11** | 12.88 | 3.35 | **16.66** | 19.32 | 14.00 |
| **2034** | **26.97** | 30.88 | 23.07 | **11.30** | 14.00 | 8.59 | **18.00** | 21.71 | 14.30 | **28.79** | 41.41 | 16.18 | **7.95** | 12.93 | 2.98 | **16.58** | 19.24 | 13.92 |
| **2035** | **27.01** | 30.91 | 23.12 | **11.15** | 13.86 | 8.45 | **17.81** | 21.52 | 14.10 | **29.07** | 42.23 | 15.90 | **7.80** | 12.97 | 2.63 | **16.50** | 19.16 | 13.84 |
| **2036** | **27.05** | 30.95 | 23.16 | **11.01** | 13.72 | 8.30 | **17.61** | 21.32 | 13.91 | **29.46** | 43.17 | 15.76 | **7.64** | 13.00 | 2.28 | **16.42** | 19.08 | 13.76 |
| **2037** | **27.09** | 30.98 | 23.21 | **10.87** | 13.58 | 8.16 | **17.42** | 21.13 | 13.71 | **29.74** | 43.96 | 15.53 | **7.48** | 13.02 | 1.93 | **16.34** | 19.00 | 13.68 |
| **2038** | **27.13** | 31.02 | 23.25 | **10.73** | 13.44 | 8.03 | **17.22** | 20.93 | 13.52 | **30.11** | 44.83 | 15.39 | **7.32** | 13.04 | 1.60 | **16.26** | 18.92 | 13.59 |
| **2039** | **27.17** | 31.05 | 23.30 | **10.60** | 13.30 | 7.89 | **17.03** | 20.74 | 13.32 | **30.39** | 45.59 | 15.20 | **7.16** | 13.06 | 1.27 | **16.18** | 18.84 | 13.51 |
| **2040** | **27.21** | 31.09 | 23.34 | **10.46** | 13.16 | 7.76 | **16.84** | 20.54 | 13.13 | **30.74** | 46.40 | 15.08 | **7.00** | 13.06 | 0.94 | **16.10** | 18.76 | 13.43 |

***Table S6:*** *Forecast of antibacterial drug consumption from 2024 to 2040 for the non-European OECD countries Australia, Canada, Chile, Costa Rica, Israel, Japan and Korea. The predicted value, the UCL and the LCL are reported in DID.*

| **country** | **Australia** | | | **Canada** | | | **Chile** | | | **Costa Rica** | | | **Israel** | | | **Japan** | | | **Korea** | | |
| --- | --- | --- | --- | --- | --- | --- | --- | --- | --- | --- | --- | --- | --- | --- | --- | --- | --- | --- | --- | --- | --- |
| **parameter** | **Forecast** | UCL | LCL | **Forecast** | UCL | LCL | **Forecast** | UCL | LCL | **Forecast** | UCL | LCL | **Forecast** | UCL | LCL | **Forecast** | UCL | LCL | **Forecast** | UCL | LCL |
| **2021** | **.** | . | . | **.** | . | . | **.** | . | . | **.** | . | . | **.** | . | . | **10.88** | 12.21 | 9.54 | **.** | . | . |
| **2022** | **.** | . | . | **.** | . | . | **.** | . | . | **.** | . | . | **.** | . | . | **11.27** | 14.15 | 8.39 | **.** | . | . |
| **2023** | **16.62** | 18.59 | 14.65 | **.** | . | . | **.** | . | . | **.** | . | . | **.** | . | . | **11.58** | 15.45 | 7.72 | **23.14** | 26.59 | 19.69 |
| **2024** | **16.95** | 19.53 | 14.37 | **14.59** | 16.27 | 12.91 | **36.75** | 46.03 | 27.48 | **18.51** | 23.25 | 13.78 | **15.62** | 21.12 | 10.13 | **11.83** | 16.49 | 7.18 | **28.02** | 31.64 | 24.39 |
| **2025** | **17.21** | 20.16 | 14.26 | **14.87** | 19.05 | 10.7 | **38.47** | 50.45 | 26.49 | **19.5** | 24.94 | 14.06 | **15.13** | 21.08 | 9.18 | **12.03** | 17.36 | 6.71 | **25.16** | 30.1 | 20.22 |
| **2026** | **17.42** | 20.61 | 14.23 | **14.95** | 21.12 | 8.79 | **40.23** | 53.73 | 26.73 | **19.76** | 26.07 | 13.45 | **14.55** | 20.6 | 8.51 | **12.19** | 18.12 | 6.27 | **29.33** | 34.42 | 24.24 |
| **2027** | **17.59** | 20.94 | 14.23 | **14.97** | 22.76 | 7.18 | **42.03** | 56.47 | 27.59 | **20.05** | 27.07 | 13.03 | **13.92** | 19.98 | 7.86 | **12.32** | 18.79 | 5.85 | **26.43** | 32.48 | 20.37 |
| **2028** | **17.72** | 21.19 | 14.25 | **14.97** | 24.14 | 5.79 | **43.86** | 58.9 | 28.82 | **20.21** | 27.88 | 12.54 | **13.25** | 19.31 | 7.19 | **12.42** | 19.39 | 5.45 | **30.11** | 36.3 | 23.93 |
| **2029** | **17.82** | 21.37 | 14.27 | **14.95** | 25.35 | 4.56 | **45.71** | 61.15 | 30.28 | **20.31** | 28.58 | 12.04 | **12.56** | 18.62 | 6.51 | **12.5** | 19.94 | 5.06 | **27.29** | 34.26 | 20.32 |
| **2030** | **17.9** | 21.52 | 14.29 | **14.94** | 26.42 | 3.46 | **47.58** | 63.28 | 31.88 | **20.36** | 29.19 | 11.53 | **11.86** | 17.91 | 5.82 | **12.56** | 20.44 | 4.68 | **30.61** | 37.71 | 23.51 |
| **2031** | **17.97** | 21.63 | 14.31 | **14.92** | 27.41 | 2.44 | **49.47** | 65.35 | 33.59 | **20.37** | 29.72 | 11.01 | **11.15** | 17.19 | 5.12 | **12.61** | 20.91 | 4.31 | **27.93** | 35.7 | 20.17 |
| **2032** | **18.02** | 21.71 | 14.33 | **14.91** | 28.32 | 1.5 | **51.36** | 67.36 | 35.37 | **20.35** | 30.2 | 10.49 | **10.44** | 16.46 | 4.41 | **12.65** | 21.35 | 3.96 | **30.97** | 38.86 | 23.08 |
| **2033** | **18.06** | 21.78 | 14.34 | **14.89** | 29.17 | 0.62 | **53.27** | 69.34 | 37.2 | **20.3** | 30.63 | 9.97 | **9.72** | 15.74 | 3.71 | **12.68** | 21.76 | 3.61 | **28.46** | 36.92 | 19.99 |
| **2034** | **18.1** | 21.83 | 14.36 | **14.88** | 29.97 | -0.22 | **55.18** | 71.3 | 39.06 | **20.24** | 31.02 | 9.46 | **9** | 15.01 | 3 | **12.71** | 22.15 | 3.26 | **31.25** | 39.84 | 22.65 |
| **2035** | **18.12** | 21.87 | 14.37 | **14.86** | 30.73 | -1.01 | **57.09** | 73.24 | 40.95 | **20.16** | 31.38 | 8.95 | **8.28** | 14.28 | 2.29 | **12.72** | 22.52 | 2.93 | **28.91** | 38.02 | 19.8 |
| **2036** | **18.14** | 21.91 | 14.38 | **14.85** | 31.46 | -1.76 | **59.01** | 75.17 | 42.85 | **20.07** | 31.71 | 8.44 | **7.57** | 13.55 | 1.58 | **12.74** | 22.87 | 2.61 | **31.49** | 40.73 | 22.25 |
| **2037** | **18.16** | 21.93 | 14.39 | **14.83** | 32.15 | -2.49 | **60.93** | 77.09 | 44.77 | **19.98** | 32.02 | 7.94 | **6.85** | 12.83 | 0.87 | **12.75** | 23.21 | 2.29 | **29.32** | 39.02 | 19.62 |
| **2038** | **18.18** | 21.95 | 14.4 | **14.82** | 32.82 | -3.18 | **62.86** | 79.01 | 46.7 | **19.88** | 32.31 | 7.45 | **6.13** | 12.1 | 0.15 | **12.75** | 23.53 | 1.98 | **31.72** | 41.55 | 21.89 |
| **2039** | **18.19** | 21.97 | 14.41 | **14.8** | 33.46 | -3.85 | **64.78** | 80.92 | 48.64 | **19.78** | 32.58 | 6.97 | **5.41** | 11.37 | -0.56 | **12.76** | 23.85 | 1.67 | **29.71** | 39.96 | 19.46 |
| **2040** | **18.2** | 21.98 | 14.42 | **14.79** | 34.07 | -4.5 | **66.71** | 82.84 | 50.58 | **19.67** | 32.84 | 6.49 | **4.69** | 10.64 | -1.27 | **12.76** | 24.15 | 1.38 | **31.94** | 42.32 | 21.56 |

Supplemental Figures

***Fig. S1:*** *Original time series of ATC class J01 for Austria. In a) the sequence chart of consumption in DID is shown, while b) displays the ACF plot of the autocorrelation. The non-stationarity can be seen in the visible trend in both the sequence chart and the ACF plot.*


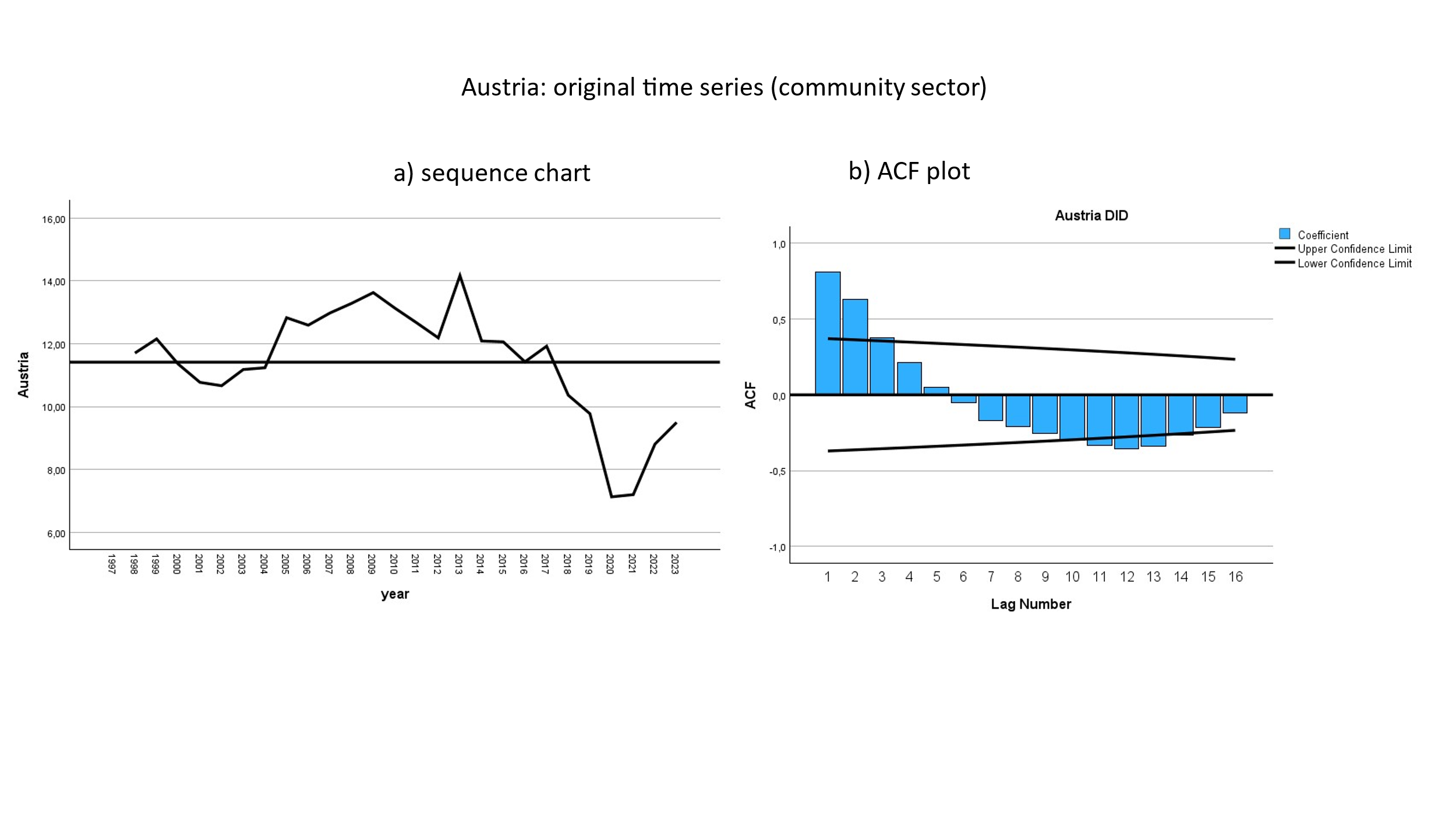


***Fig. S2:*** *Original time series of ATC class J01 for Belgium. In a) the sequence chart of consumption in DID is shown, while b) displays the ACF plot of the autocorrelation. The non-stationarity can be seen in the visible trend in both the sequence chart and the ACF plot.*


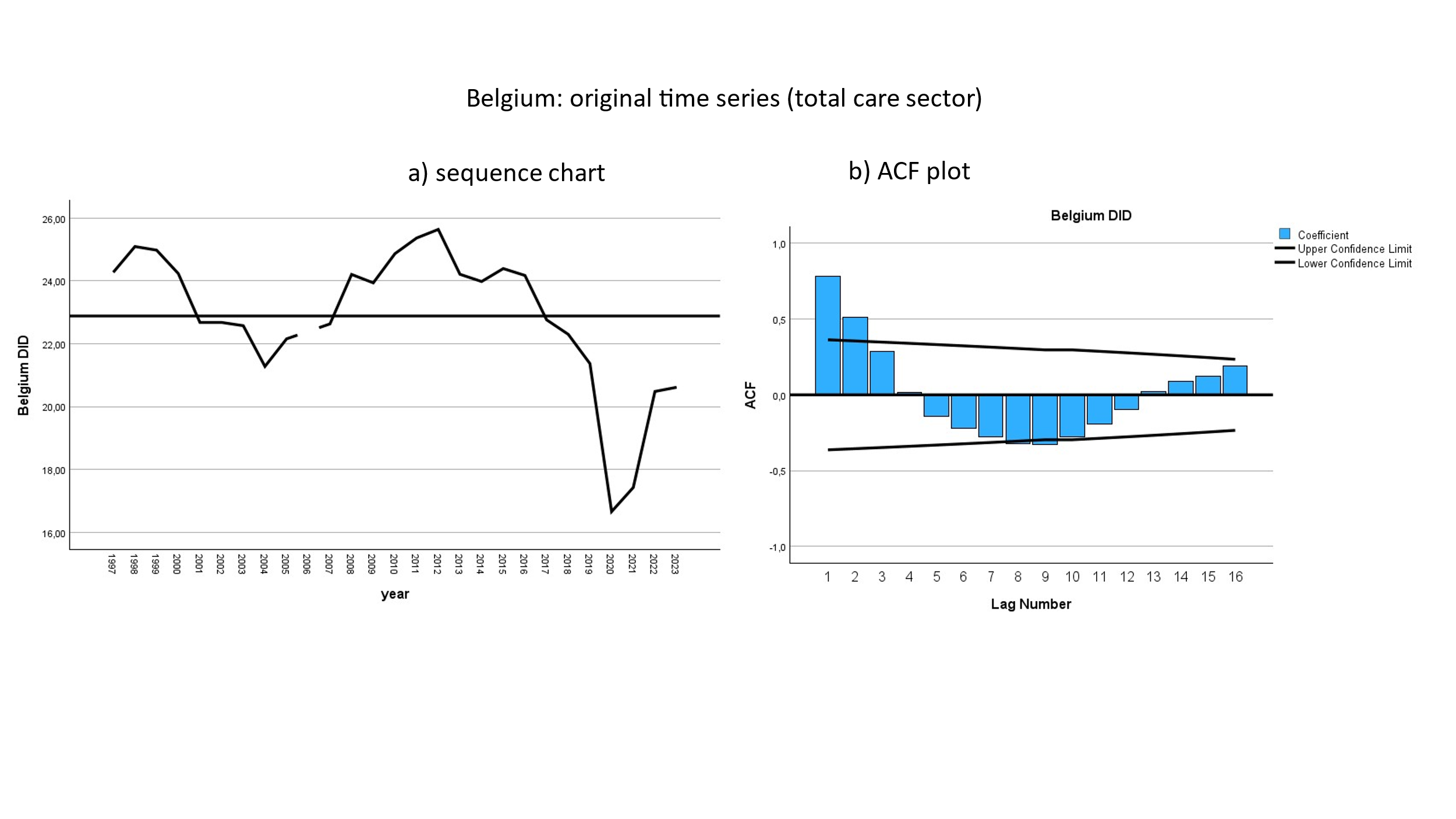


***Fig. S3:*** *Original time series of ATC class J01 for Bulgaria. In a) the sequence chart of consumption in DID is shown, while b) displays the ACF plot of the autocorrelation. The non-stationarity can be seen in the visible trend in both the sequence chart and the ACF plot.*


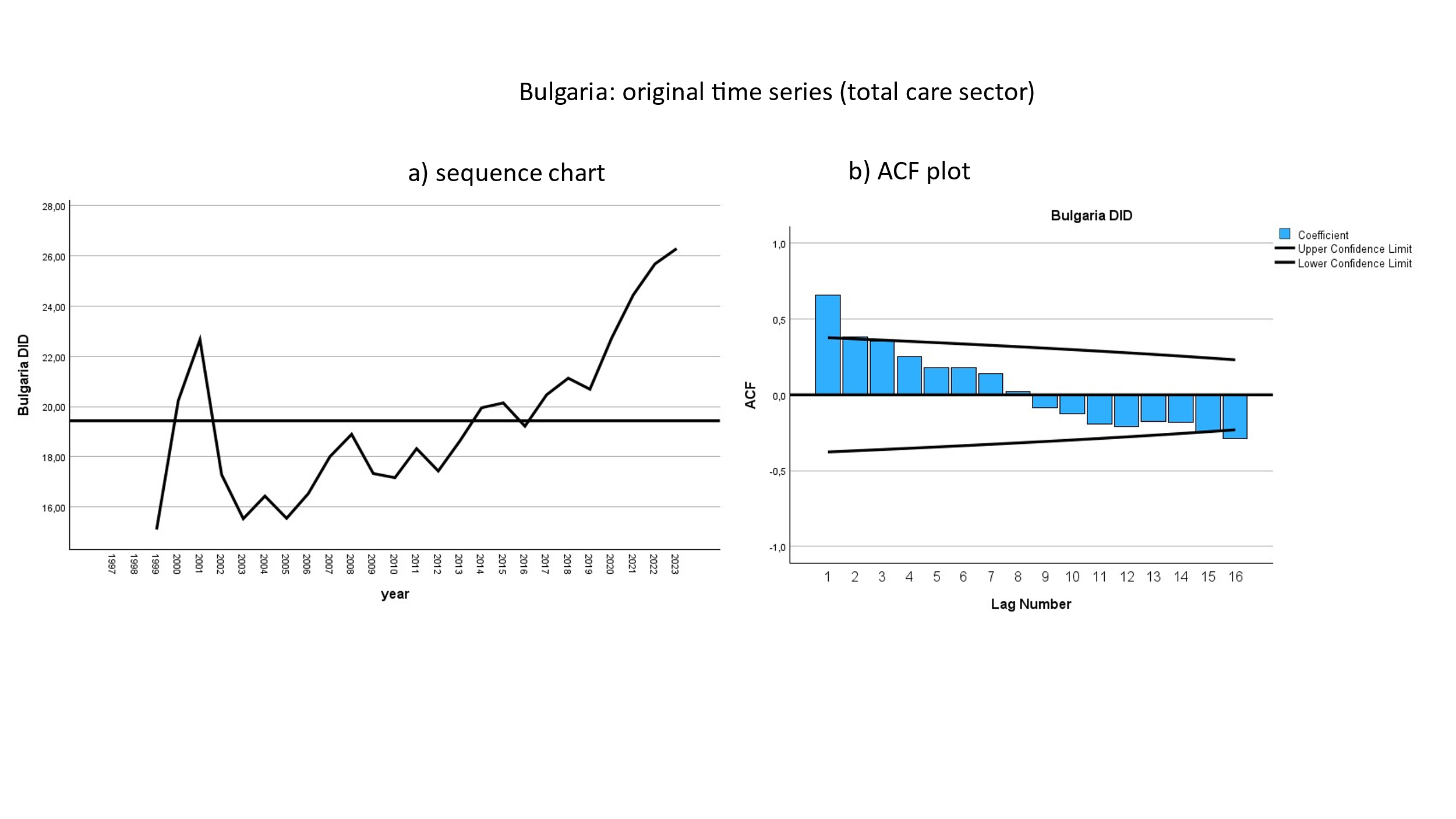


***Fig. S4:*** *Original time series of ATC class J01 for Croatia. In a) the sequence chart of consumption in DID is shown, while b) displays the ACF plot of the autocorrelation. The non-stationarity can be seen in the visible trend in both the sequence chart and the ACF plot.*


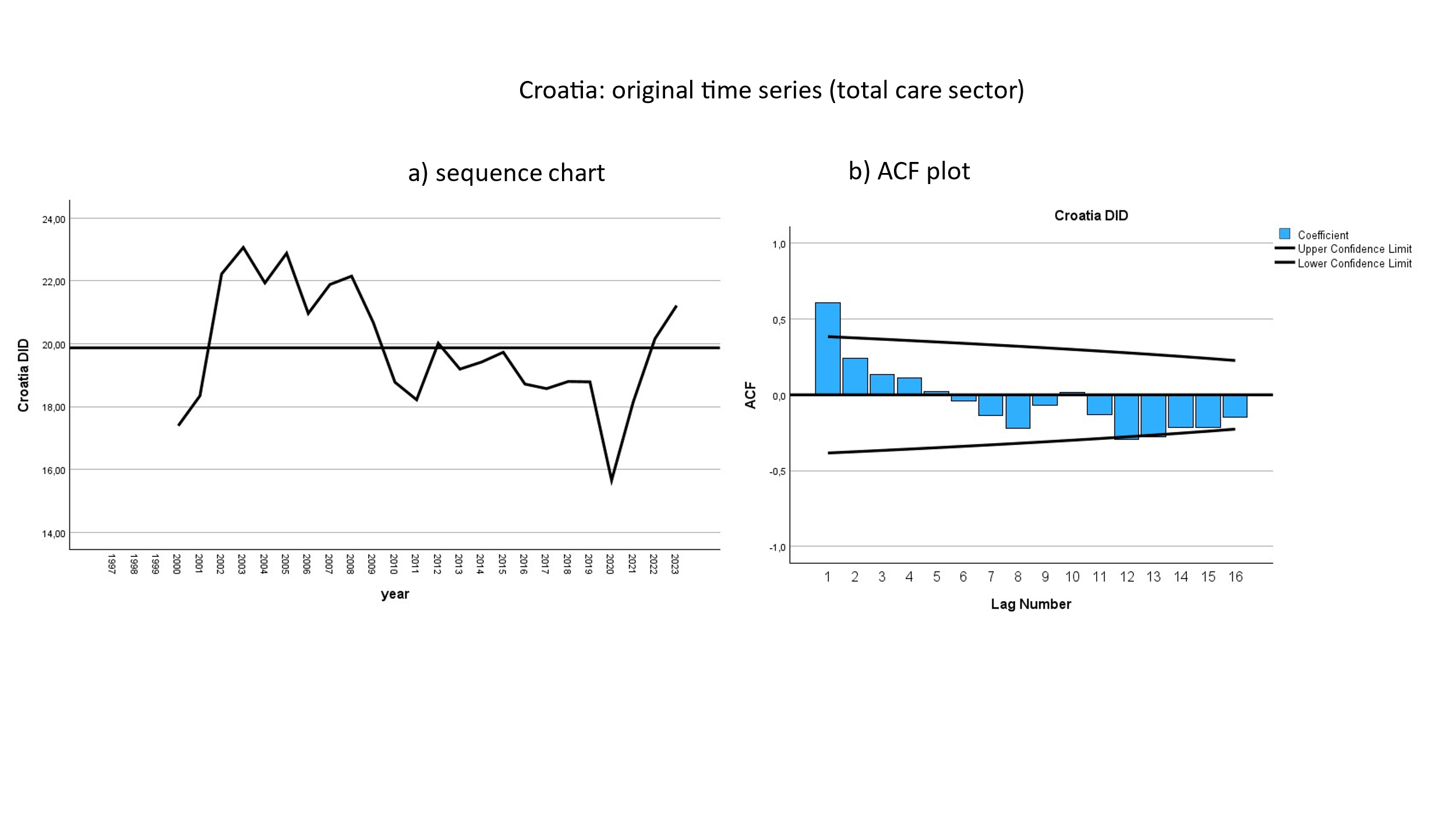


***Fig. S5:*** *Original time series of ATC class J01 for Czechia. In a) the sequence chart of consumption in DID is shown, while b) displays the ACF plot of the autocorrelation. The non-stationarity can be seen in the visible trend in both the sequence chart and the ACF plot.*


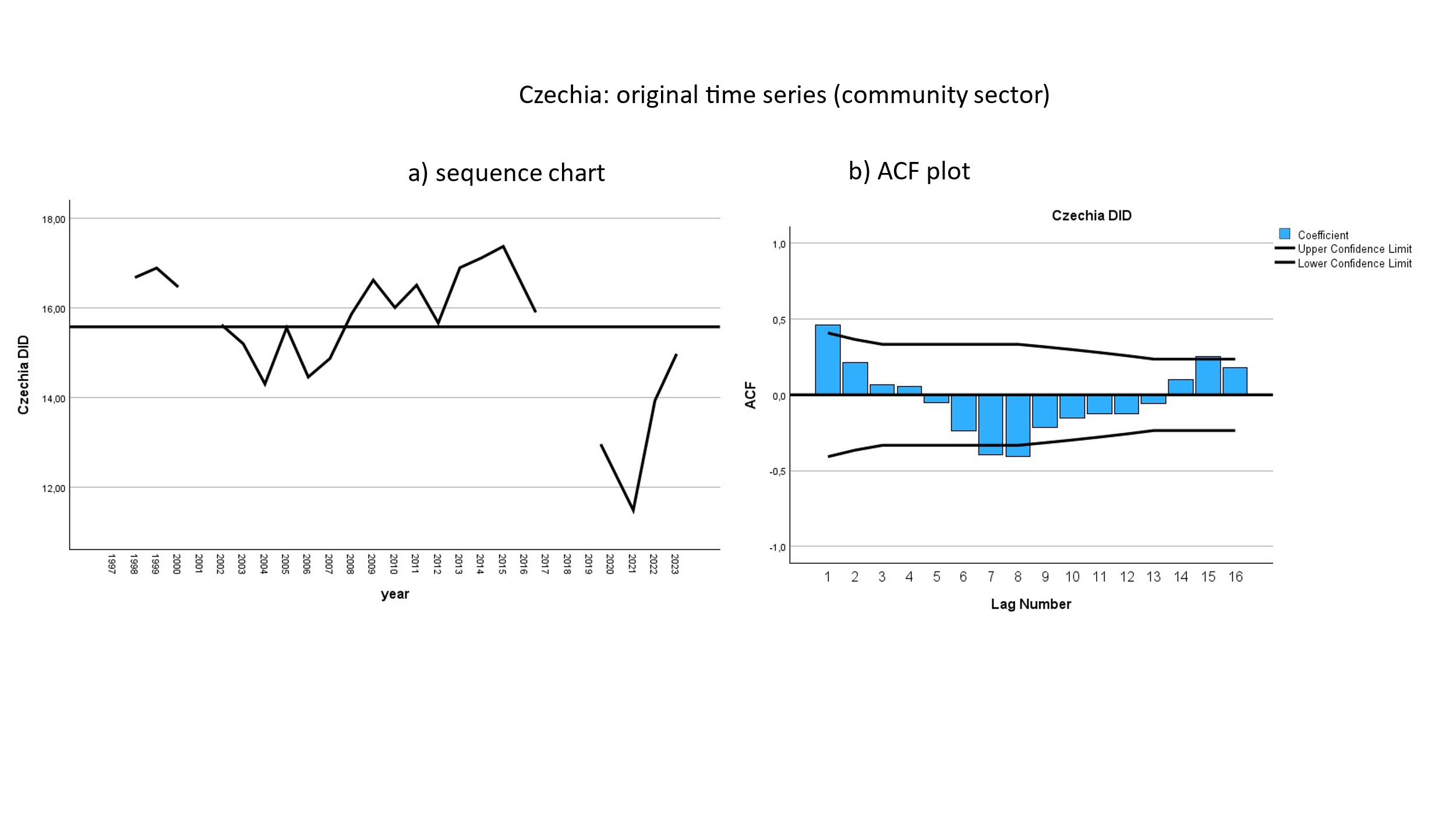


***Fig. S6:*** *Original time series of ATC class J01 for Denmark. In a) the sequence chart of consumption in DID is shown, while b) displays the ACF plot of the autocorrelation. The non-stationarity can be seen in the visible trend in both the sequence chart and the ACF plot.*


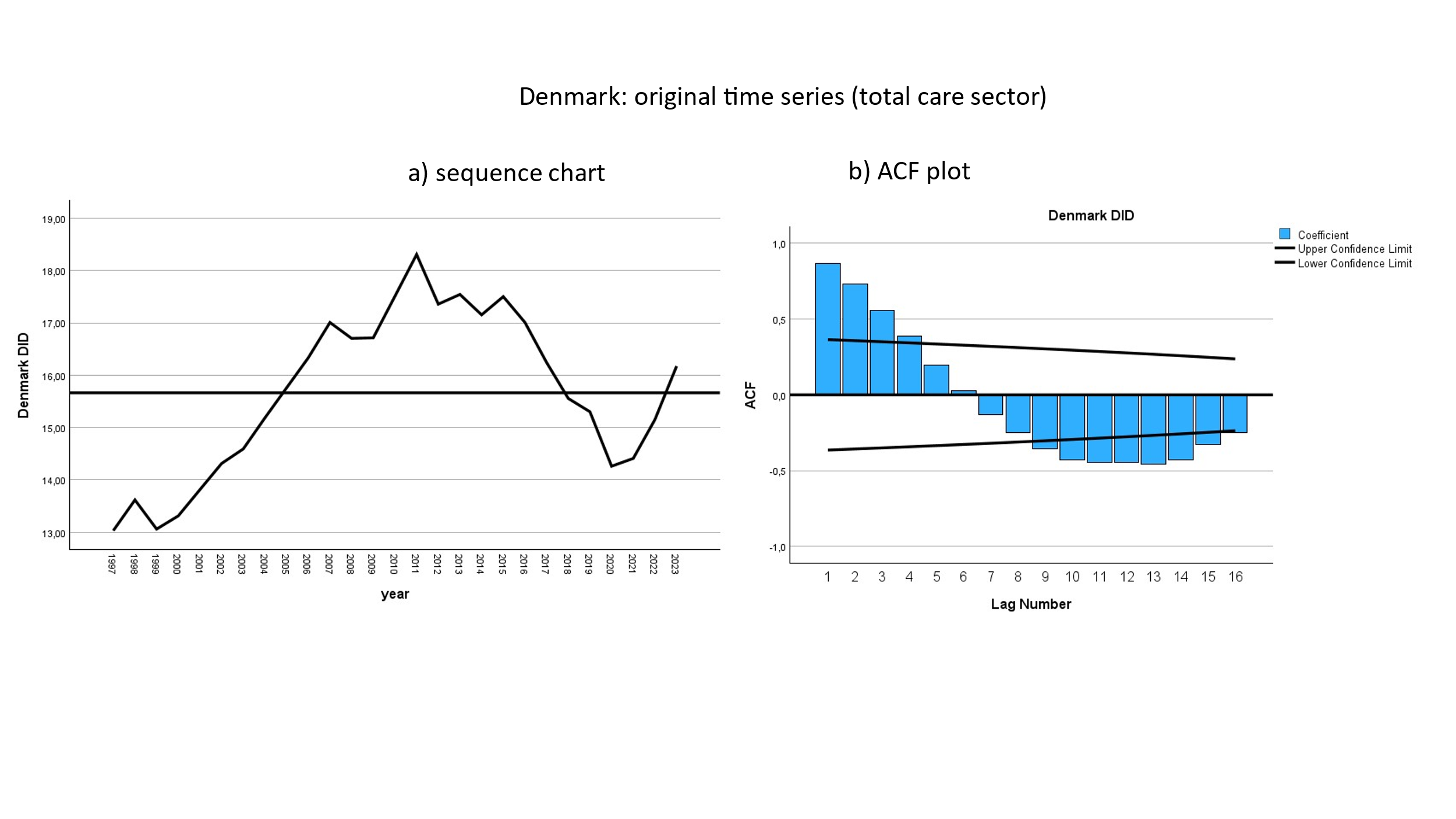


***Fig. S7:*** *Original time series of ATC class J01 for Estonia. In a) the sequence chart of consumption in DID is shown, while b) displays the ACF plot of the autocorrelation. The non-stationarity can be seen in the visible trend in both the sequence chart and the ACF plot.*


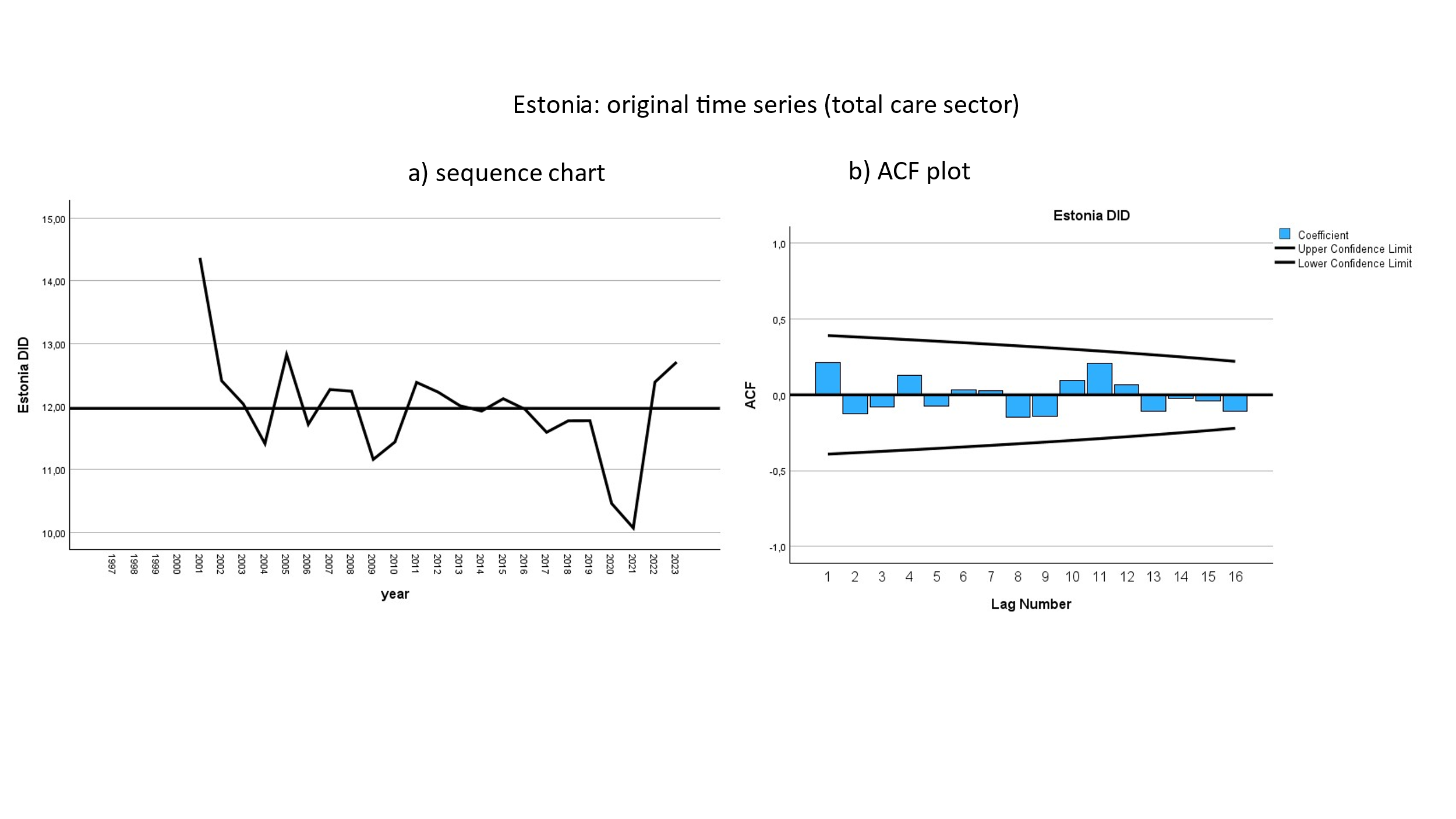


***Fig. S8:*** *Original time series of ATC class J01 for Finland. In a) the sequence chart of consumption in DID is shown, while b) displays the ACF plot of the autocorrelation. The non-stationarity can be seen in the visible trend in both the sequence chart and the ACF plot.*


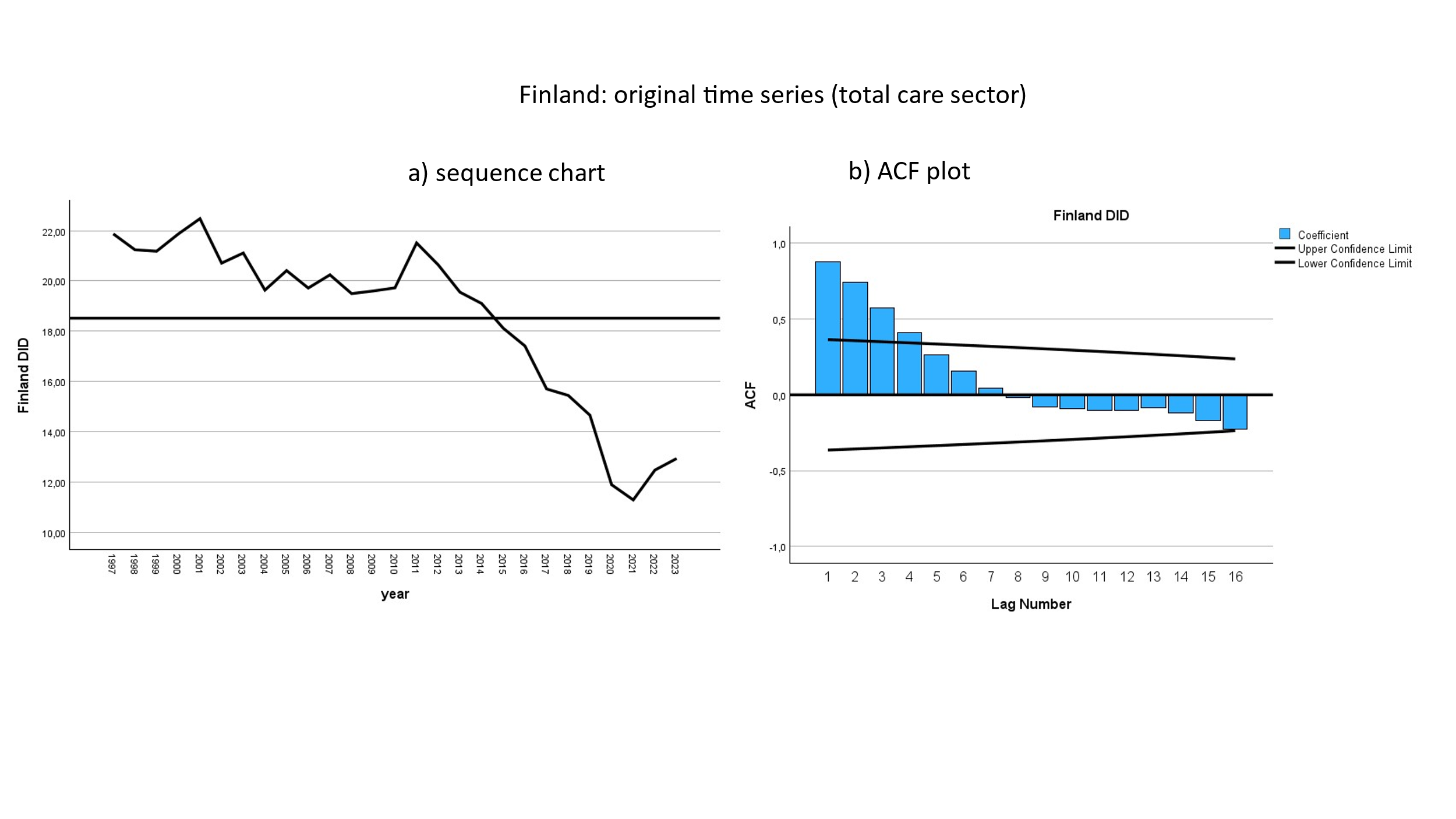


***Fig. S9:*** *Original time series of ATC class J01 for France. In a) the sequence chart of consumption in DID is shown, while b) displays the ACF plot of the autocorrelation. The non-stationarity can be seen in the visible trend in both the sequence chart and the ACF plot.*


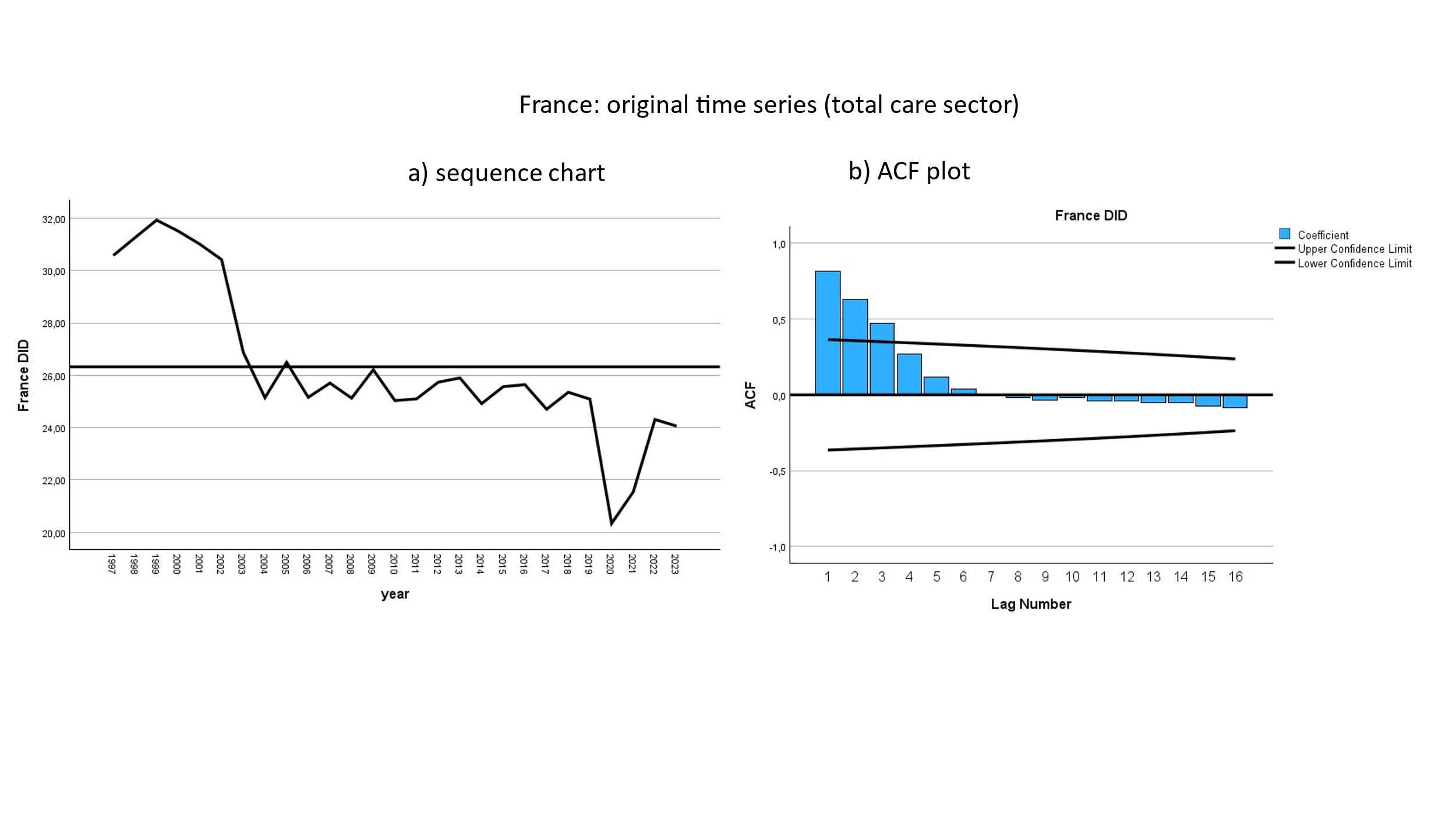


***Fig. S10:*** *Original time series of ATC class J01 for Germany. In a) the sequence chart of consumption in DID is shown, while b) displays the ACF plot of the autocorrelation. The non-stationarity can be seen in the visible trend in both the sequence chart and the ACF plot.*
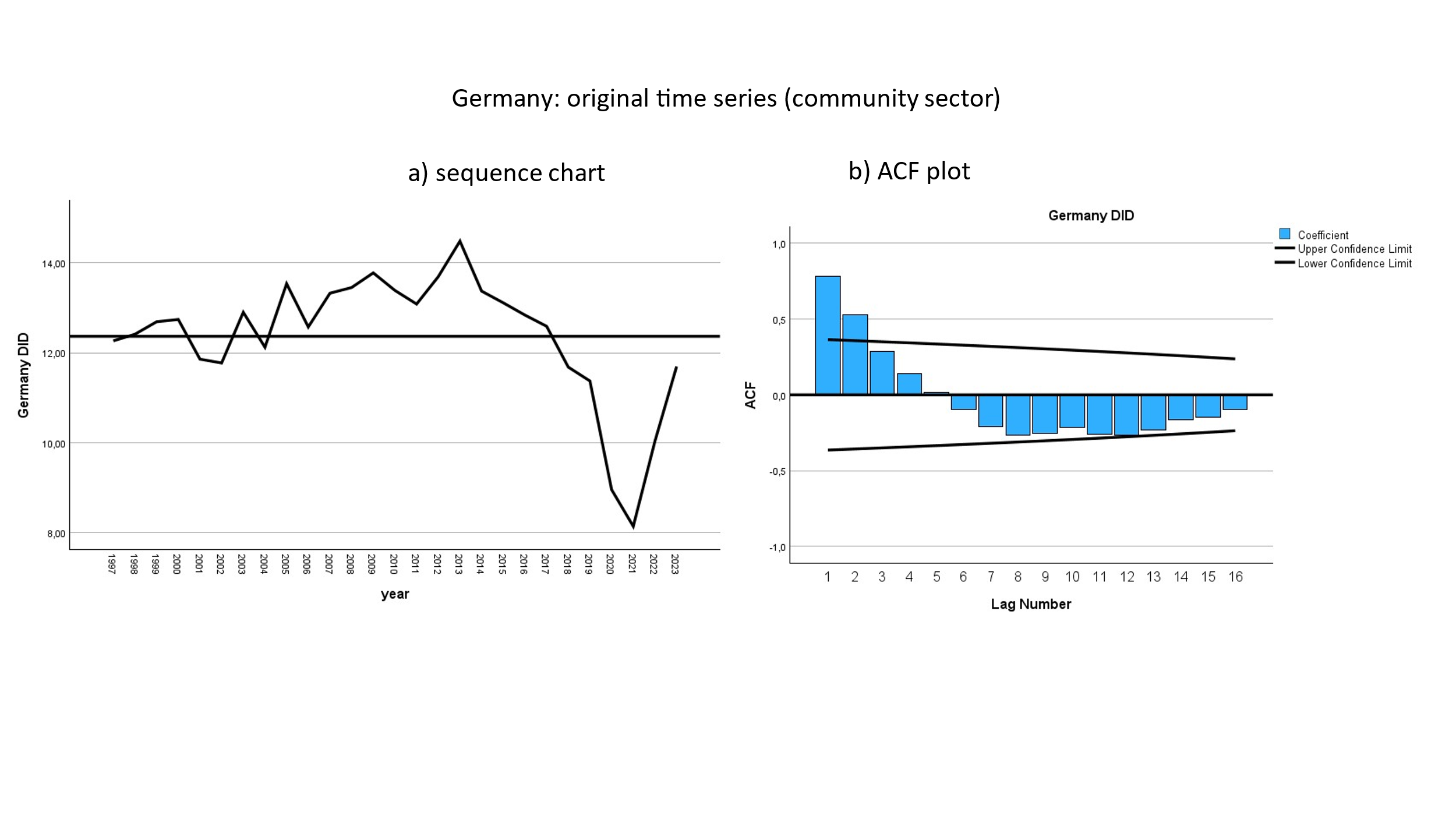


***Fig. S11:*** *Original time series of ATC class J01 for Greece. In a) the sequence chart of consumption in DID is shown, while b) displays the ACF plot of the autocorrelation. The non-stationarity can be seen in the visible trend in both the sequence chart and the ACF plot.*


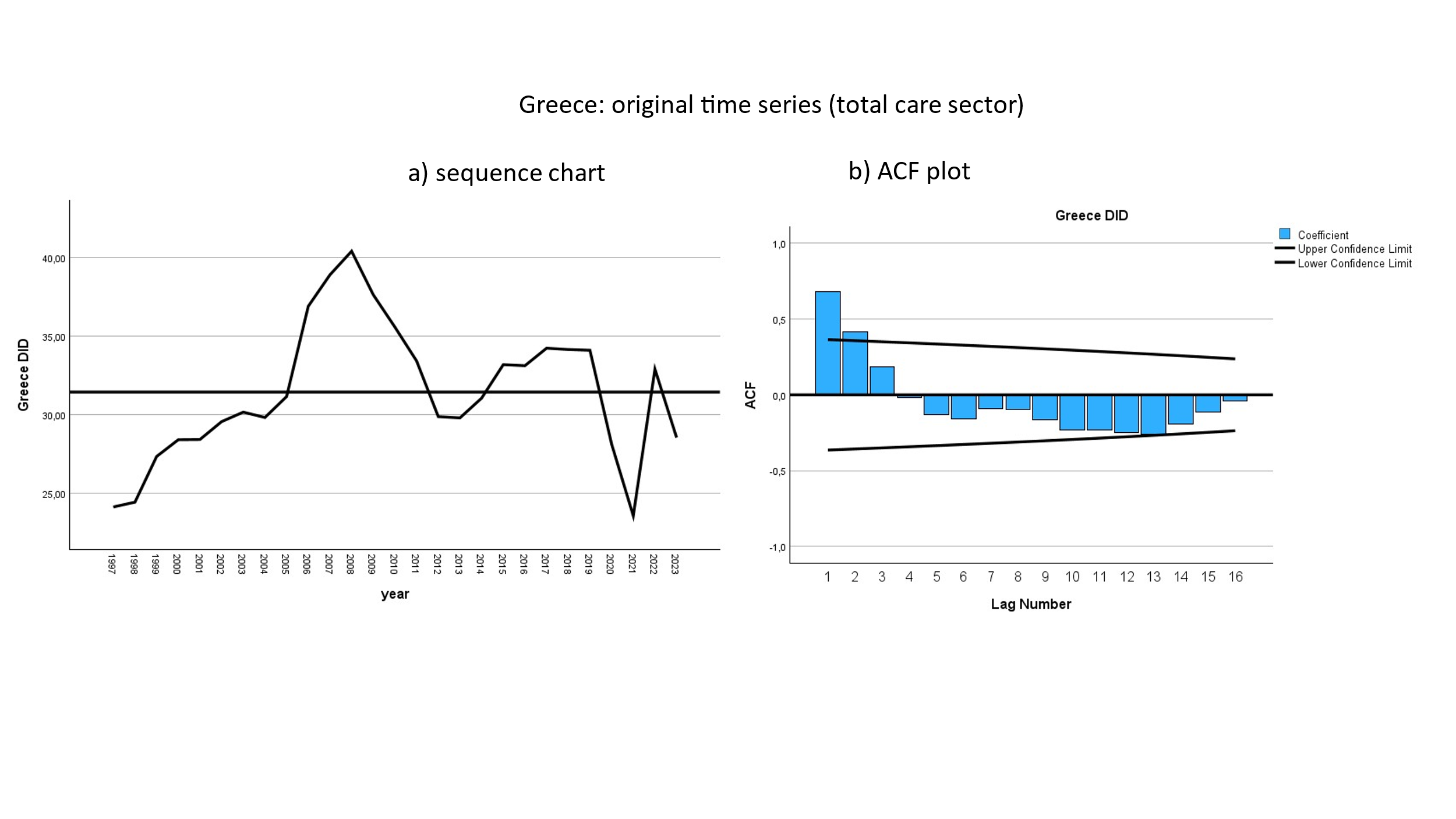


***Fig. S12:*** *Original time series of ATC class J01 for Hungary. In a) the sequence chart of consumption in DID is shown, while b) displays the ACF plot of the autocorrelation. The non-stationarity can be seen in the visible trend in both the sequence chart and the ACF plot.*
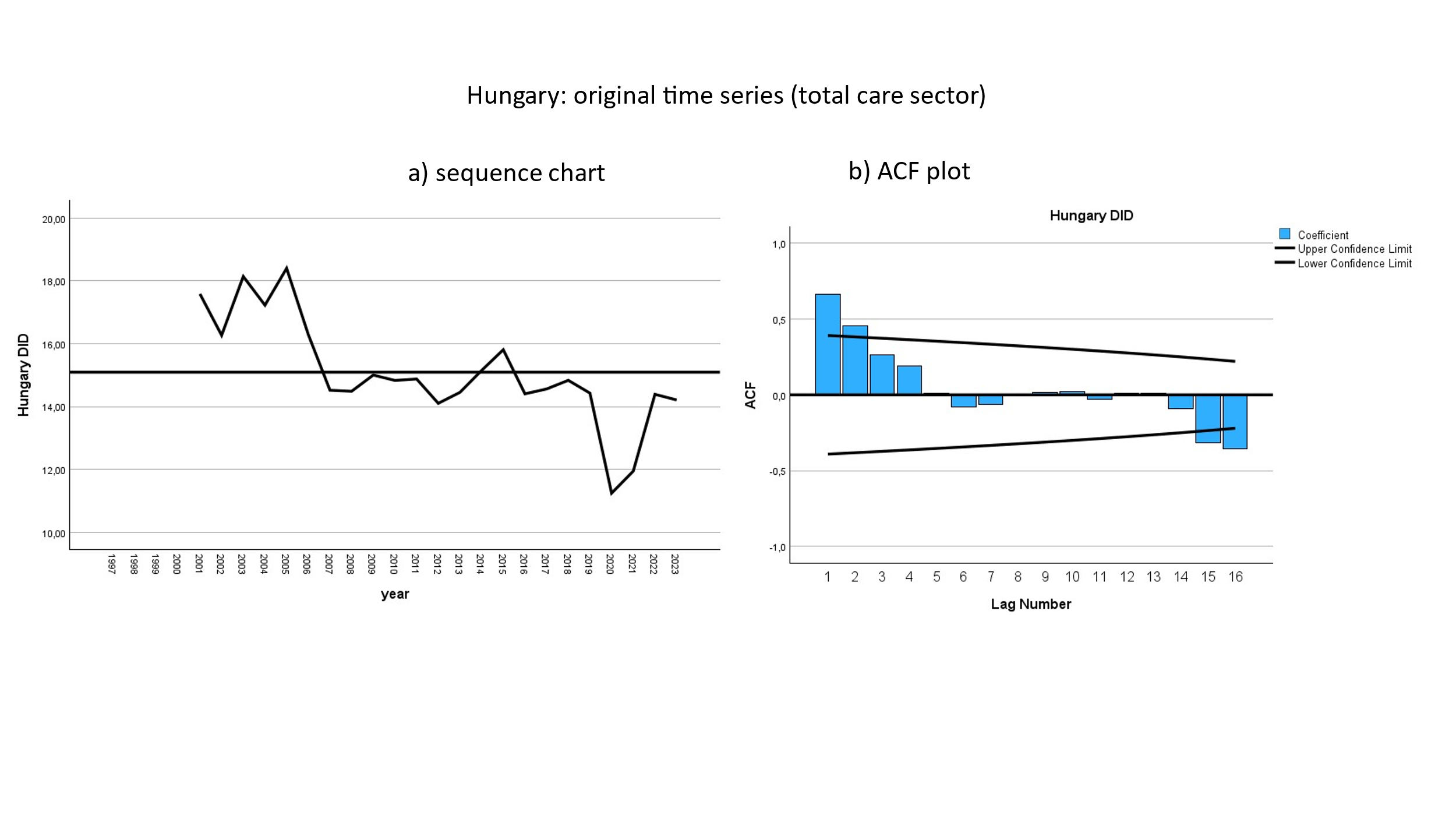


***Fig. S13:*** *Original time series of ATC class J01 for Ireland. In a) the sequence chart of consumption in DID is shown, while b) displays the ACF plot of the autocorrelation. The non-stationarity can be seen in the visible trend in both the sequence chart and the ACF plot.*


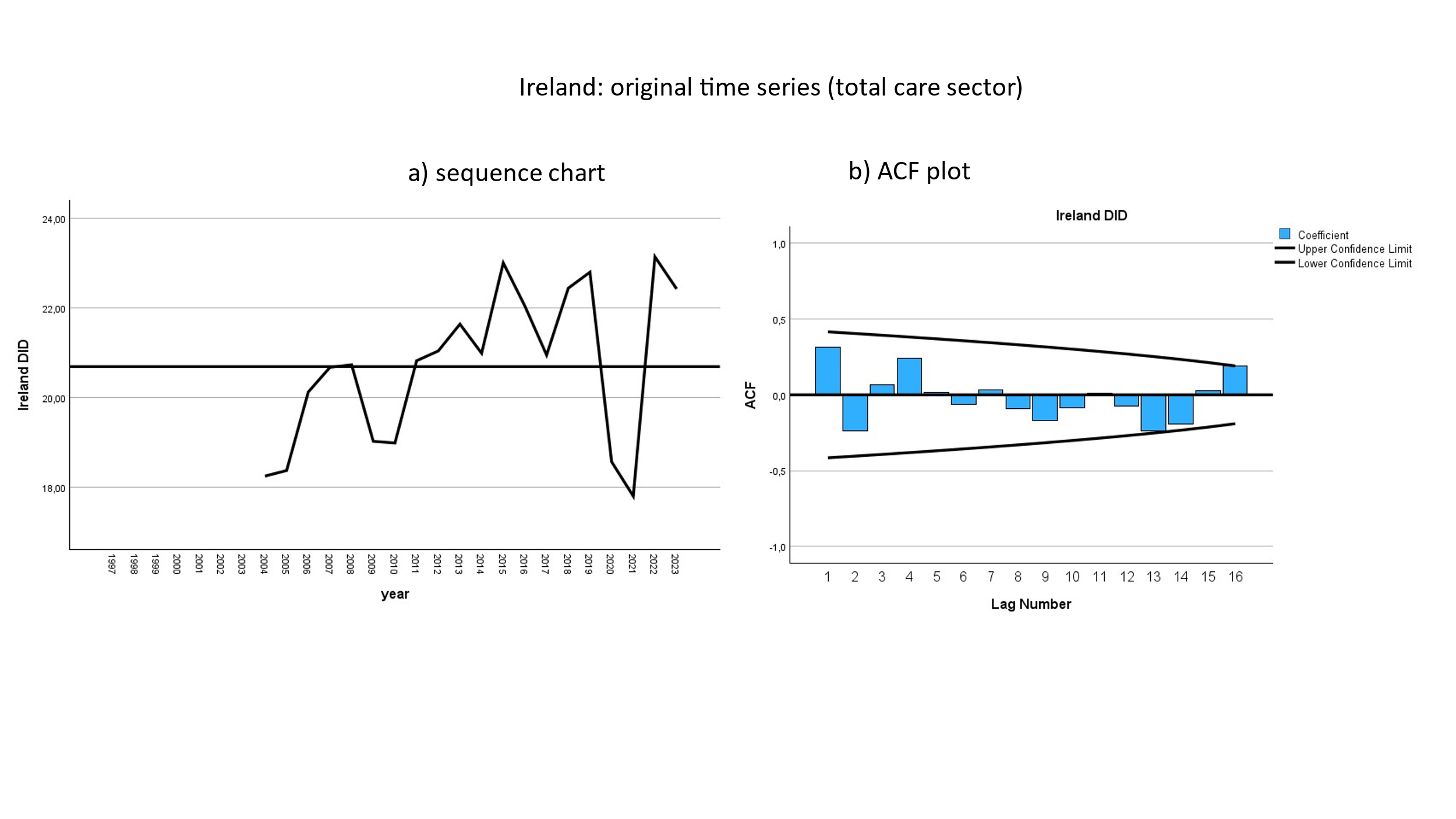


***Fig. S14:*** *Original time series of ATC class J01 for Italy. In a) the sequence chart of consumption in DID is shown, while b) displays the ACF plot of the autocorrelation. The non-stationarity can be seen in the visible trend in both the sequence chart and the ACF plot.*
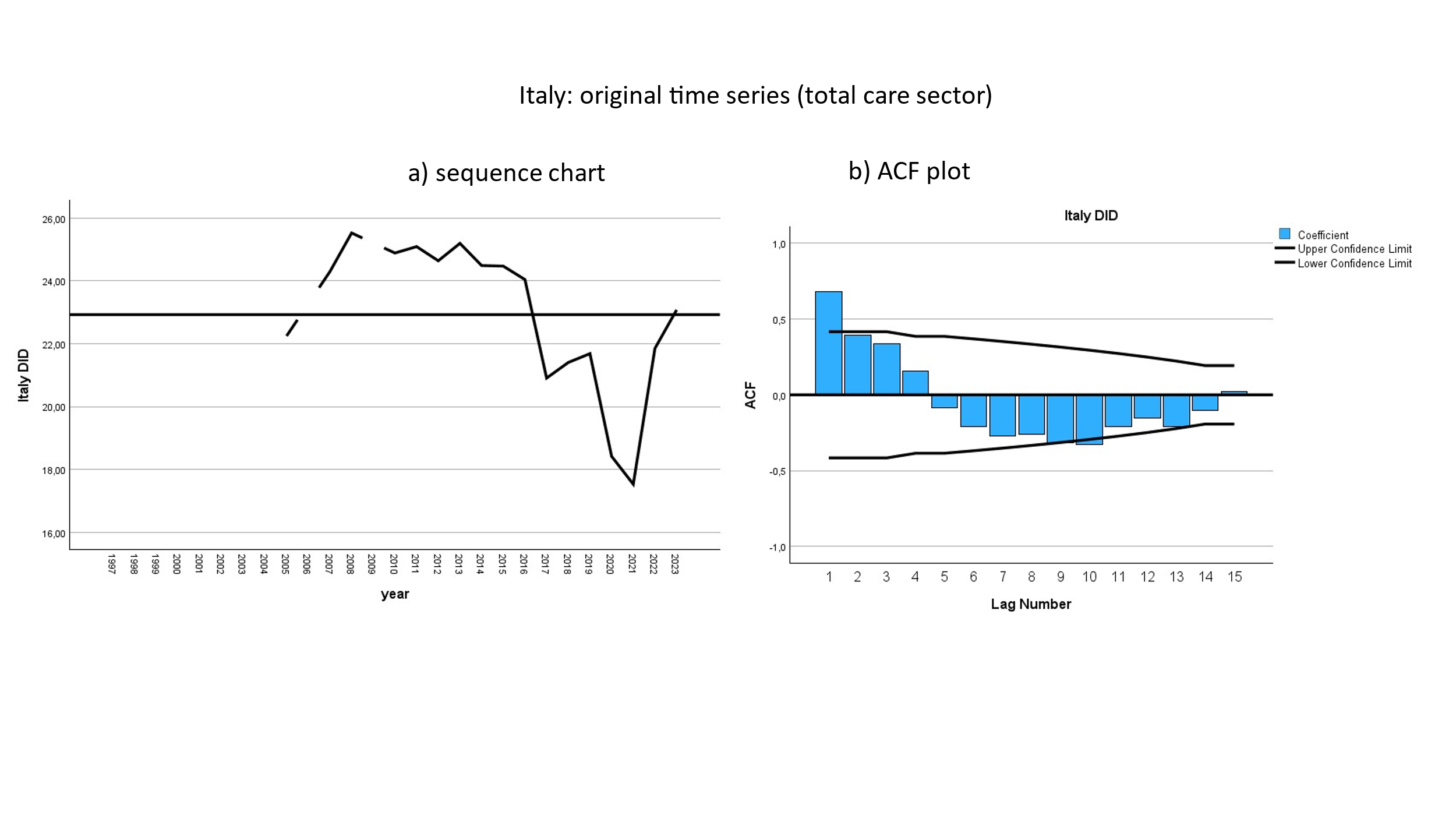


***Fig. S15:*** *Original time series of ATC class J01 for Iceland. In a) the sequence chart of consumption in DID is shown, while b) displays the ACF plot of the autocorrelation. The non-stationarity can be seen in the visible trend in both the sequence chart and the ACF plot.*


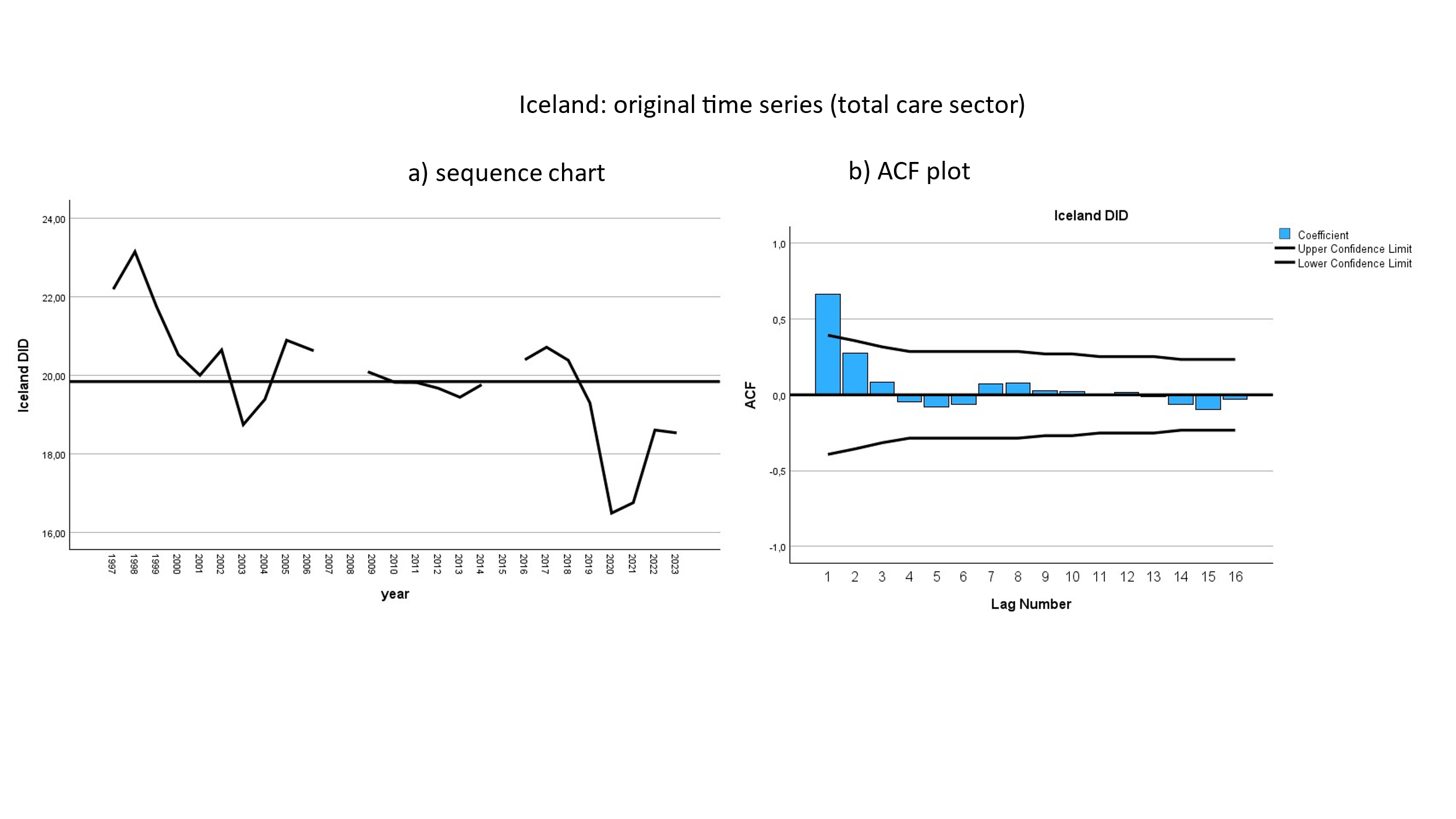


***Fig. S16:*** *Original time series of ATC class J01 for Lithuania. In a) the sequence chart of consumption in DID is shown, while b) displays the ACF plot of the autocorrelation. The non-stationarity can be seen in the visible trend in both the sequence chart and the ACF plot.*


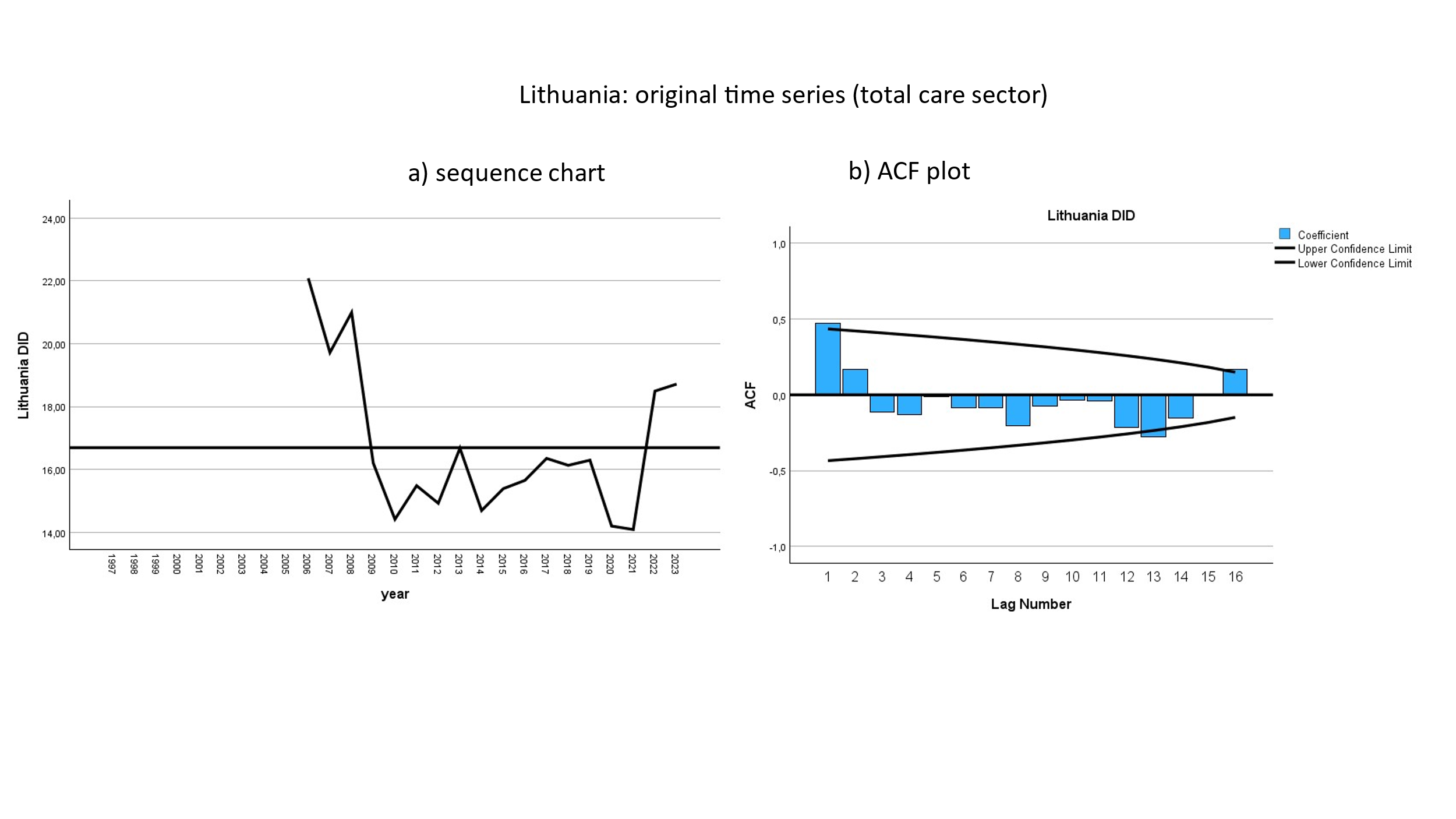


***Fig. S17:*** *Original time series of ATC class J01 for Luxembourg. In a) the sequence chart of consumption in DID is shown, while b) displays the ACF plot of the autocorrelation. The non-stationarity can be seen in the visible trend in both the sequence chart and the ACF plot.*


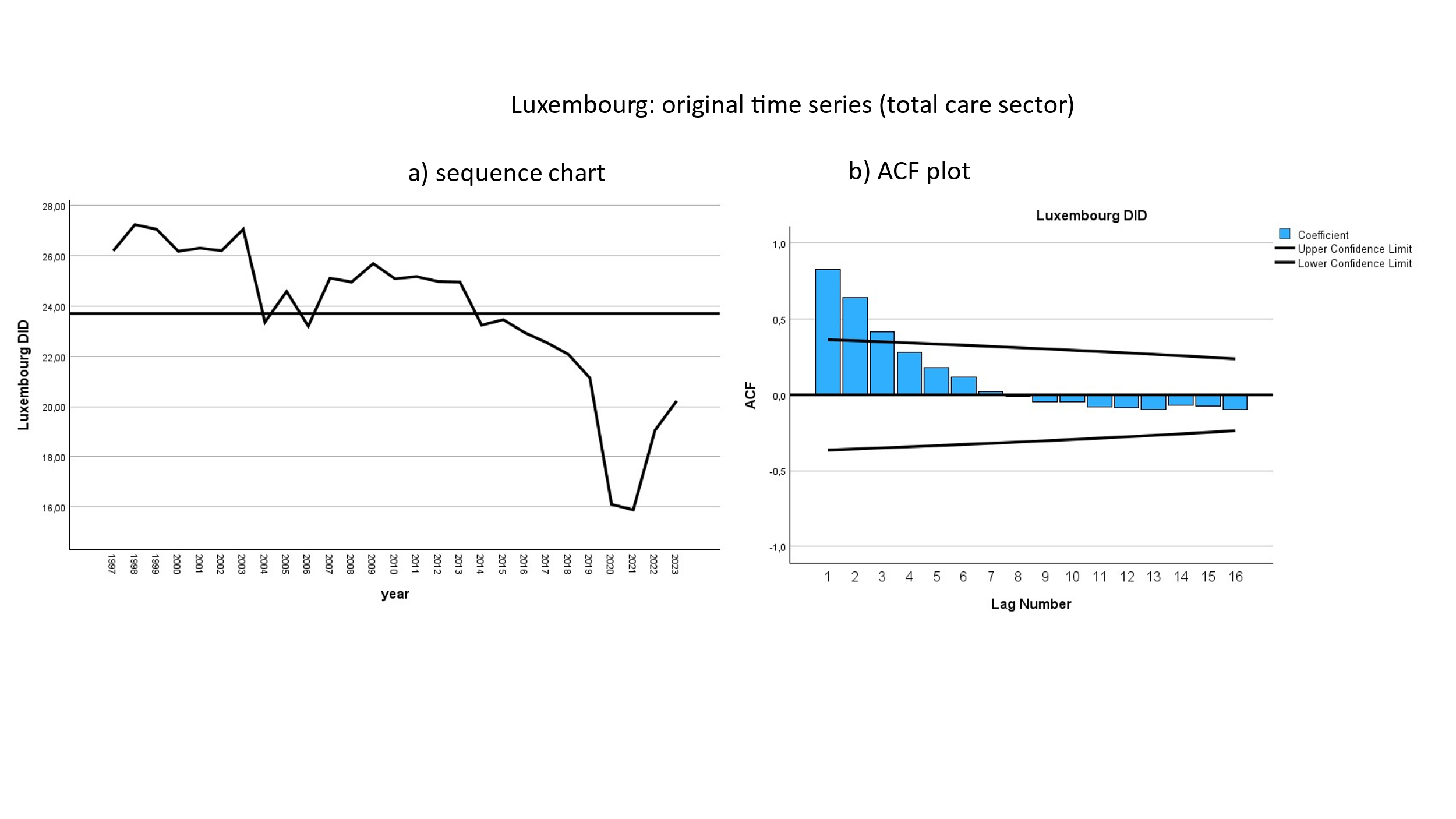


***Fig. S18:*** *Original time series of ATC class J01 for Latvia. In a) the sequence chart of consumption in DID is shown, while b) displays the ACF plot of the autocorrelation. The non-stationarity can be seen in the visible trend in both the sequence chart and the ACF plot.*


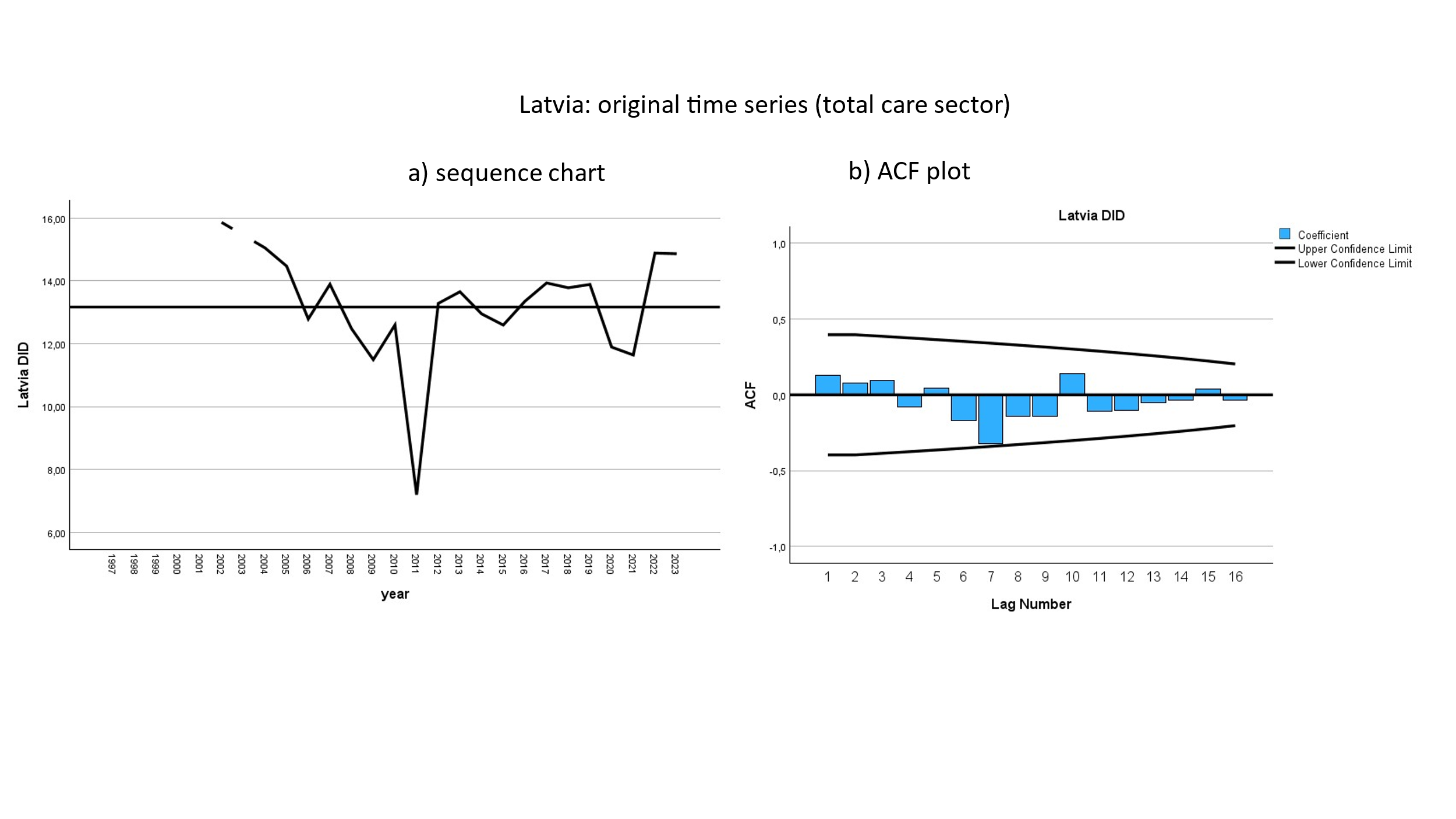


***Fig. S19:*** *Original time series of ATC class J01 for Malta. In a) the sequence chart of consumption in DID is shown, while b) displays the ACF plot of the autocorrelation. The non-stationarity can be seen in the visible trend in both the sequence chart and the ACF plot.*


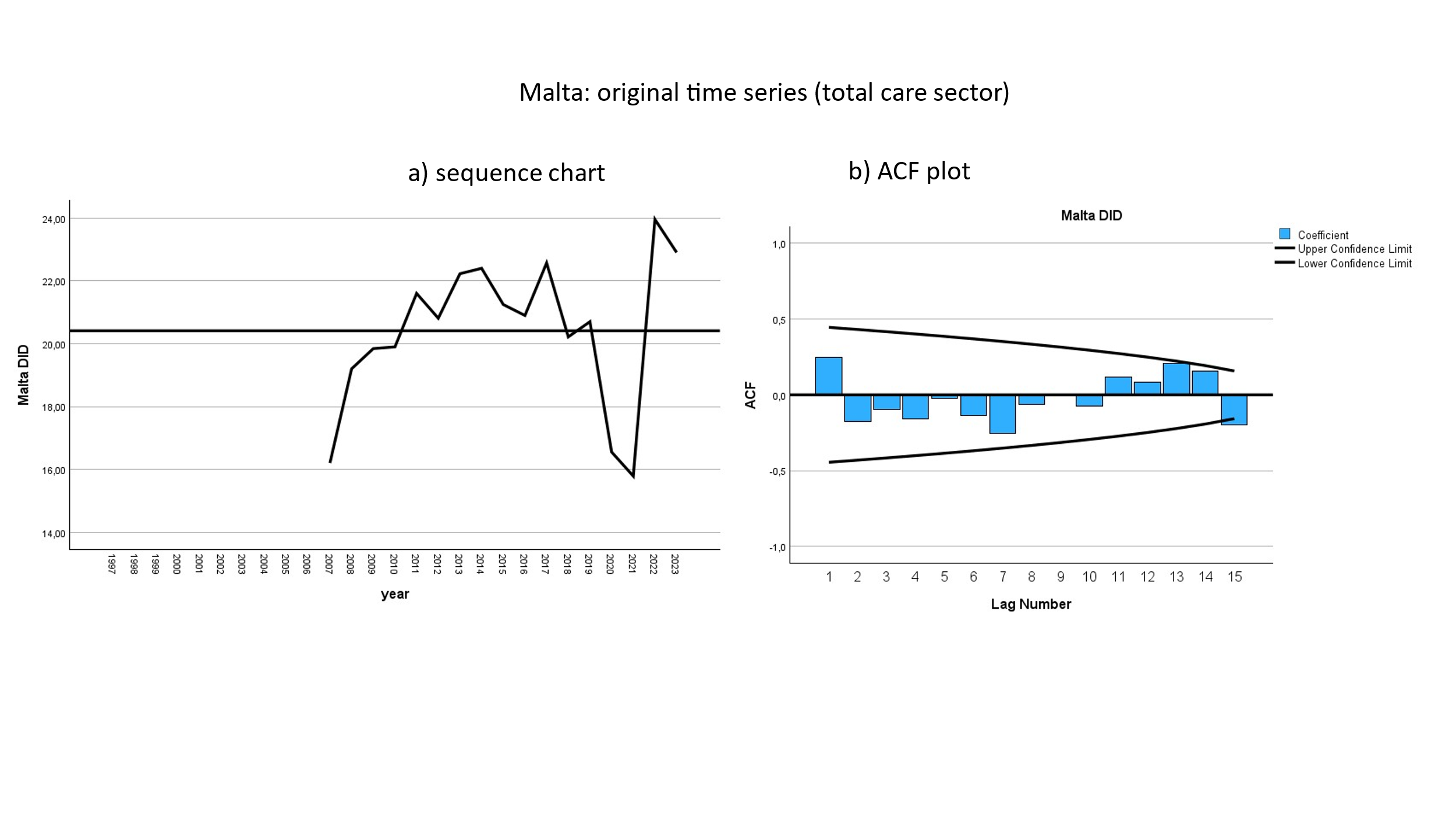


***Fig. S20:*** *Original time series of ATC class J01 for the Netherlands. In a) the sequence chart of consumption in DID is shown, while b) displays the ACF plot of the autocorrelation. The non-stationarity can be seen in the visible trend in both the sequence chart and the ACF plot.*


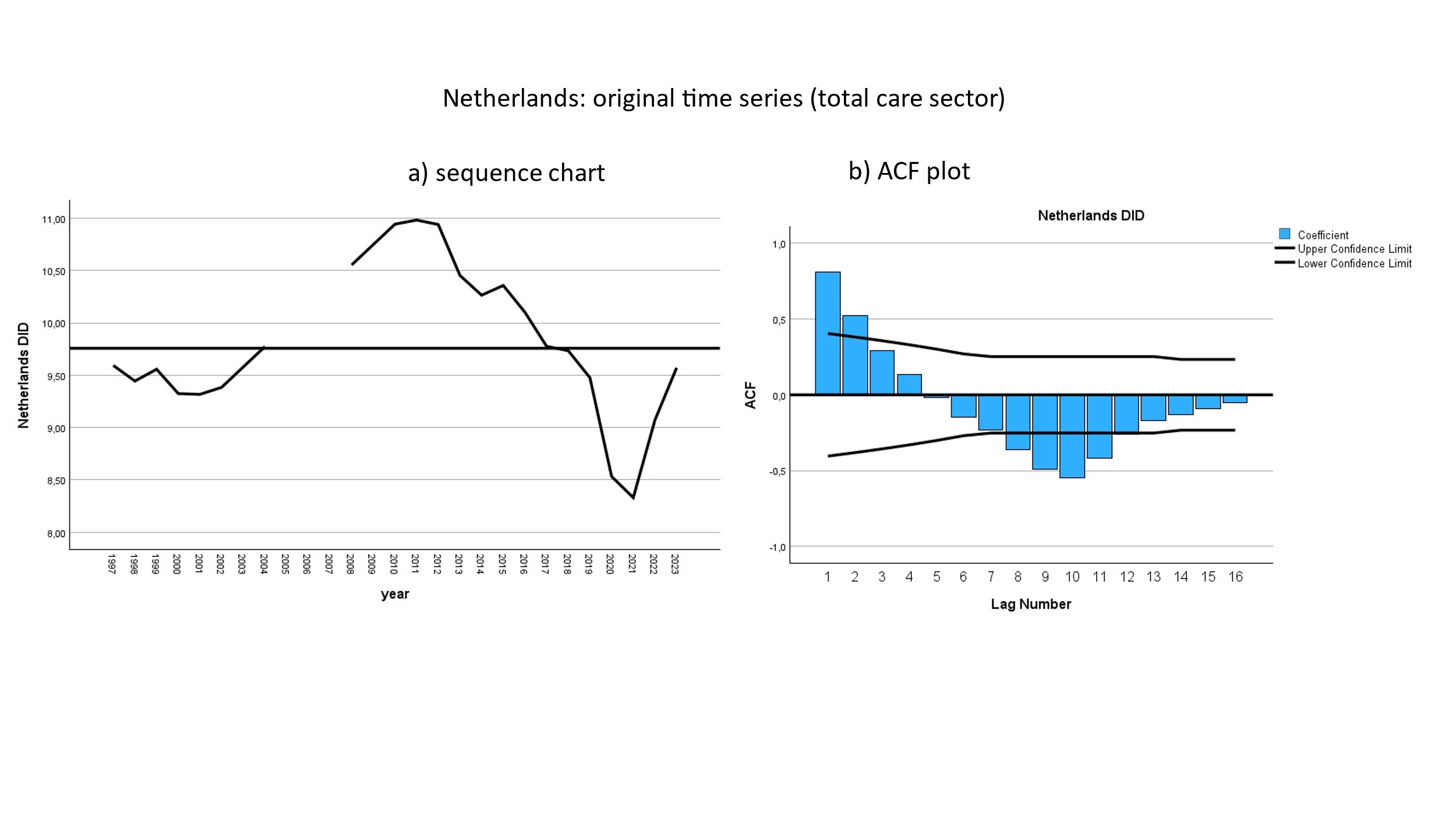


***Fig. S21:*** *Original time series of ATC class J01 for Norway. In a) the sequence chart of consumption in DID is shown, while b) displays the ACF plot of the autocorrelation. The non-stationarity can be seen in the visible trend in both the sequence chart and the ACF plot.*


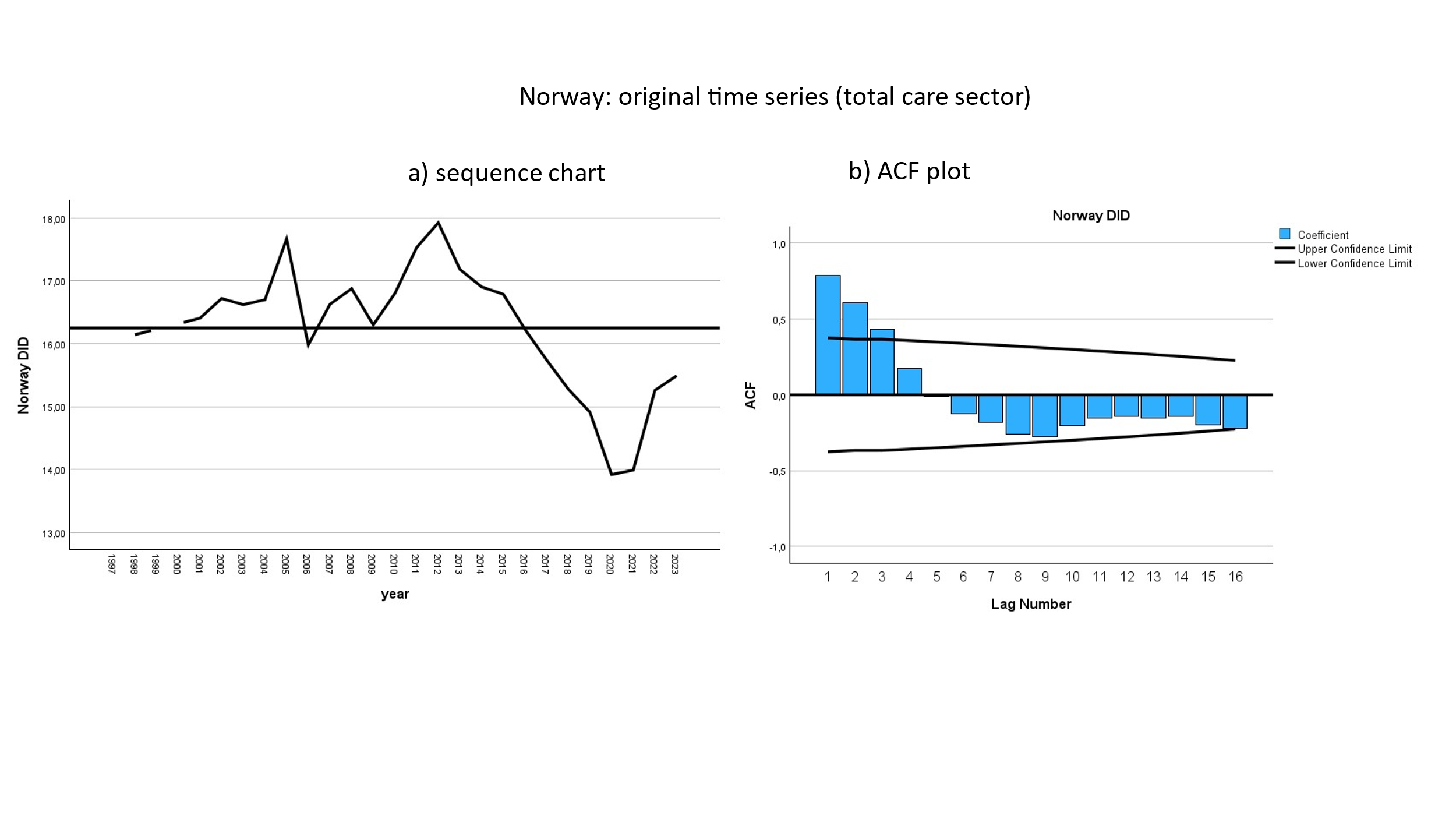


***Fig. S22:*** *Original time series of ATC class J01 for Poland. In a) the sequence chart of consumption in DID is shown, while b) displays the ACF plot of the autocorrelation. The non-stationarity can be seen in the visible trend in both the sequence chart and the ACF plot.*


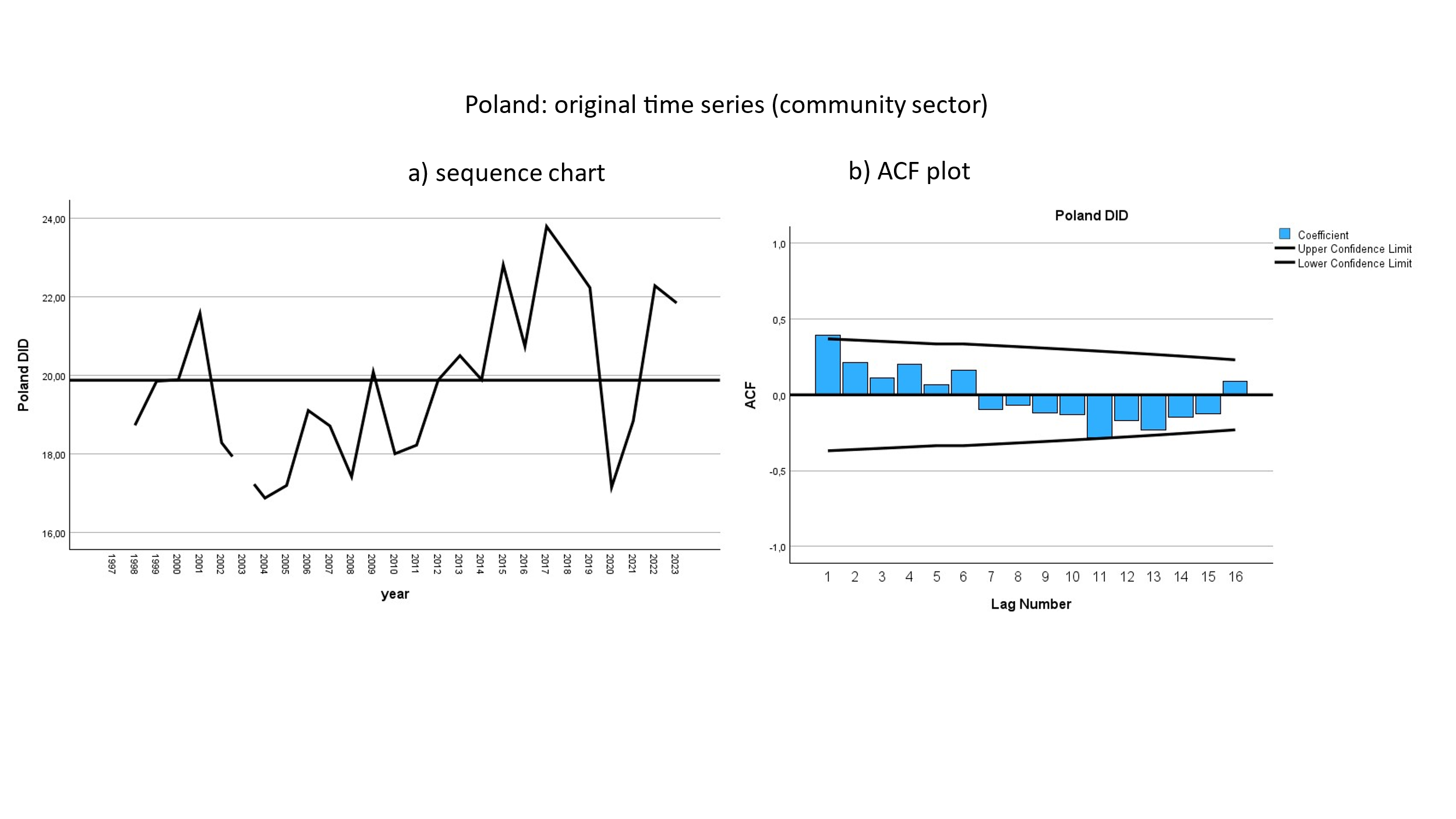


***Fig. S23:*** *Original time series of ATC class J01 for Portugal. In a) the sequence chart of consumption in DID is shown, while b) displays the ACF plot of the autocorrelation. The non-stationarity can be seen in the visible trend in both the sequence chart and the ACF plot.*


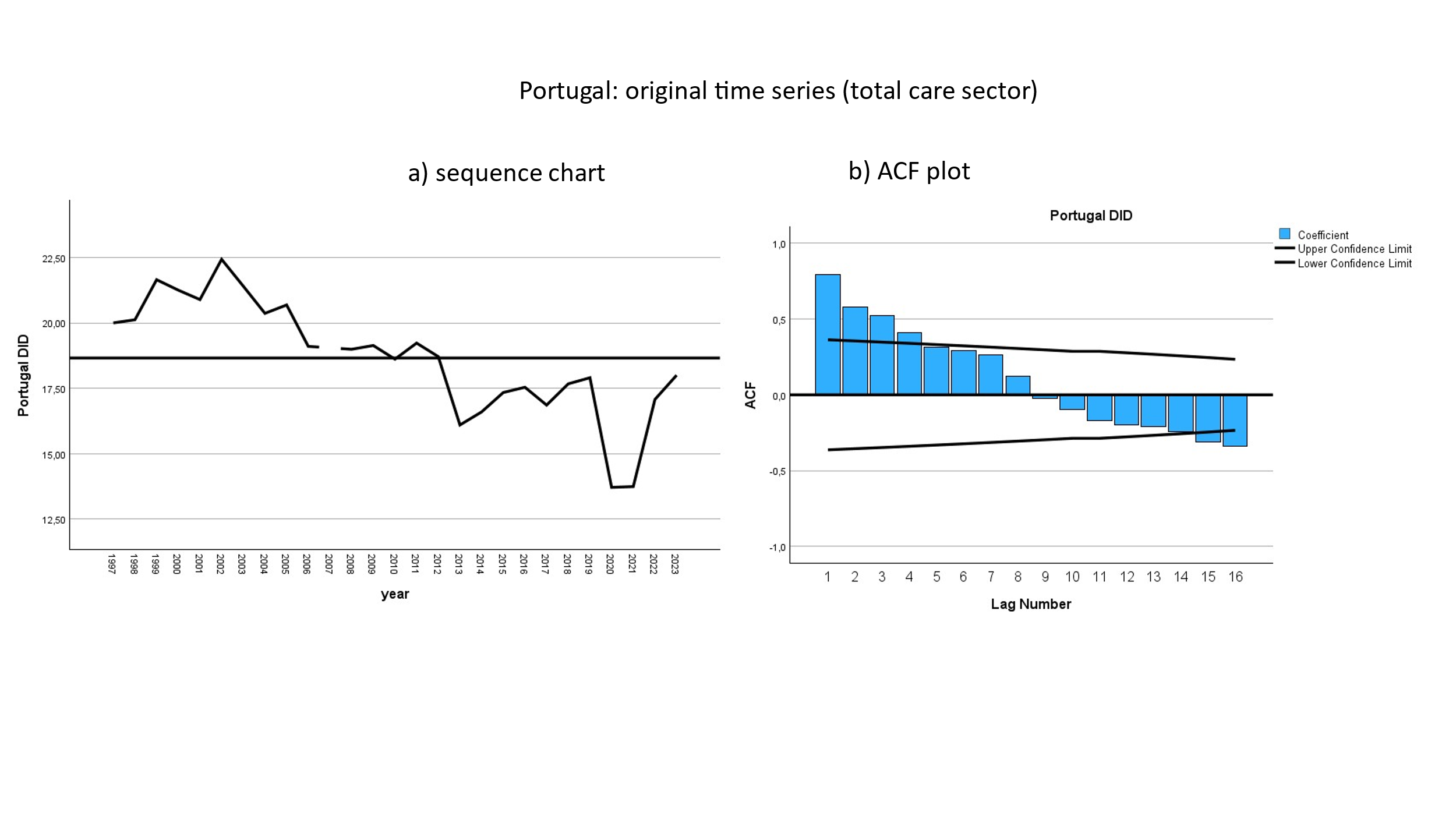


***Fig. S24:*** *Original time series of ATC class J01 for Romania. In a) the sequence chart of consumption in DID is shown, while b) displays the ACF plot of the autocorrelation. The non-stationarity can be seen in the visible trend in both the sequence chart and the ACF plot.*


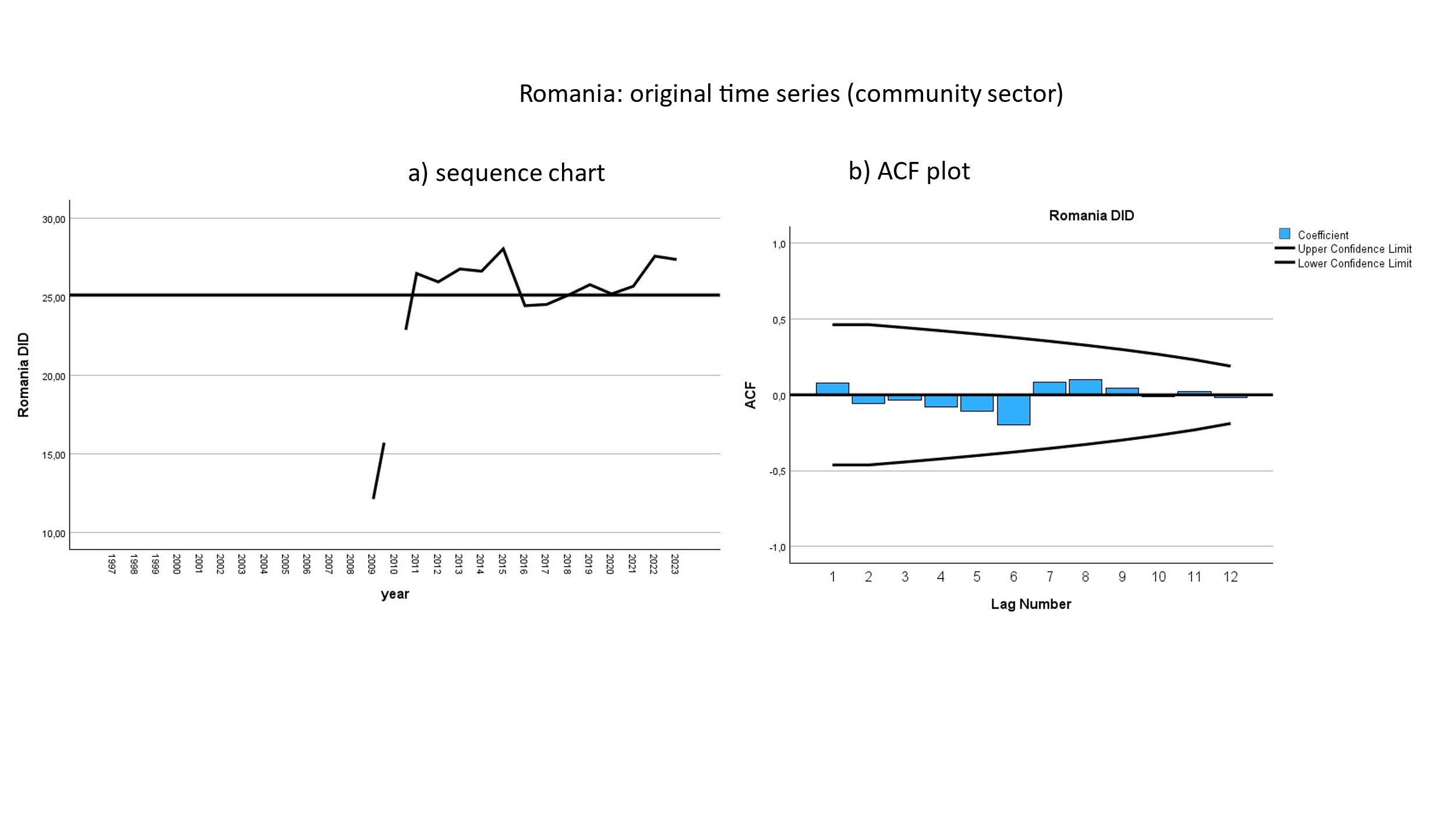


***Fig. S25:*** *Original time series of ATC class J01 for Slovenia. In a) the sequence chart of consumption in DID is shown, while b) displays the ACF plot of the autocorrelation. The non-stationarity can be seen in the visible trend in both the sequence chart and the ACF plot.*


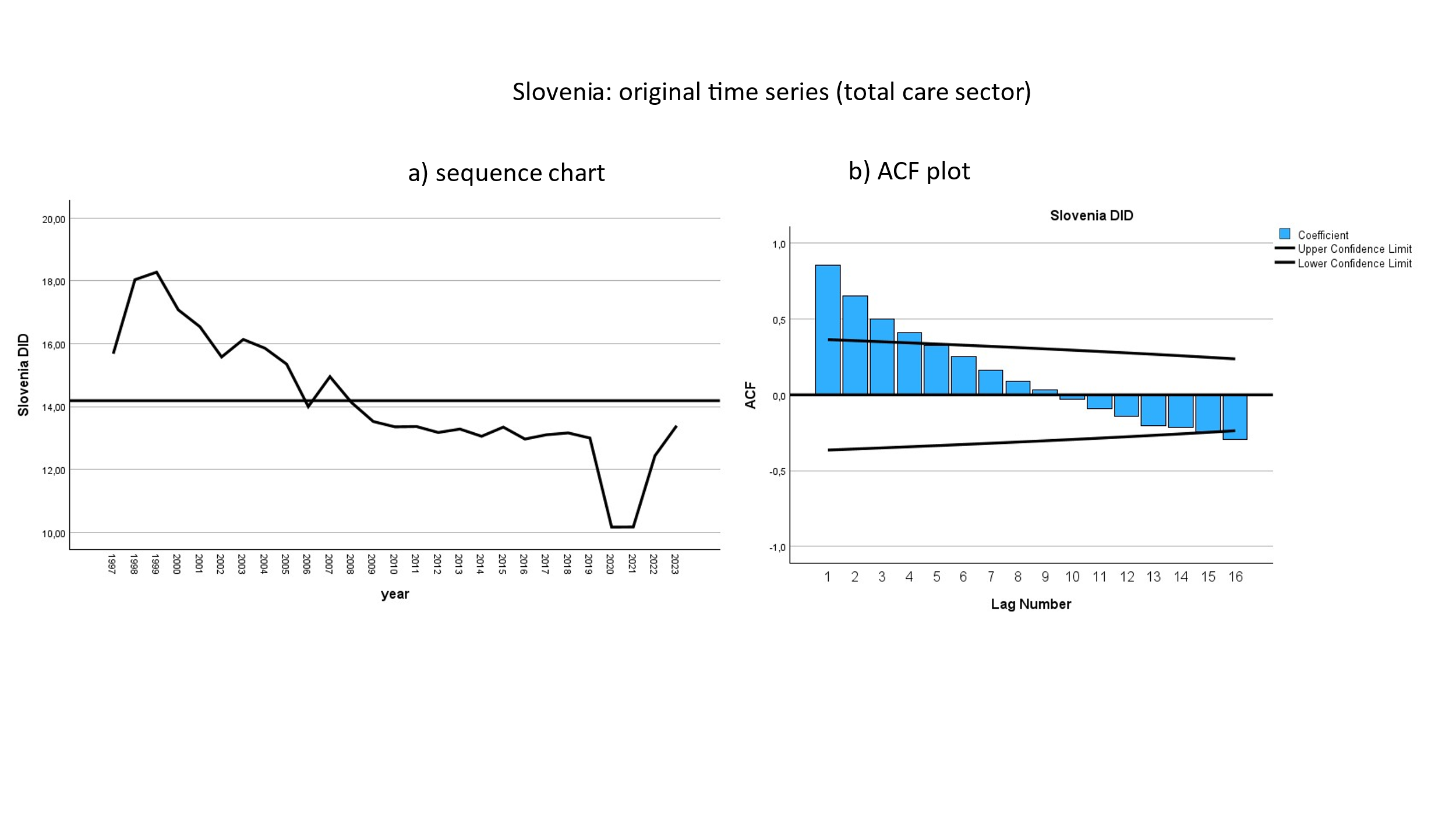


***Fig. S26:*** *Original time series of ATC class J01 for Slovakia. In a) the sequence chart of consumption in DID is shown, while b) displays the ACF plot of the autocorrelation. The non-stationarity can be seen in the visible trend in both the sequence chart and the ACF plot.*


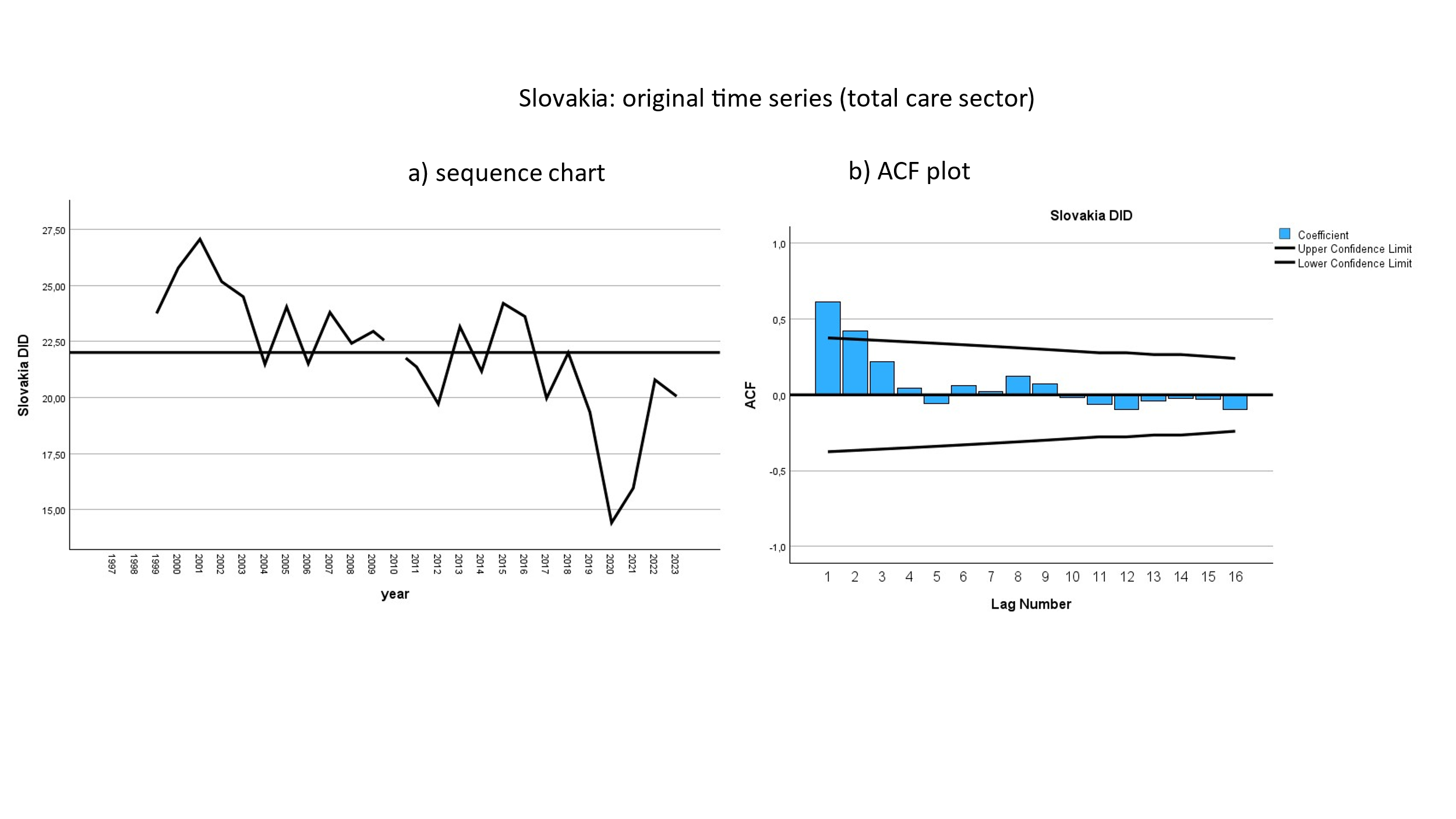


***Fig. S27:*** *Original time series of ATC class J01 for Spain. In a) the sequence chart of consumption in DID is shown, while b) displays the ACF plot of the autocorrelation. The non-stationarity can be seen in the visible trend in both the sequence chart and the ACF plot.*


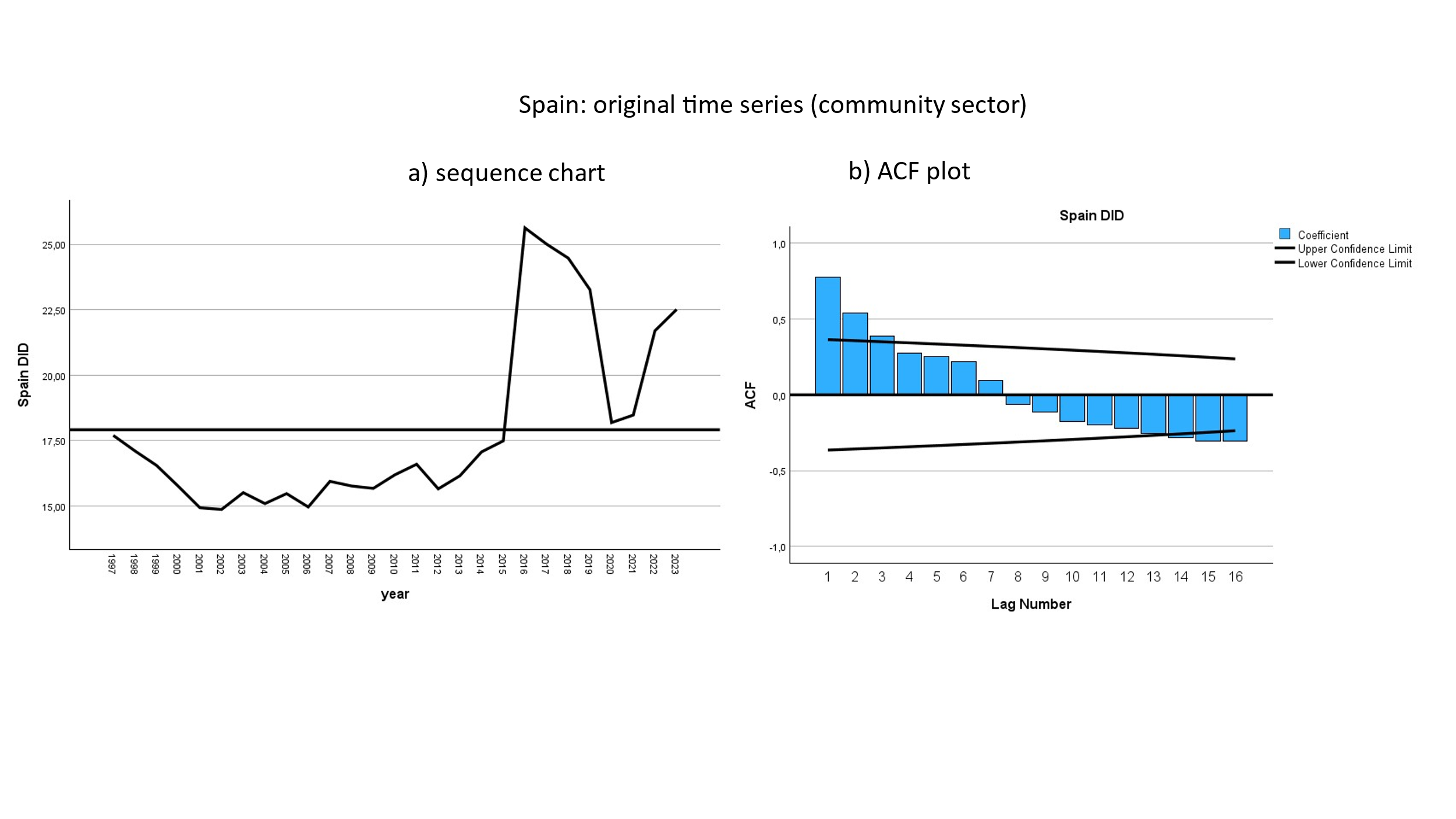


***Fig. S28:*** *Original time series of ATC class J01 for Sweden. In a) the sequence chart of consumption in DID is shown, while b) displays the ACF plot of the autocorrelation. The non-stationarity can be seen in the visible trend in both the sequence chart and the ACF plot.*


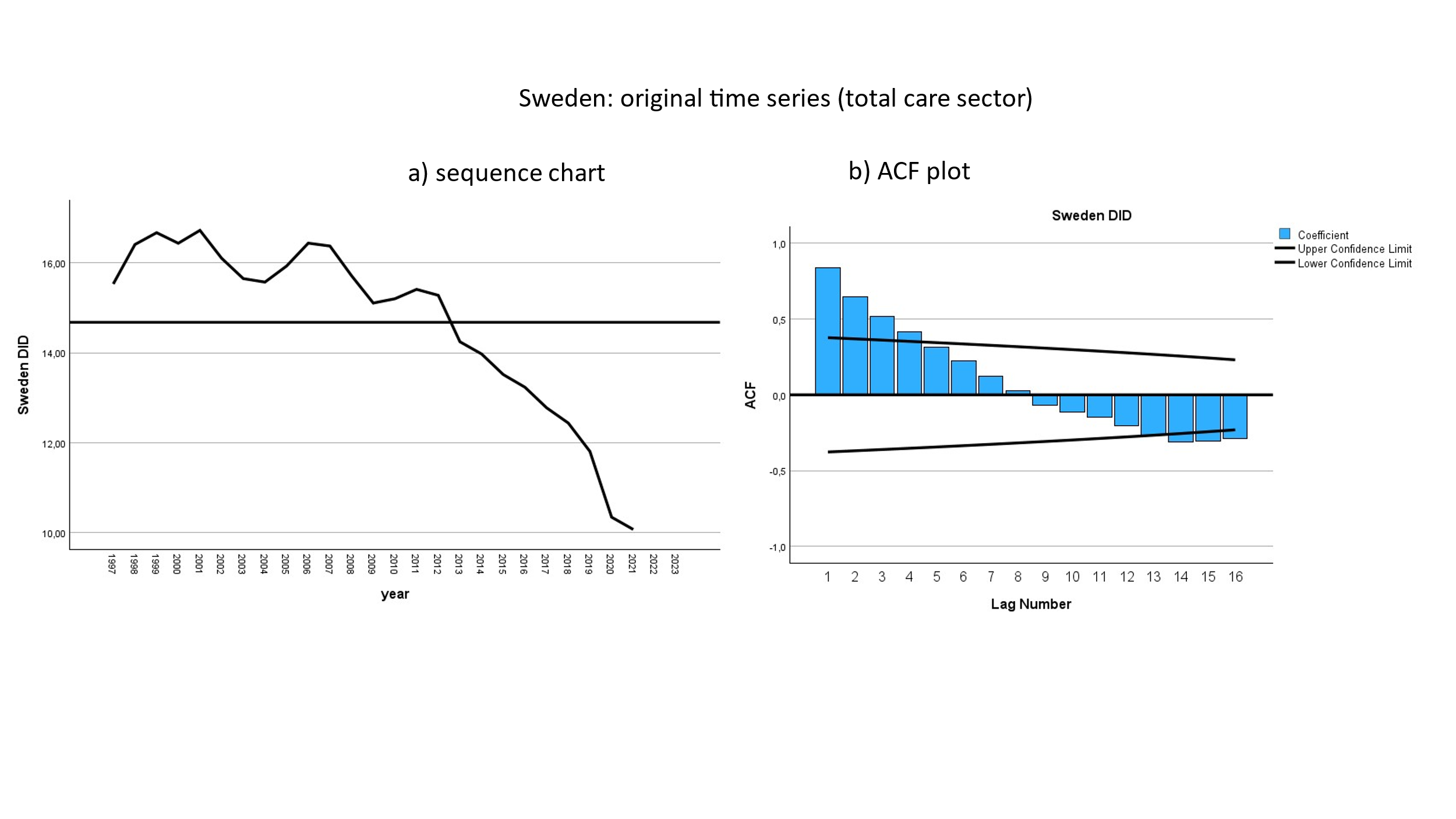


***Fig. S29:*** *Original time series of ATC class J01 for the United Kingdom. In a) the sequence chart of consumption in DID is shown, while b) displays the ACF plot of the autocorrelation. The non-stationarity can be seen in the visible trend in both the sequence chart and the ACF plot.*


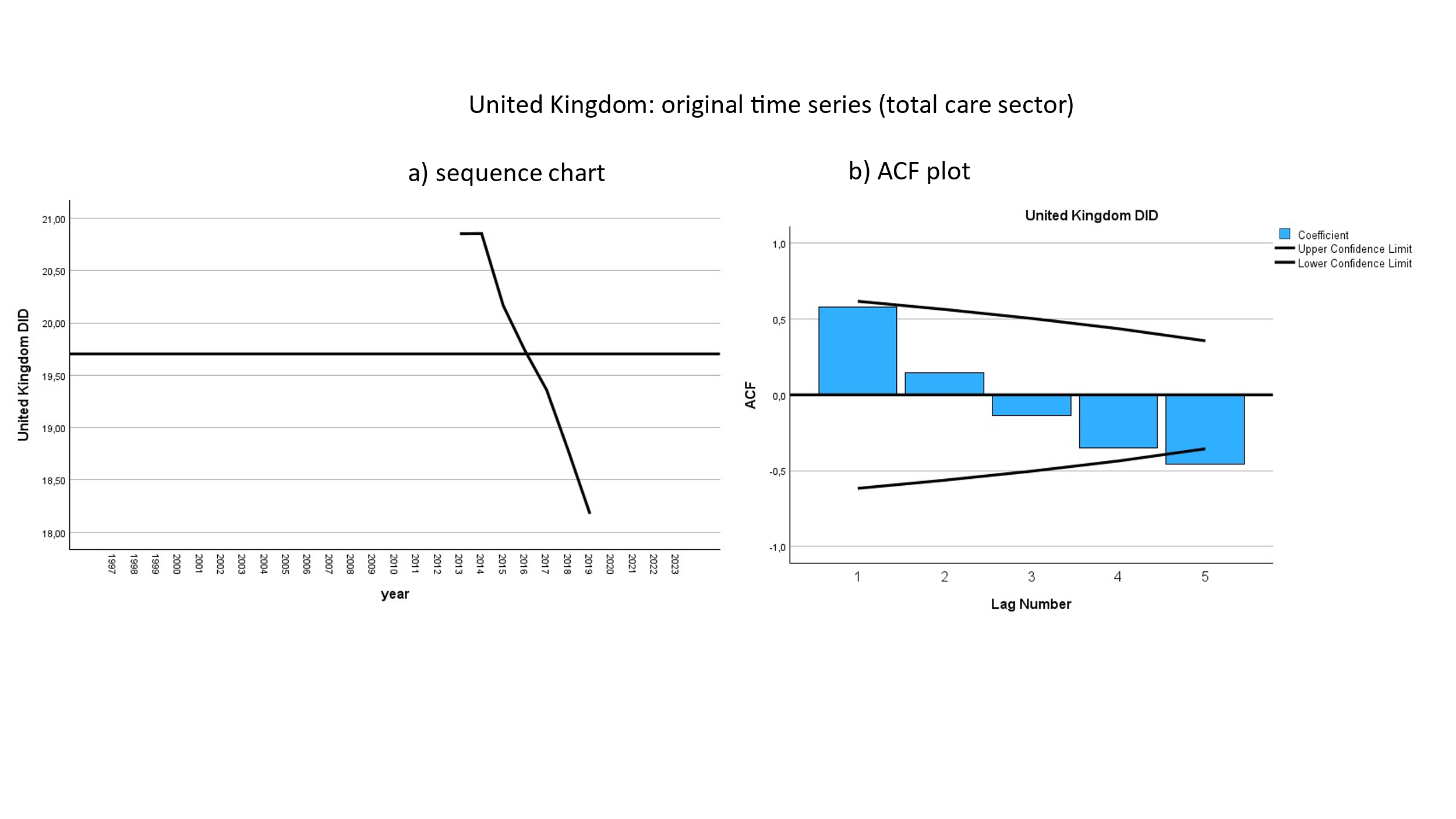


***Fig. S30:*** *Differentiated time series of ATC class J01 for Austria. In a) the sequence chart of consumption in DID is shown, while b) displays the ACF and c) the PACF plot of the autocorrelation. Stationarity can be seen in a roughly stable trend in both the sequence chart and the autocorrelation plots.*


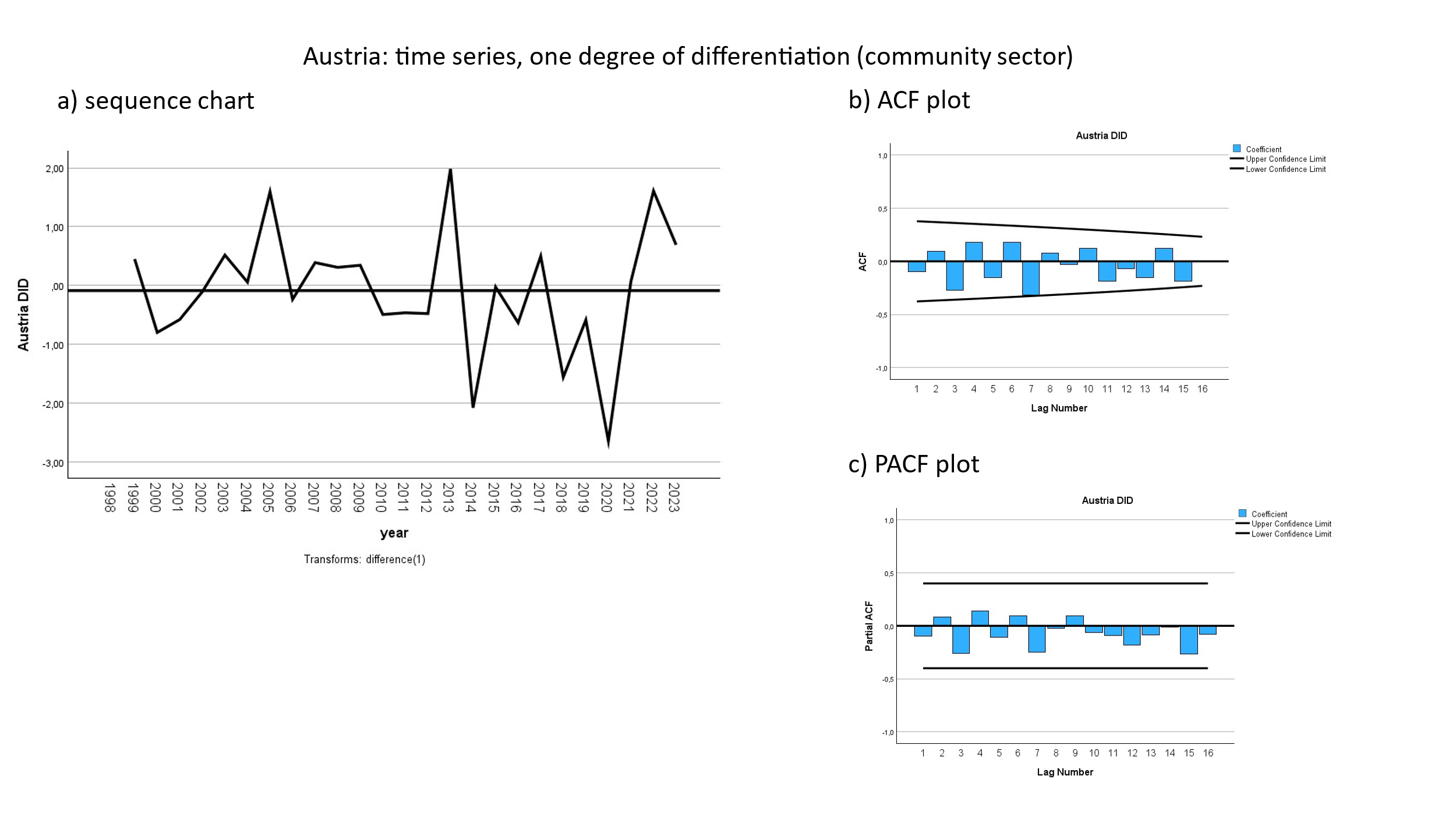


***Fig. S31:*** *Differentiated time series of ATC class J01 for Belgium. In a) the sequence chart of consumption in DID is shown, while b) displays the ACF and c) the PACF plot of the autocorrelation. Stationarity can be seen in a roughly stable trend in both the sequence chart and the autocorrelation plots.*


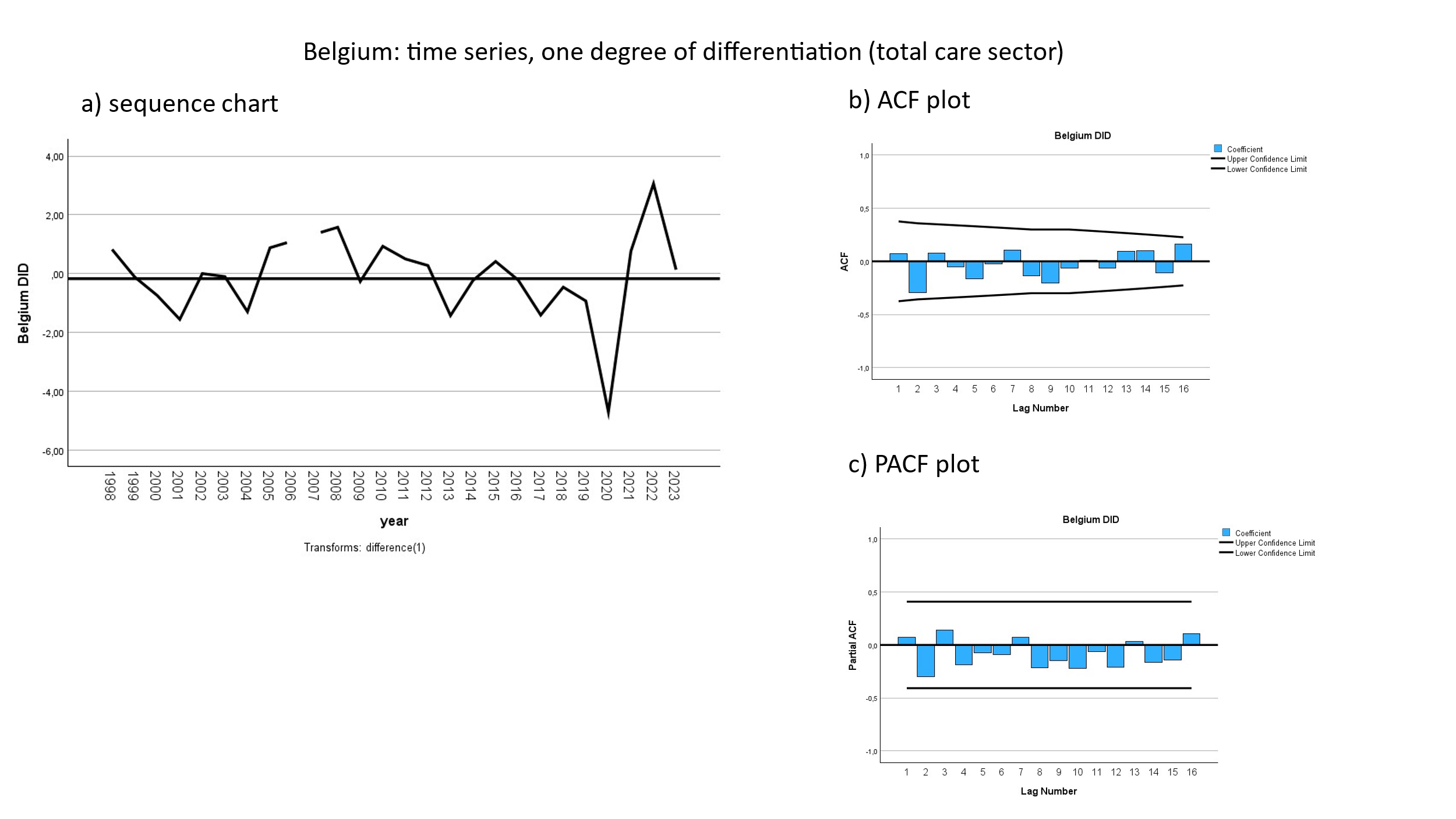


***Fig. S32:*** *Differentiated time series of ATC class J01 for Bulgaria. In a) the sequence chart of consumption in DID is shown, while b) displays the ACF and c) the PACF plot of the autocorrelation. Stationarity can be seen in a roughly stable trend in both the sequence chart and the autocorrelation plots.*


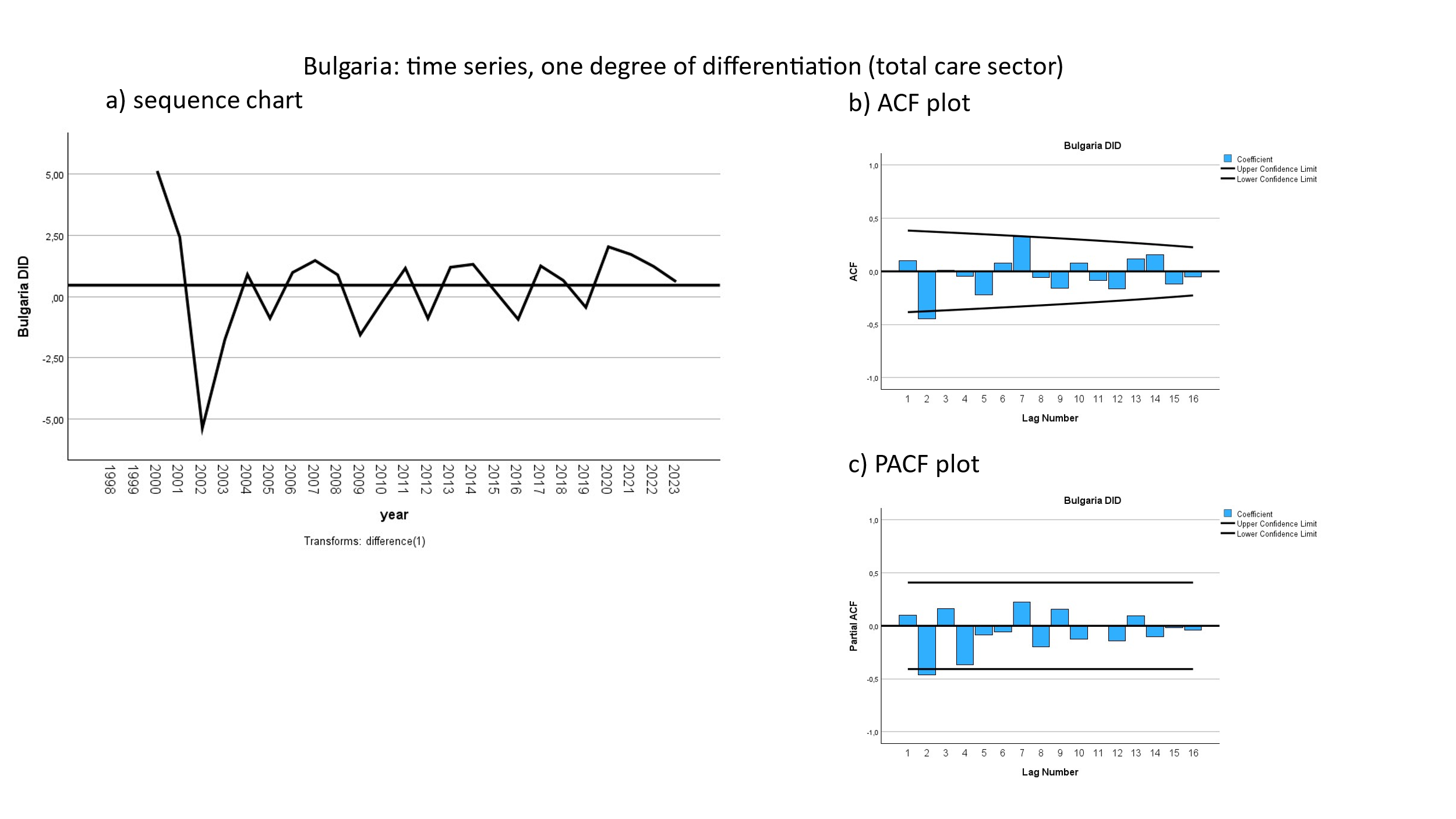


***Fig. S33:*** *Differentiated time series of ATC class J01 for Croatia. In a) the sequence chart of consumption in DID is shown, while b) displays the ACF and c) the PACF plot of the autocorrelation. Stationarity can be seen in a roughly stable trend in both the sequence chart and the autocorrelation plots.*


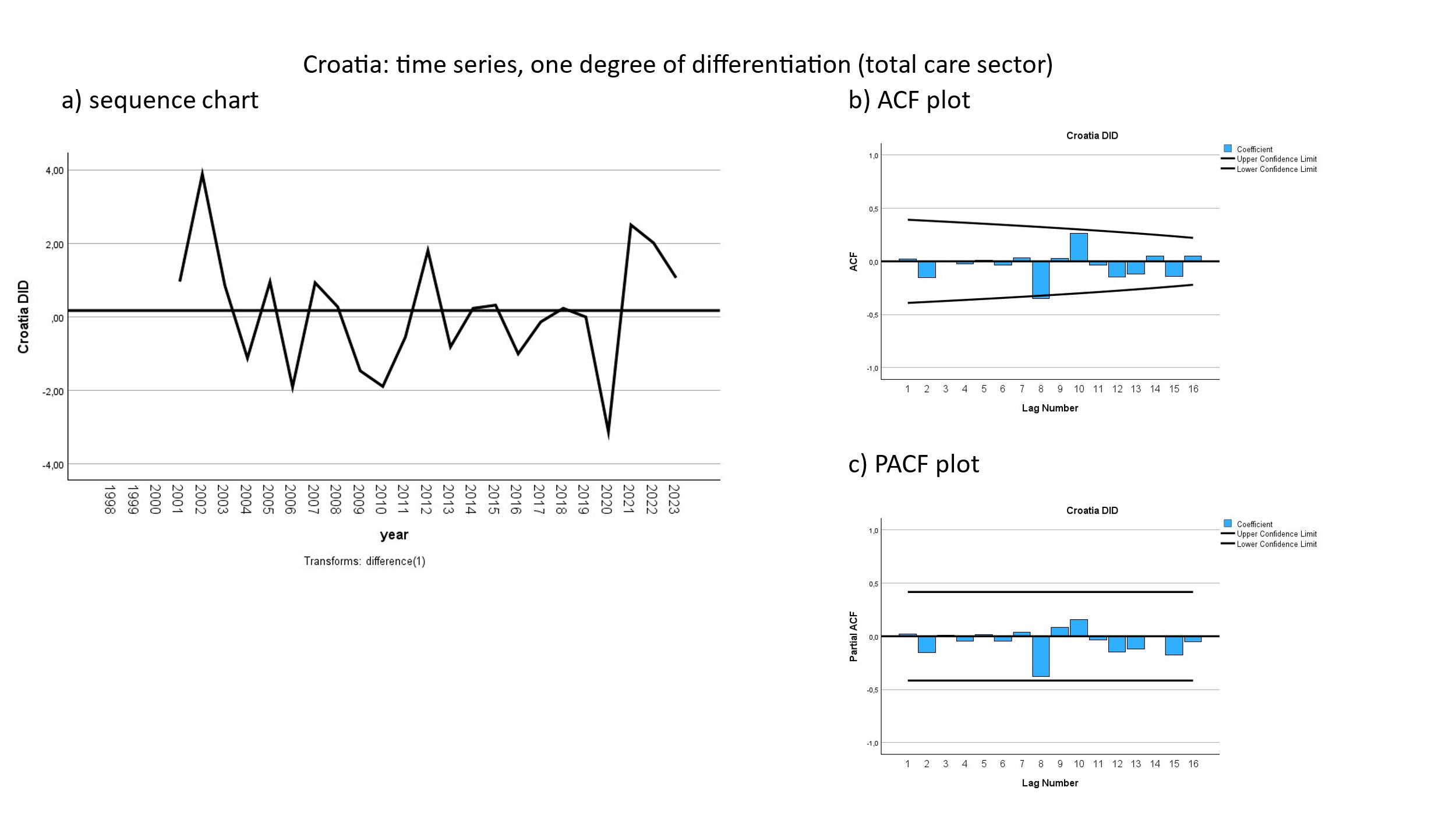


***Fig. S34:*** *Differentiated time series of ATC class J01 for Cyprus. In a) the sequence chart of consumption in DID is shown, while b) displays the ACF and c) the PACF plot of the autocorrelation. Stationarity can be seen in a roughly stable trend in both the sequence chart and the autocorrelation plots.*


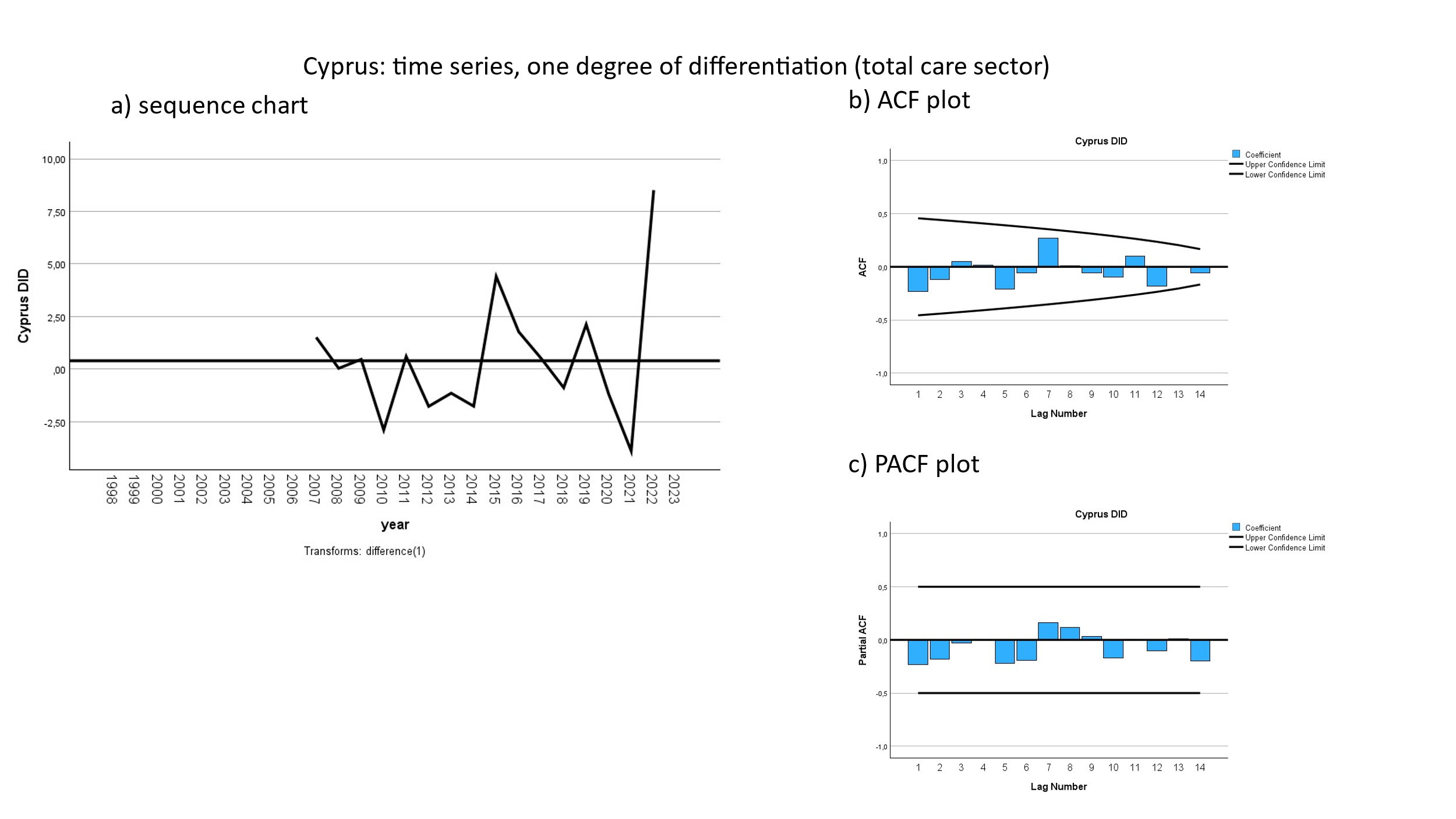


***Fig. S35:*** *Differentiated time series of ATC class J01 for Czechia. In a) the sequence chart of consumption in DID is shown, while b) displays the ACF and c) the PACF plot of the autocorrelation. Stationarity can be seen in a roughly stable trend in both the sequence chart and the autocorrelation plots.*


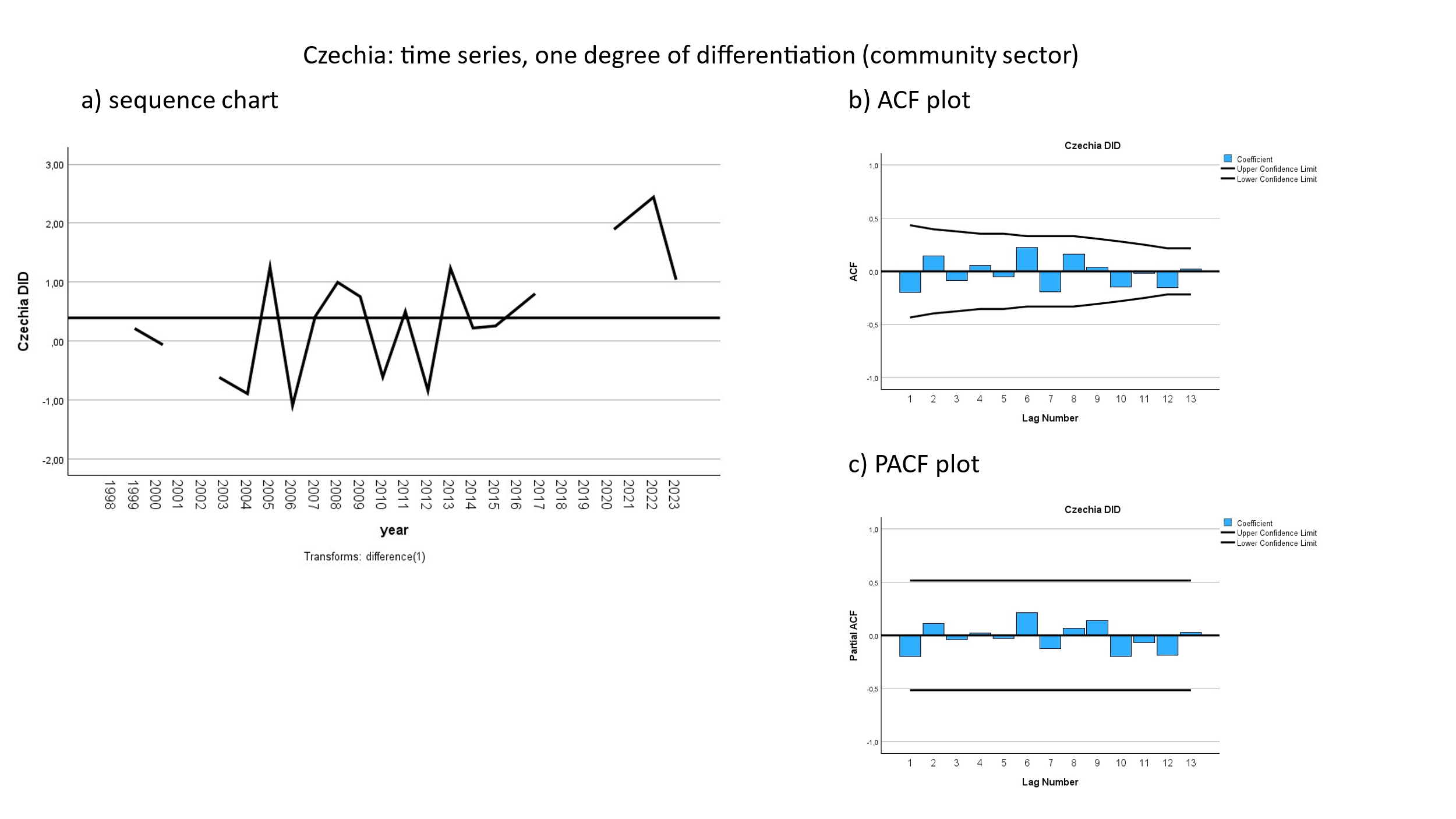


***Fig. S36:*** *Differentiated time series of ATC class J01 for Denmark. In a) the sequence chart of consumption in DID is shown, while b) displays the ACF and c) the PACF plot of the autocorrelation. Stationarity can be seen in a roughly stable trend in both the sequence chart and the autocorrelation plots.*


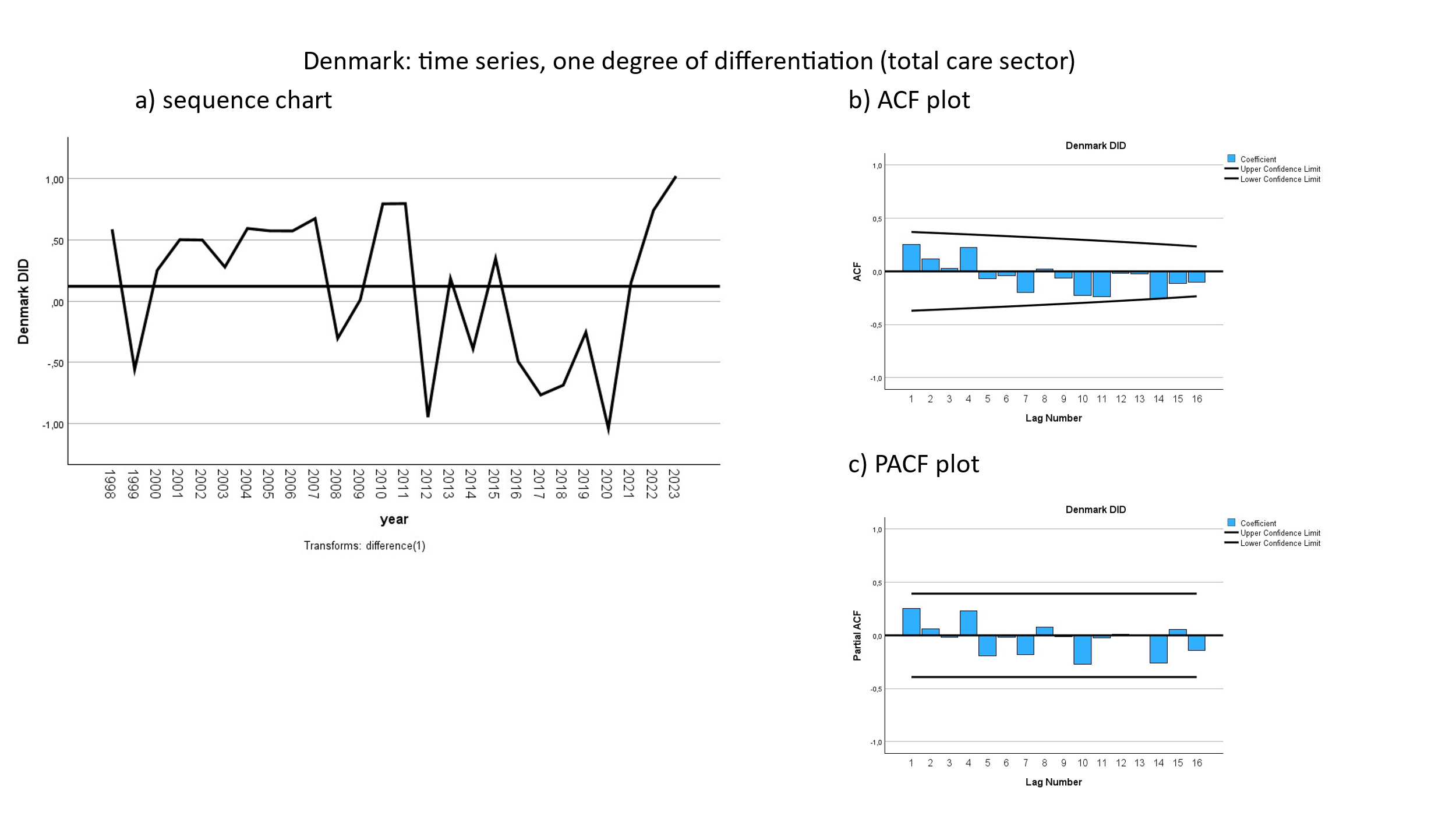


***Fig. S37:*** *Differentiated time series of ATC class J01 for Estonia. In a) the sequence chart of consumption in DID is shown, while b) displays the ACF and c) the PACF plot of the autocorrelation. Stationarity can be seen in a roughly stable trend in both the sequence chart and the autocorrelation plots.*


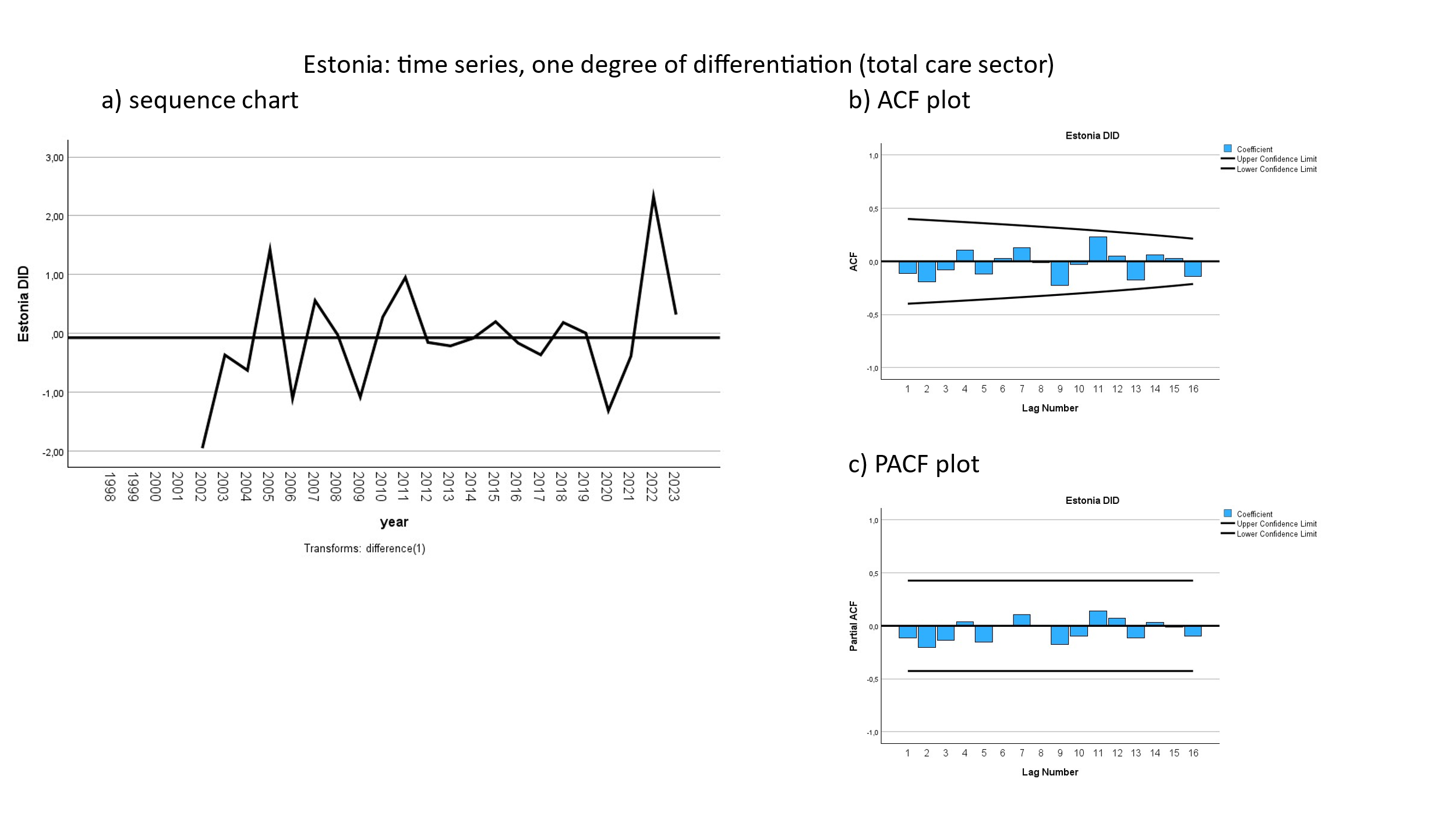


***Fig. S38:*** *Differentiated time series of ATC class J01 for Finland. In a) the sequence chart of consumption in DID is shown, while b) displays the ACF and c) the PACF plot of the autocorrelation. Stationarity can be seen in a roughly stable trend in both the sequence chart and the autocorrelation plots.*


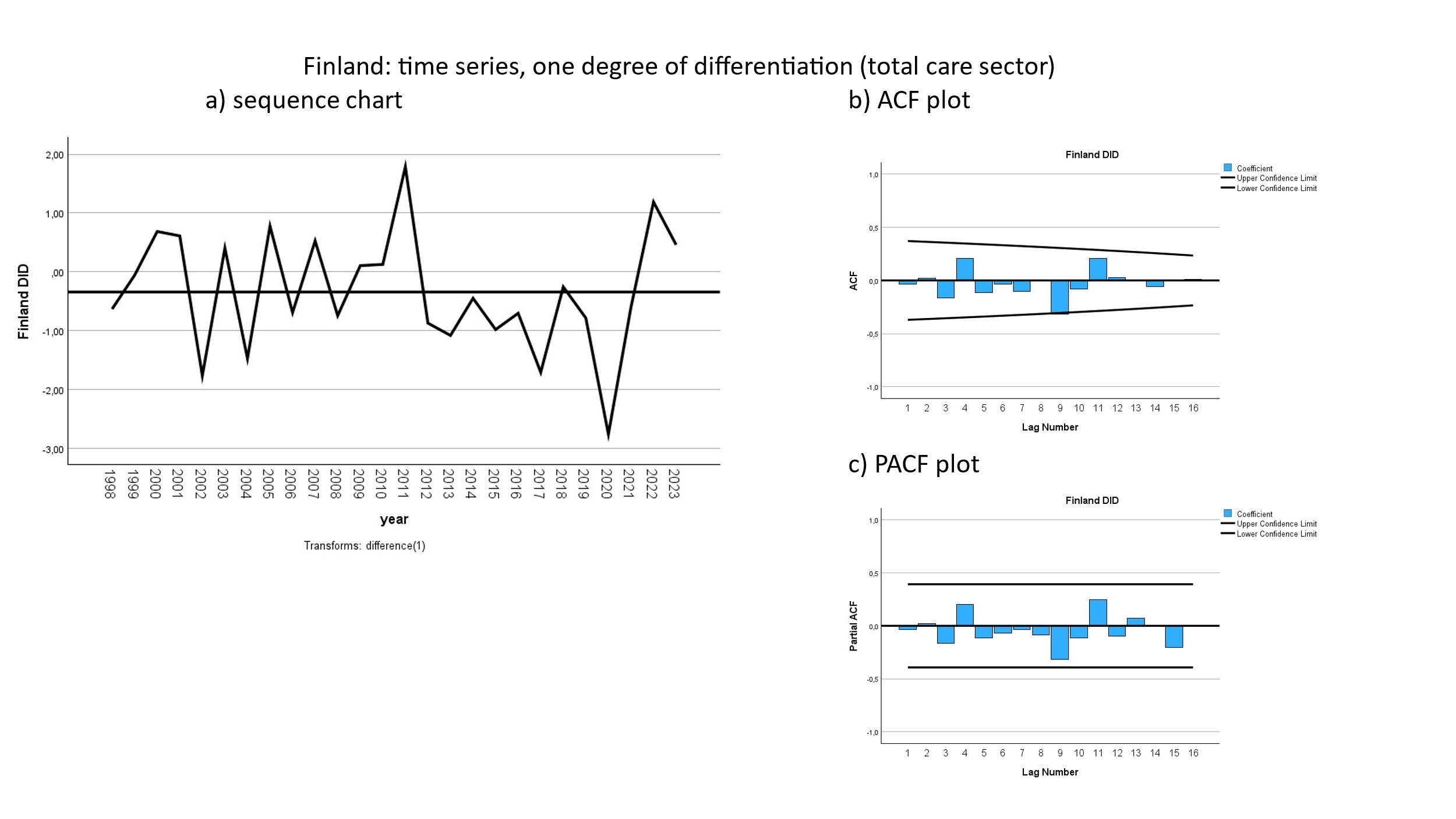


***Fig. S39:*** *Differentiated time series of ATC class J01 for France. In a) the sequence chart of consumption in DID is shown, while b) displays the ACF and c) the PACF plot of the autocorrelation. Stationarity can be seen in a roughly stable trend in both the sequence chart and the autocorrelation plots.*


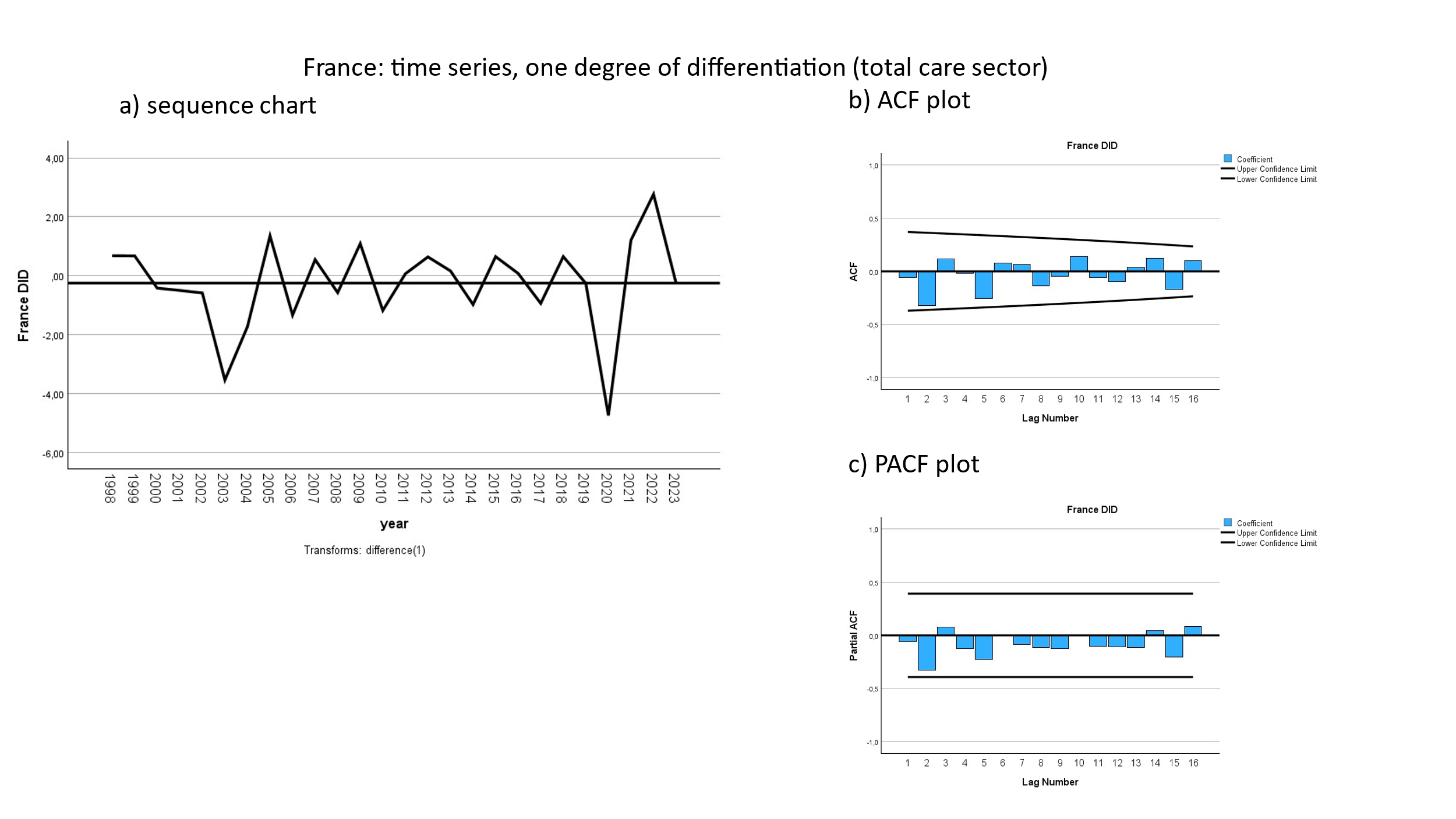


***Fig. S40:*** *Differentiated time series of ATC class J01 for Germany. In a) the sequence chart of consumption in DID is shown, while b) displays the ACF and c) the PACF plot of the autocorrelation. Stationarity can be seen in a roughly stable trend in both the sequence chart and the autocorrelation plots.*


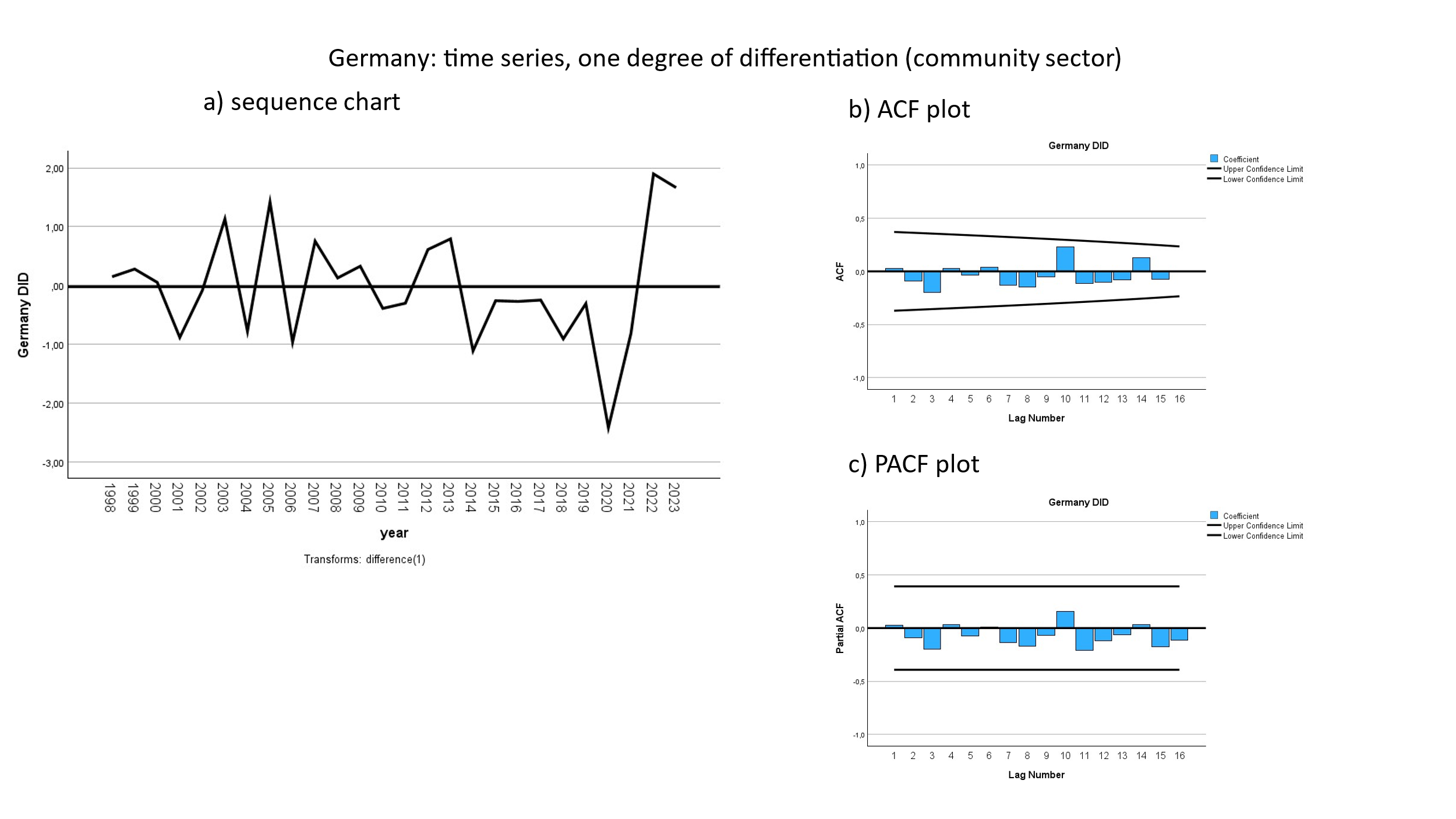


***Fig. S41:*** *Differentiated time series of ATC class J01 for Greece. In a) the sequence chart of consumption in DID is shown, while b) displays the ACF and c) the PACF plot of the autocorrelation. Stationarity can be seen in a roughly stable trend in both the sequence chart and the autocorrelation plots.*


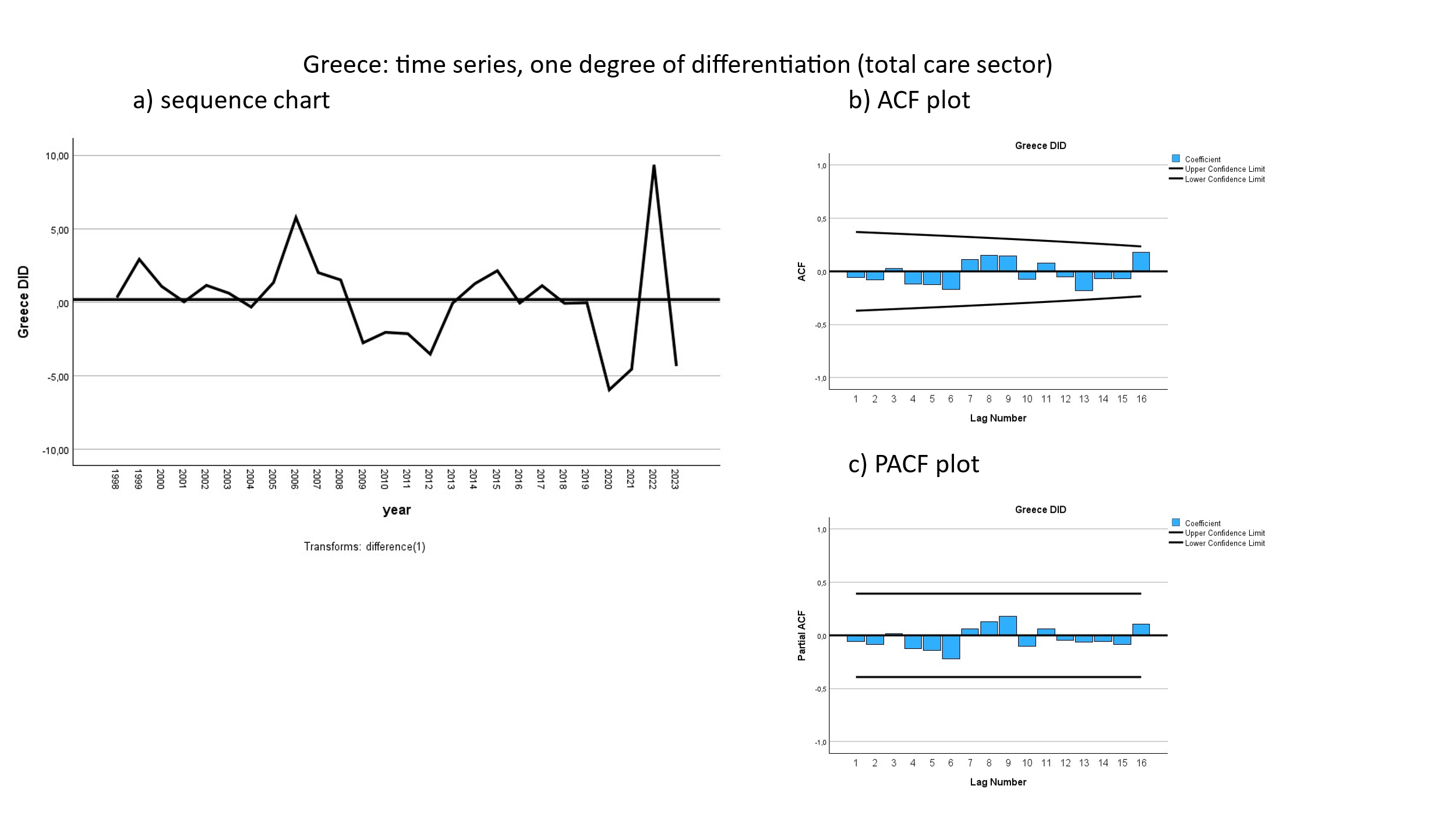


***Fig. S42:*** *Differentiated time series of ATC class J01 for Hungary. In a) the sequence chart of consumption in DID is shown, while b) displays the ACF and c) the PACF plot of the autocorrelation. Stationarity can be seen in a roughly stable trend in both the sequence chart and the autocorrelation plots.*


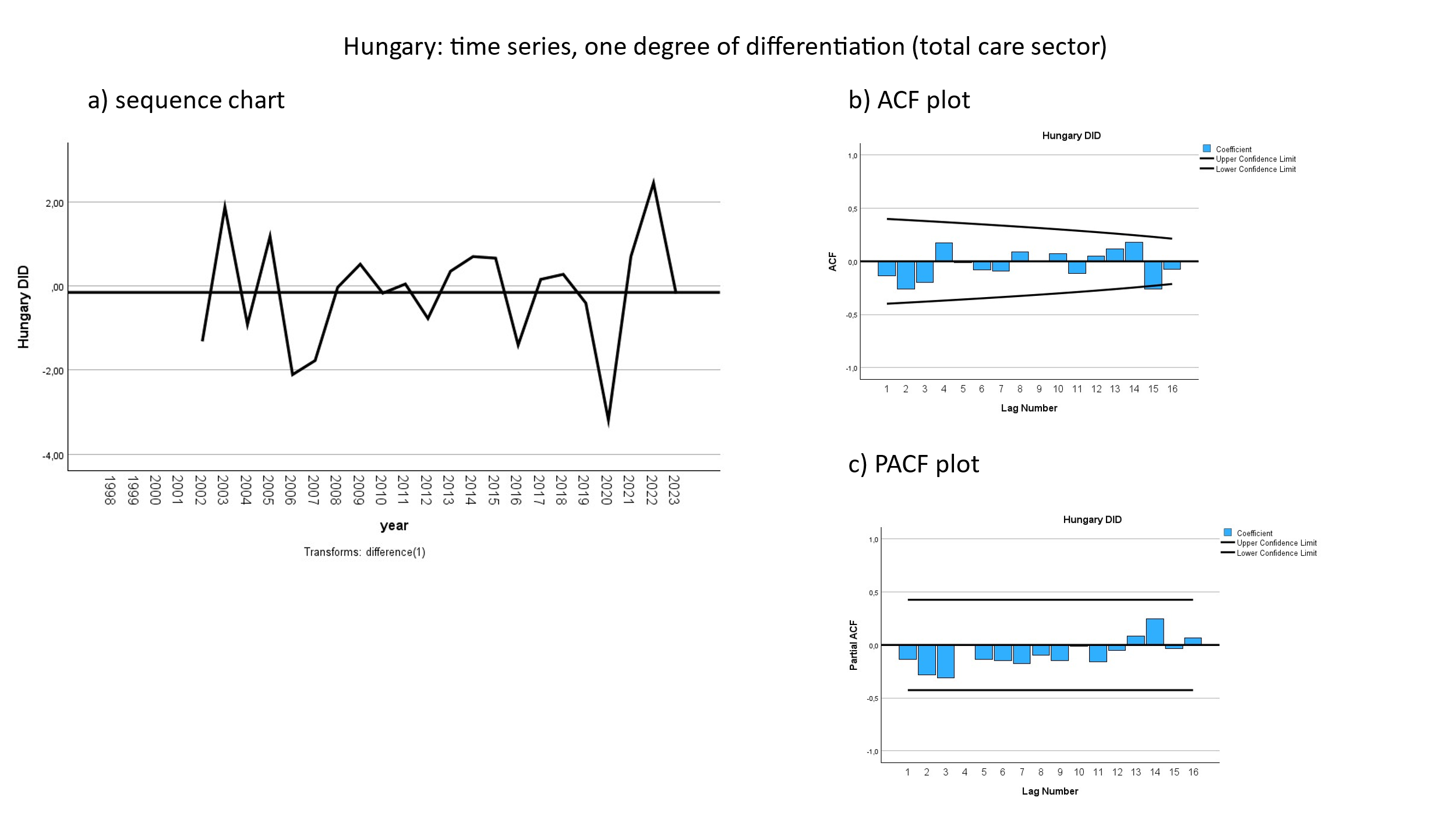


***Fig. S43:*** *Differentiated time series of ATC class J01 for Ireland. In a) the sequence chart of consumption in DID is shown, while b) displays the ACF and c) the PACF plot of the autocorrelation. Stationarity can be seen in a roughly stable trend in both the sequence chart and the autocorrelation plots.*


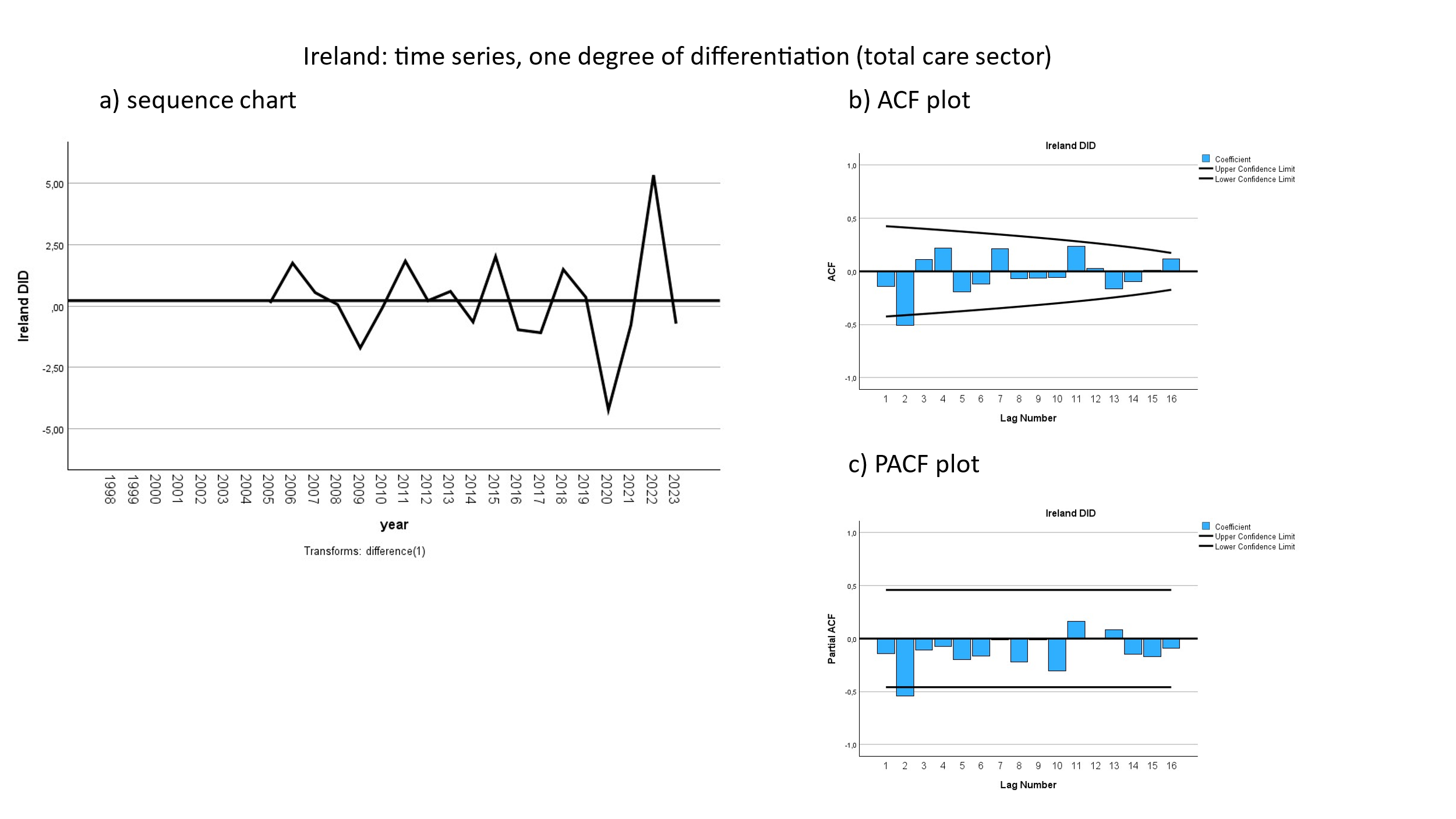


***Fig. S44:*** *Differentiated time series of ATC class J01 for Italy. In a) the sequence chart of consumption in DID is shown, while b) displays the ACF and c) the PACF plot of the autocorrelation. Stationarity can be seen in a roughly stable trend in both the sequence chart and the autocorrelation plots.*


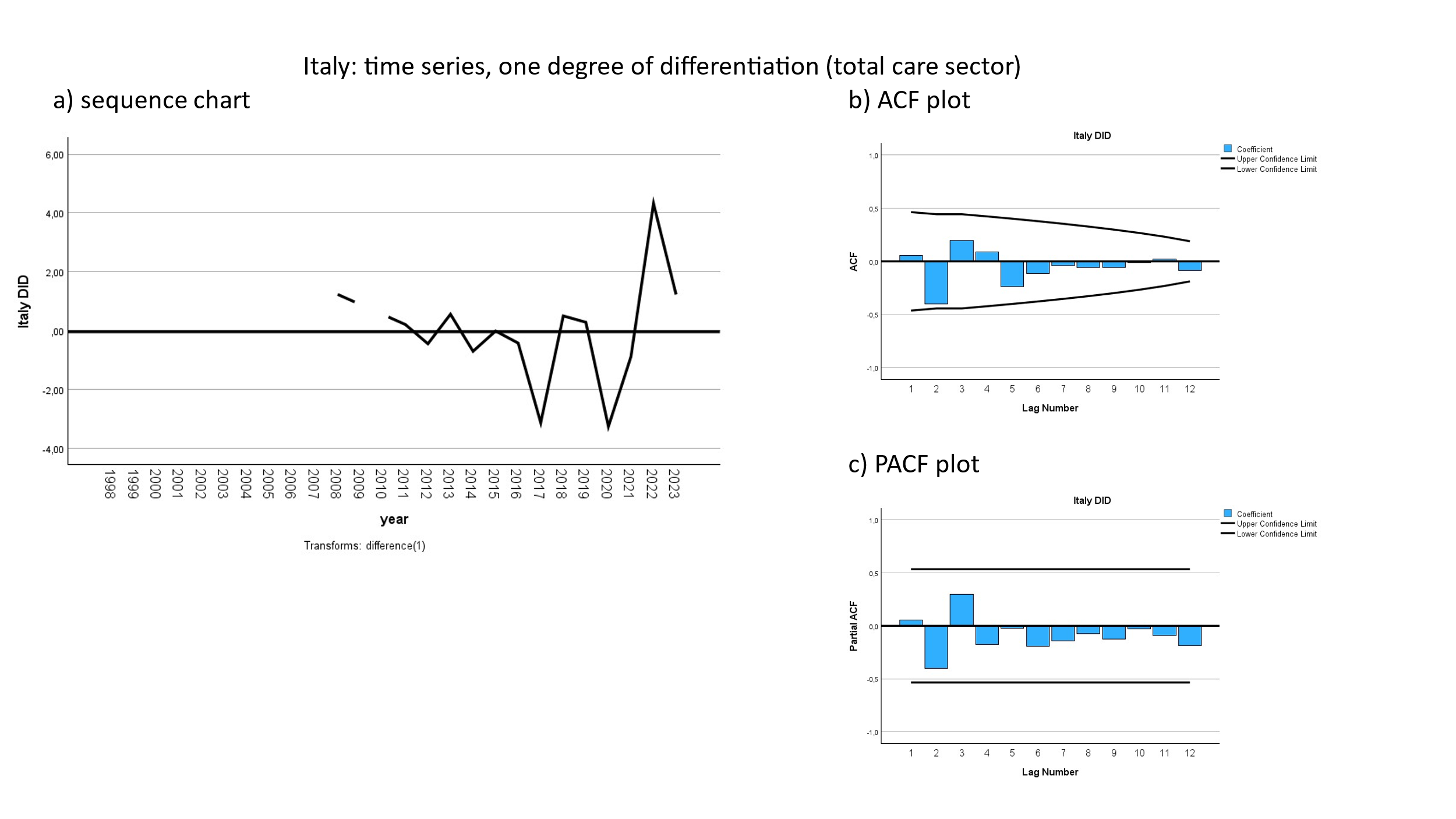


***Fig. S45:*** *Differentiated time series of ATC class J01 for Iceland. In a) the sequence chart of consumption in DID is shown, while b) displays the ACF and c) the PACF plot of the autocorrelation. Stationarity can be seen in a roughly stable trend in both the sequence chart and the autocorrelation plots.*


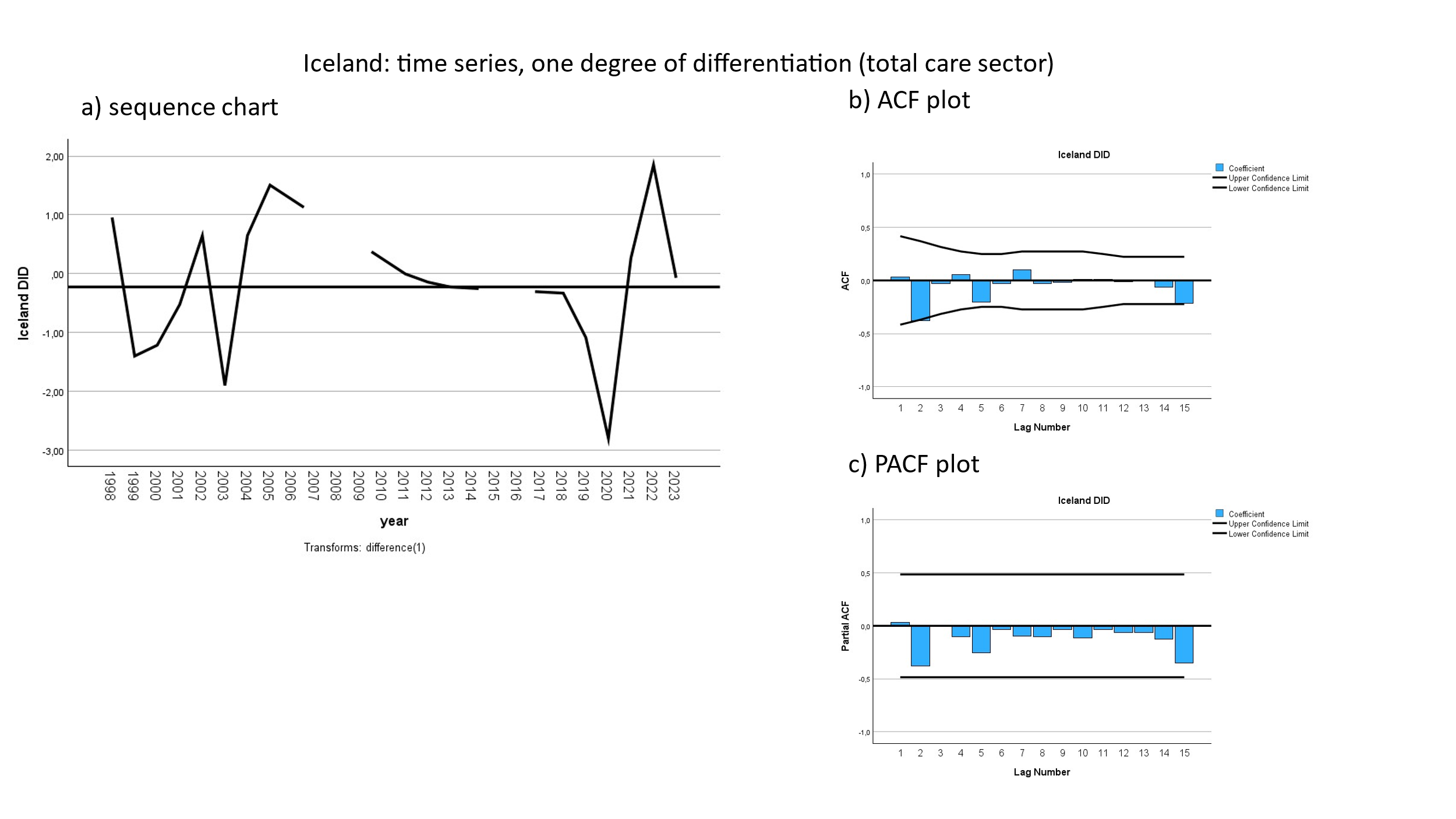


***Fig. S46:*** *Differentiated time series of ATC class J01 for Lithuania. In a) the sequence chart of consumption in DID is shown, while b) displays the ACF and c) the PACF plot of the autocorrelation. Stationarity can be seen in a roughly stable trend in both the sequence chart and the autocorrelation plots.*


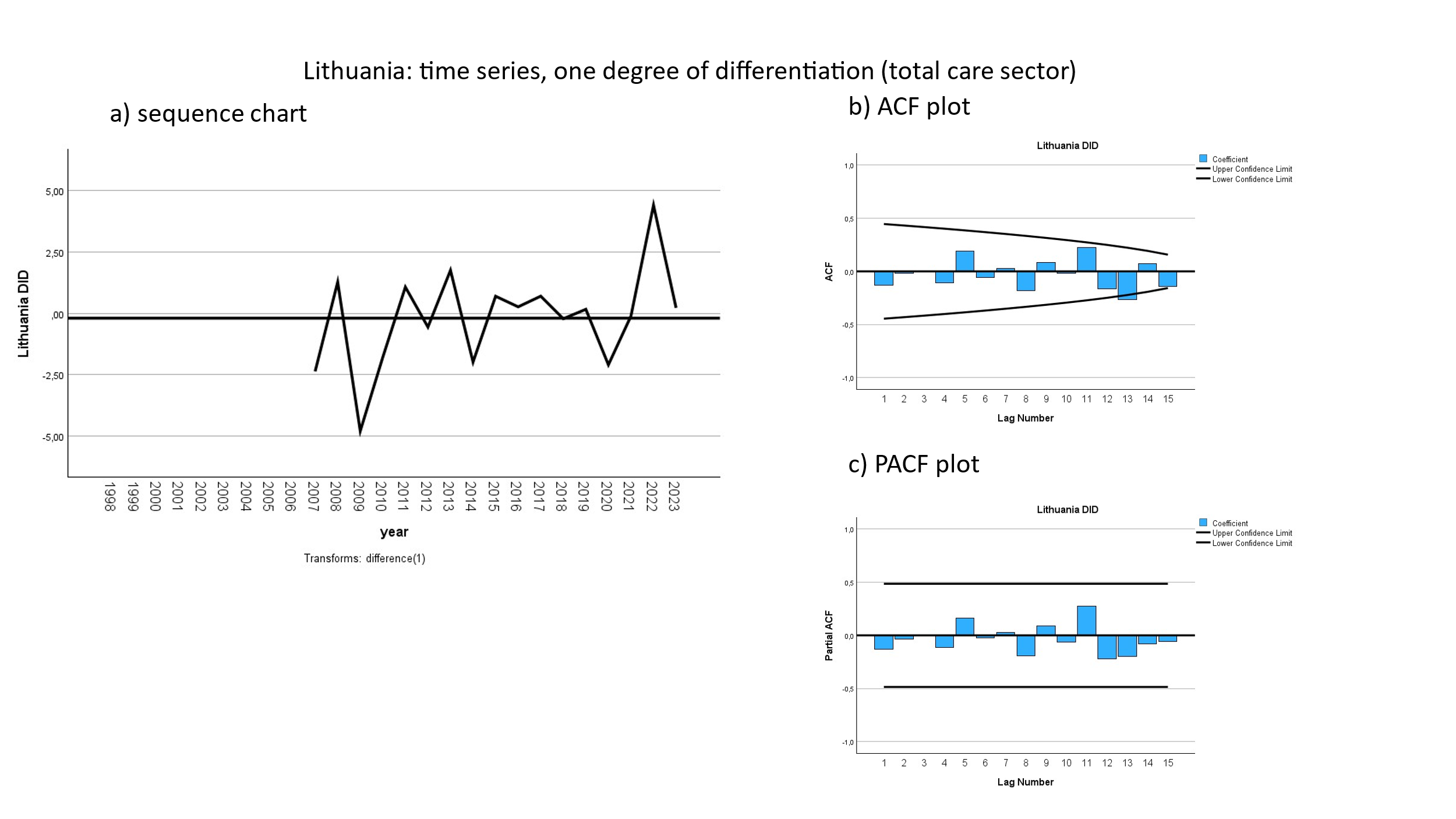


***Fig. S47:*** *Differentiated time series of ATC class J01 for Luxembourg. In a) the sequence chart of consumption in DID is shown, while b) displays the ACF and c) the PACF plot of the autocorrelation. Stationarity can be seen in a roughly stable trend in both the sequence chart and the autocorrelation plots.*


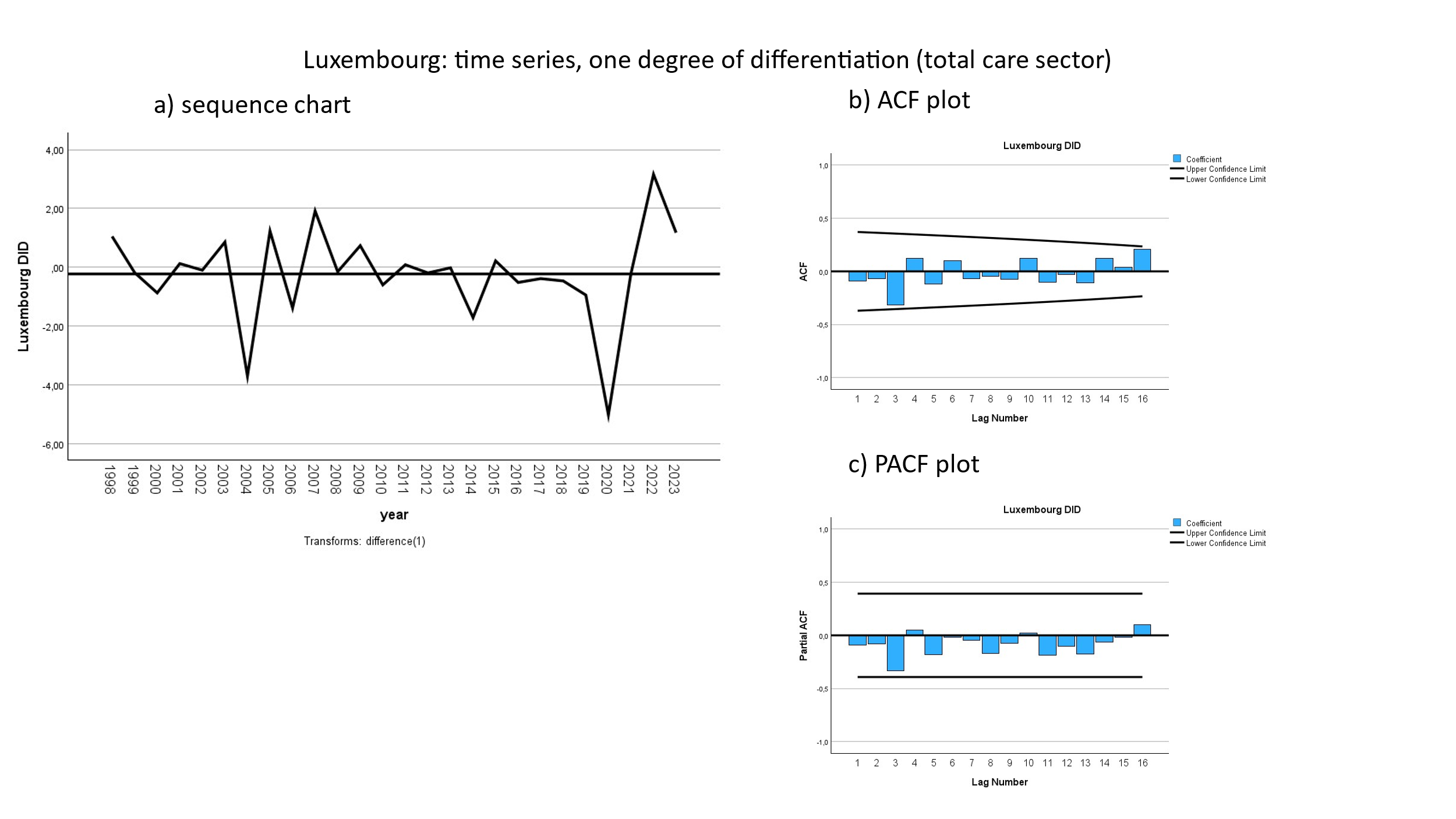


***Fig. S48:*** *Differentiated time series of ATC class J01 for Latvia. In a) the sequence chart of consumption in DID is shown, while b) displays the ACF and c) the PACF plot of the autocorrelation. Stationarity can be seen in a roughly stable trend in both the sequence chart and the autocorrelation plots.*


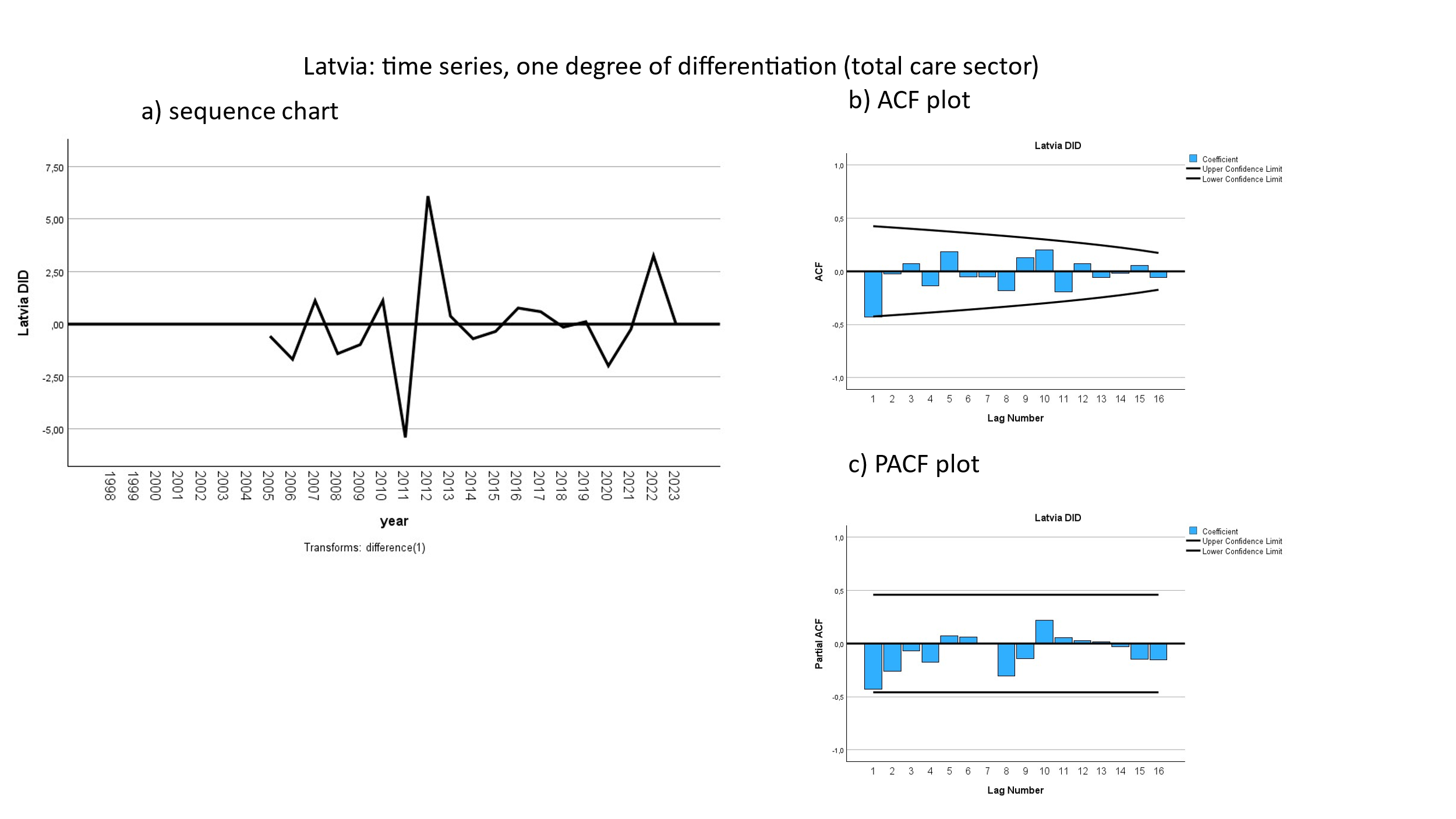


***Fig. S49:*** *Differentiated time series of ATC class J01 for Malta. In a) the sequence chart of consumption in DID is shown, while b) displays the ACF and c) the PACF plot of the autocorrelation. Stationarity can be seen in a roughly stable trend in both the sequence chart and the autocorrelation plots.*


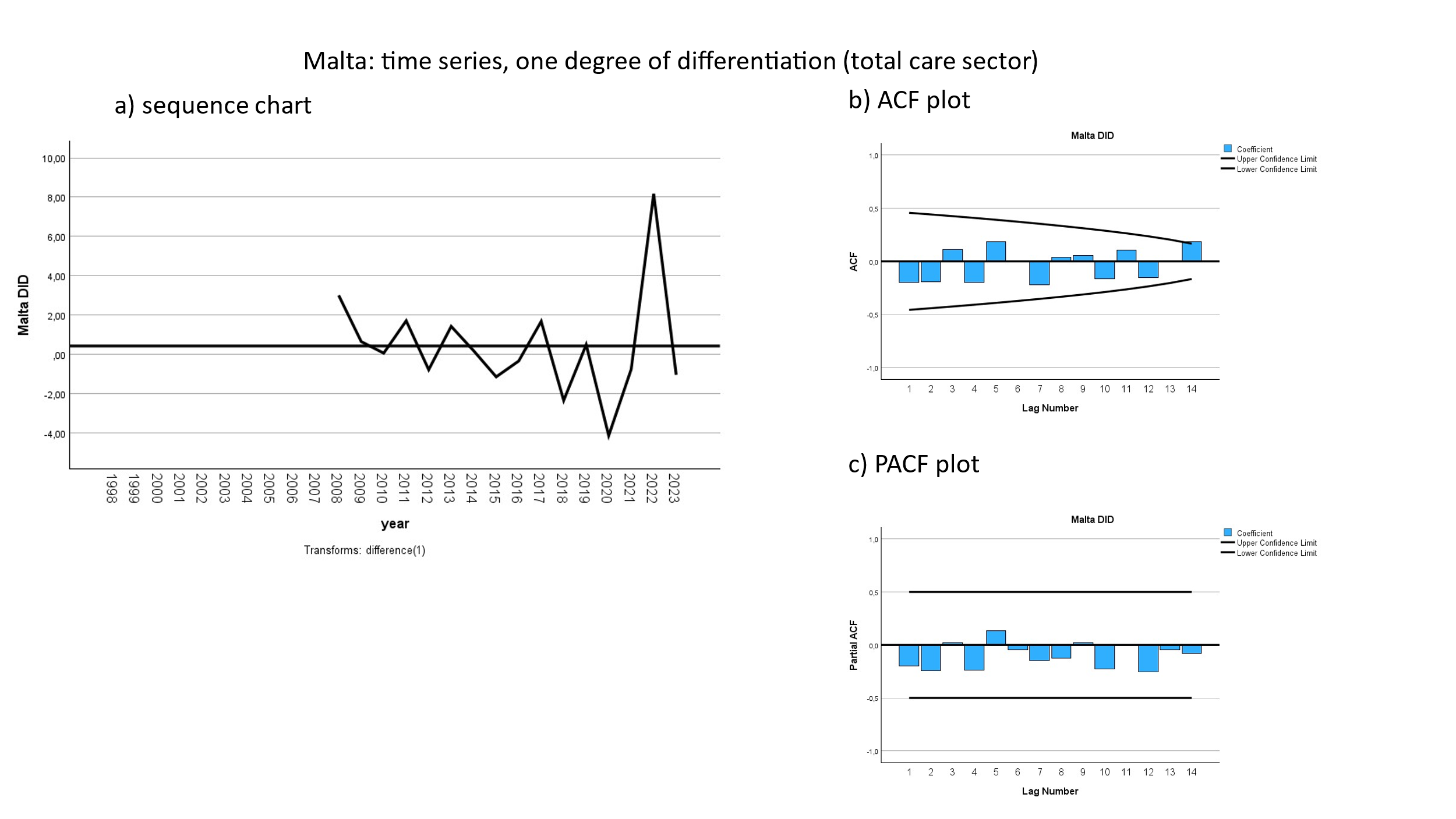


***Fig. S50:*** *Differentiated time series of ATC class J01 for the Netherlands. In a) the sequence chart of consumption in DID is shown, while b) displays the ACF and c) the PACF plot of the autocorrelation. Stationarity can be seen in a roughly stable trend in both the sequence chart and the autocorrelation plots.*


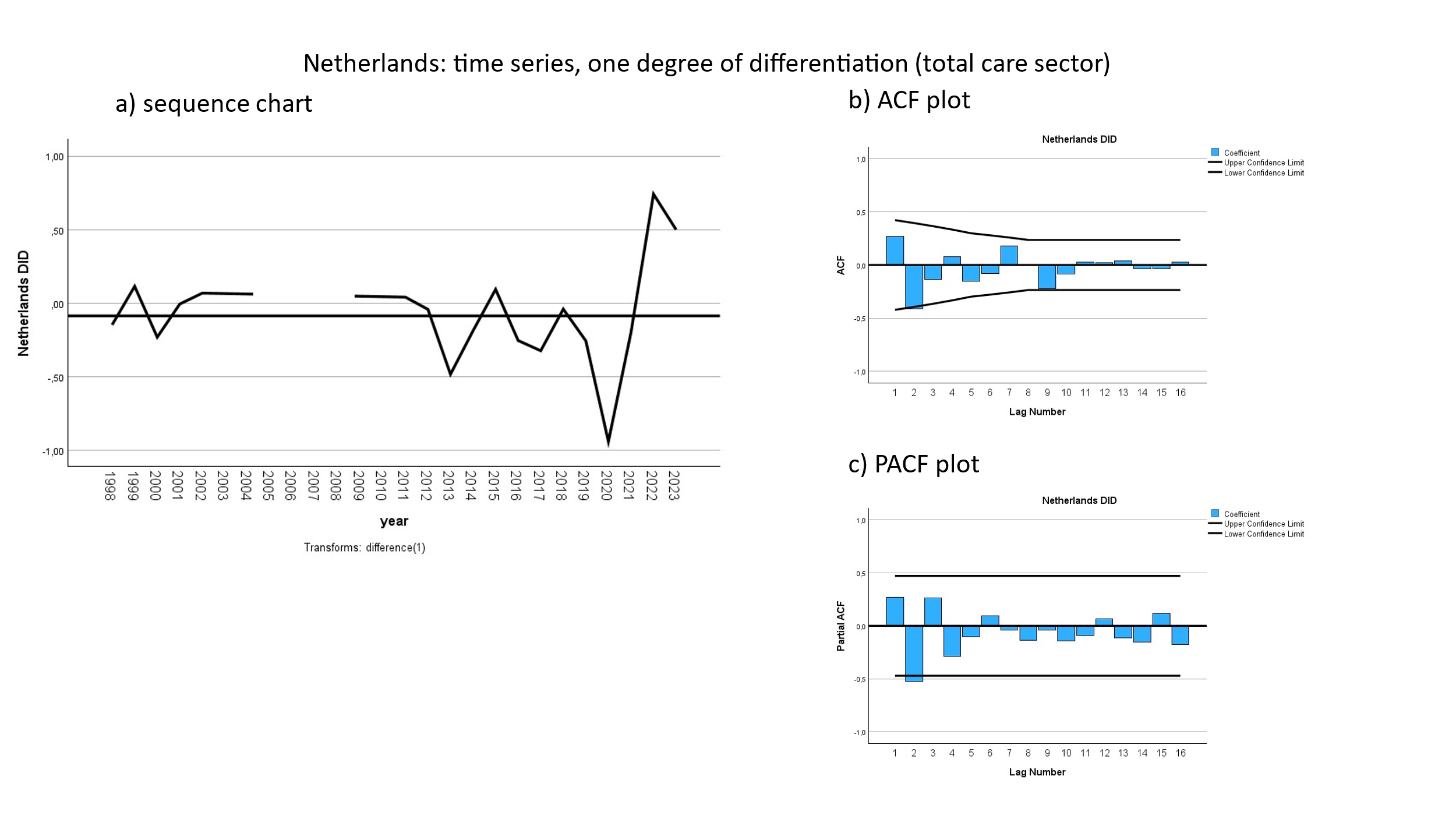


***Fig. S51:*** *Differentiated time series of ATC class J01 for Norway. In a) the sequence chart of consumption in DID is shown, while b) displays the ACF and c) the PACF plot of the autocorrelation. Stationarity can be seen in a roughly stable trend in both the sequence chart and the autocorrelation plots.*


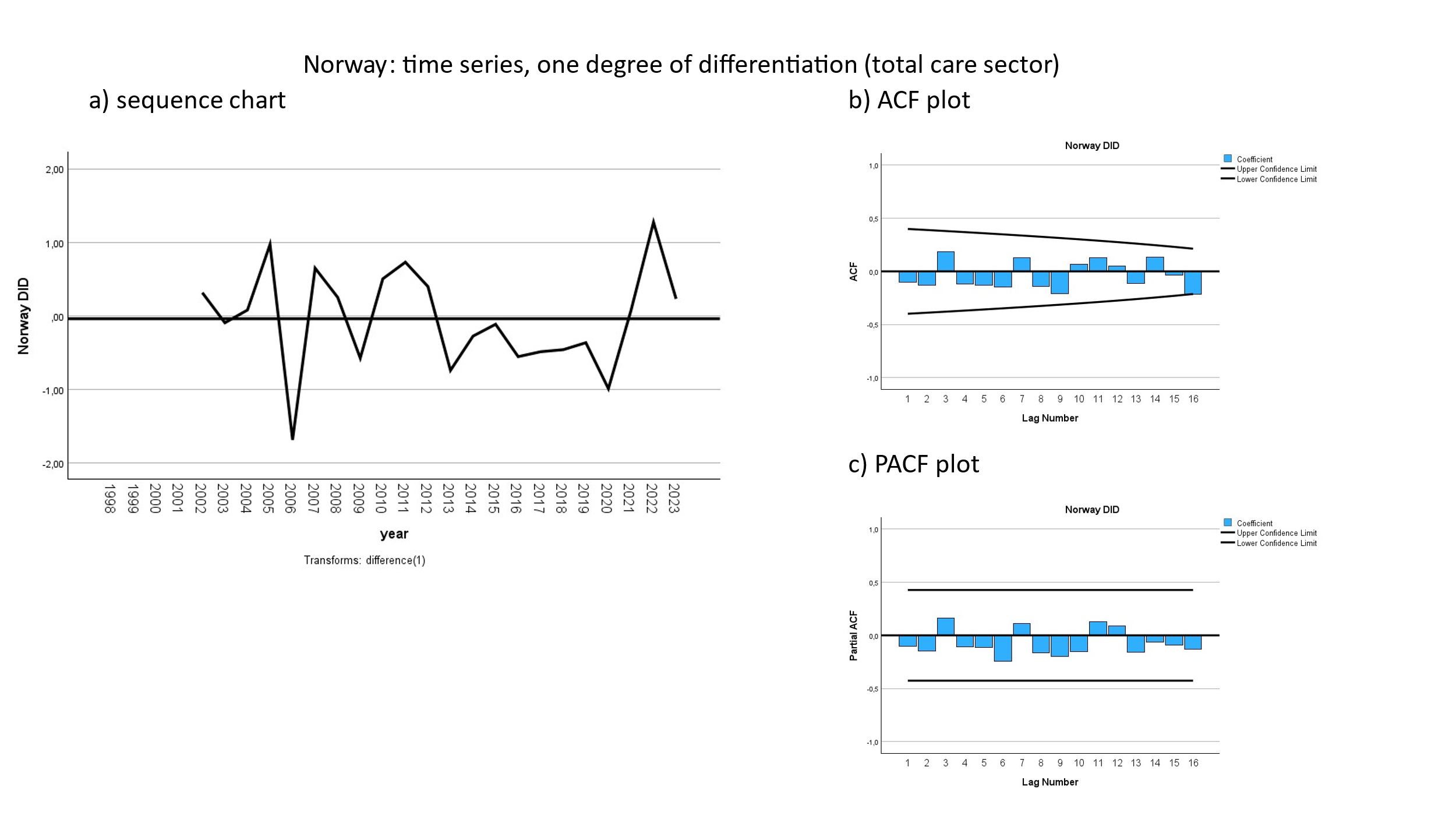


***Fig. S52:*** *Differentiated time series of ATC class J01 for Poland. In a) the sequence chart of consumption in DID is shown, while b) displays the ACF and c) the PACF plot of the autocorrelation. Stationarity can be seen in a roughly stable trend in both the sequence chart and the autocorrelation plots.*


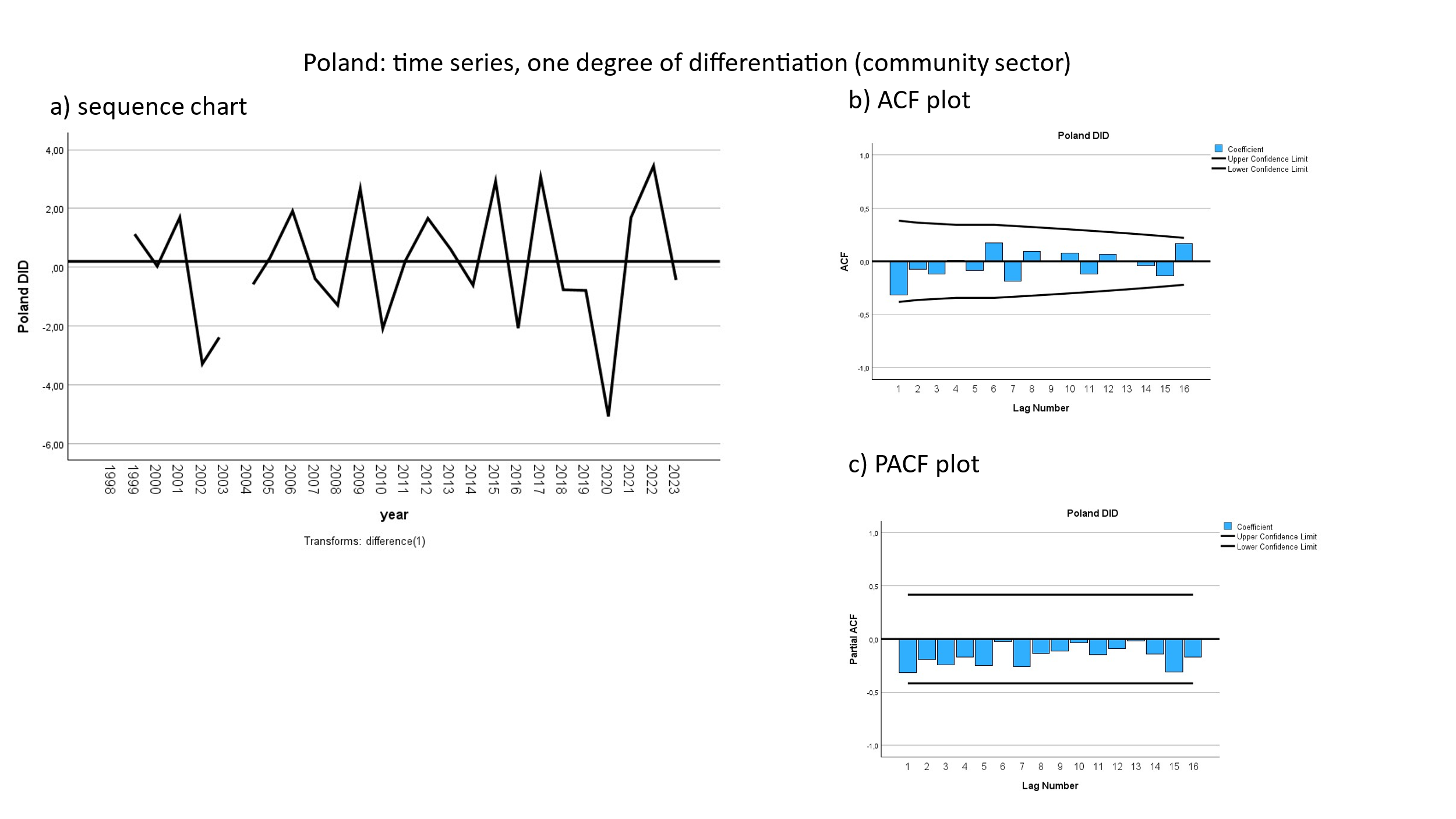


***Fig. S53:*** *Differentiated time series of ATC class J01 for Portugal. In a) the sequence chart of consumption in DID is shown, while b) displays the ACF and c) the PACF plot of the autocorrelation. Stationarity can be seen in a roughly stable trend in both the sequence chart and the autocorrelation plots.*


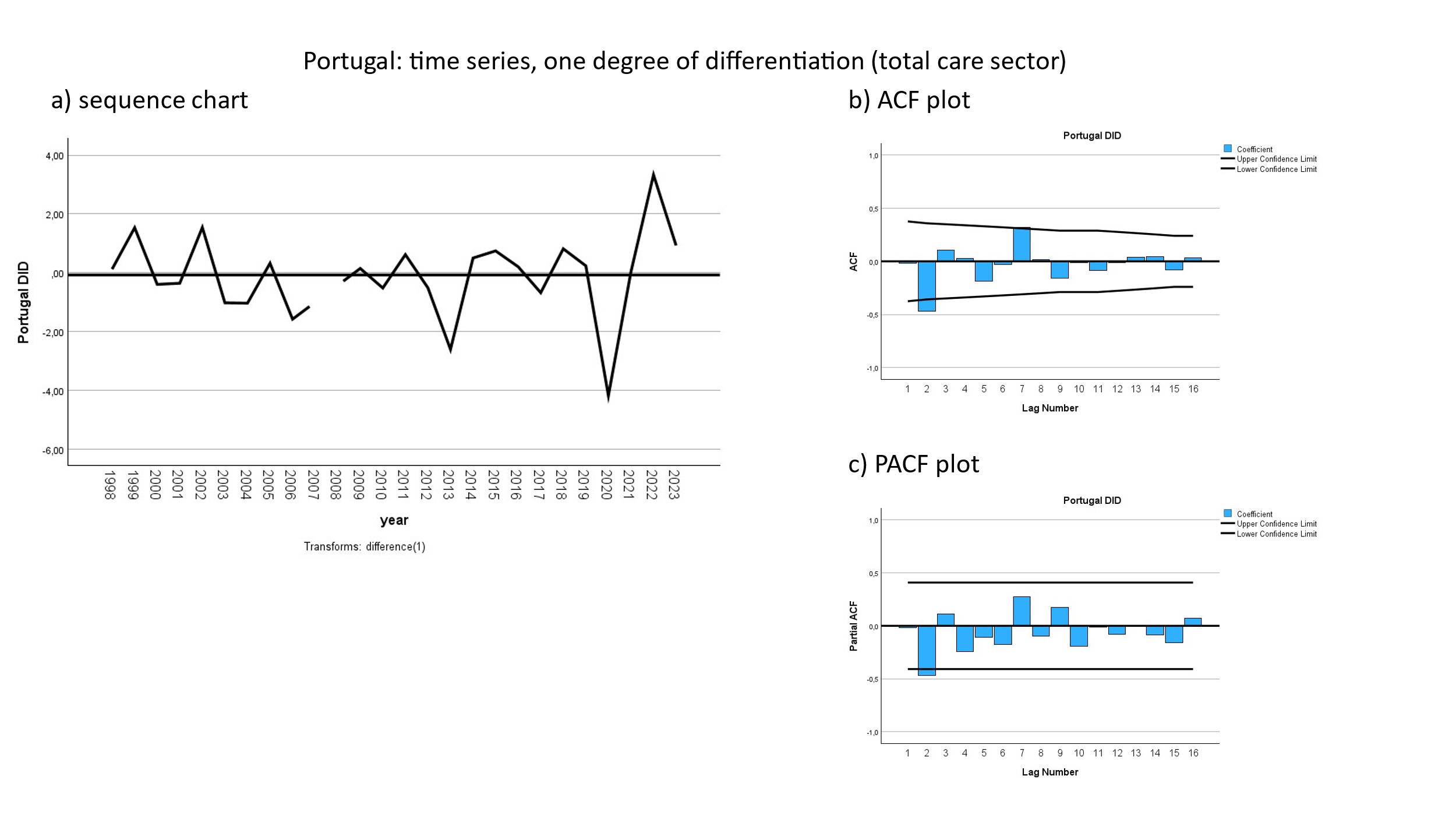


***Fig. S54:*** *Differentiated time series of ATC class J01 for Romania. In a) the sequence chart of consumption in DID is shown, while b) displays the ACF and c) the PACF plot of the autocorrelation. Stationarity can be seen in a roughly stable trend in both the sequence chart and the autocorrelation plots.*


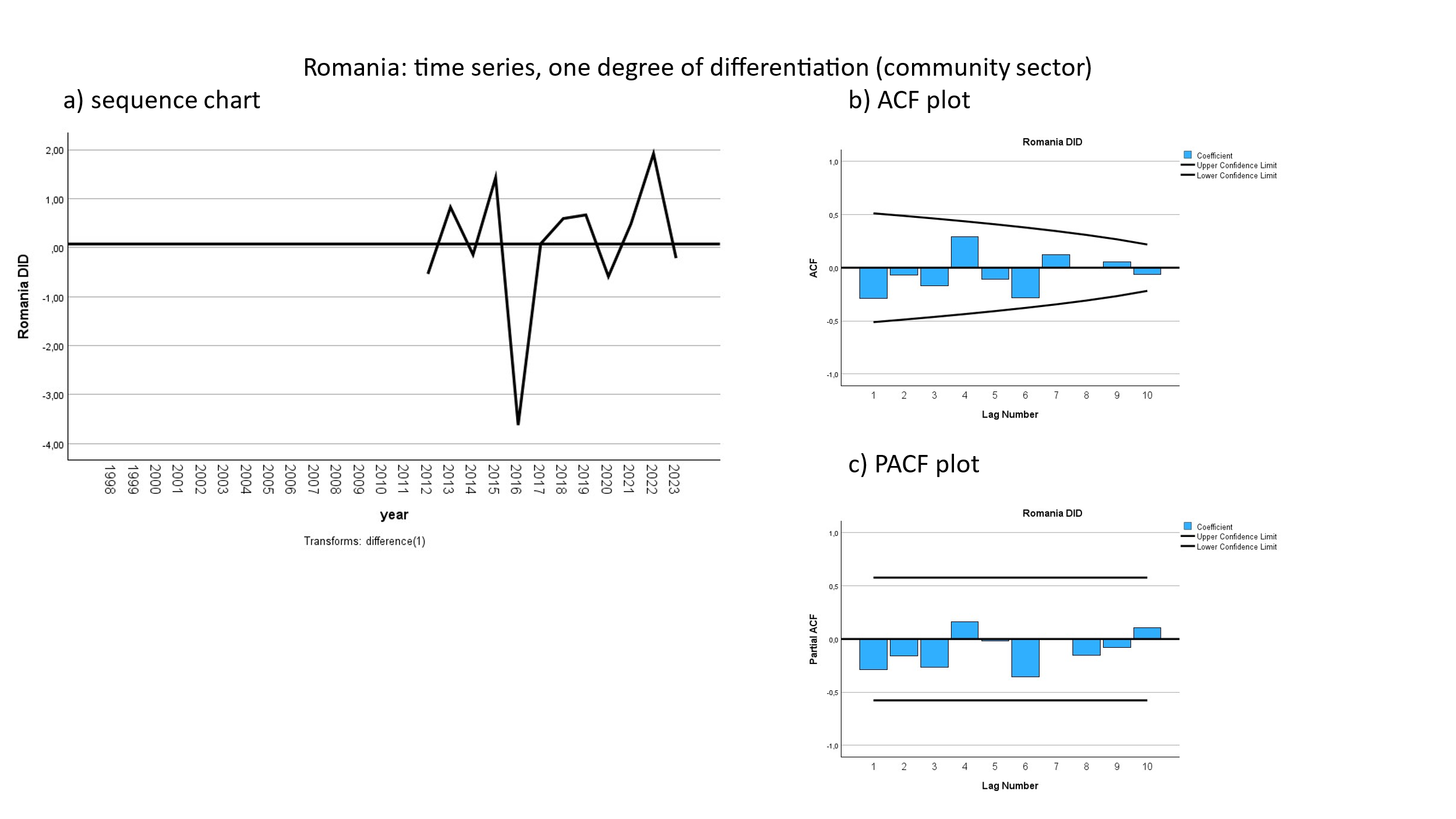


***Fig. S55:*** *Differentiated time series of ATC class J01 for Slovenia. In a) the sequence chart of consumption in DID is shown, while b) displays the ACF and c) the PACF plot of the autocorrelation. Stationarity can be seen in a roughly stable trend in both the sequence chart and the autocorrelation plots.*


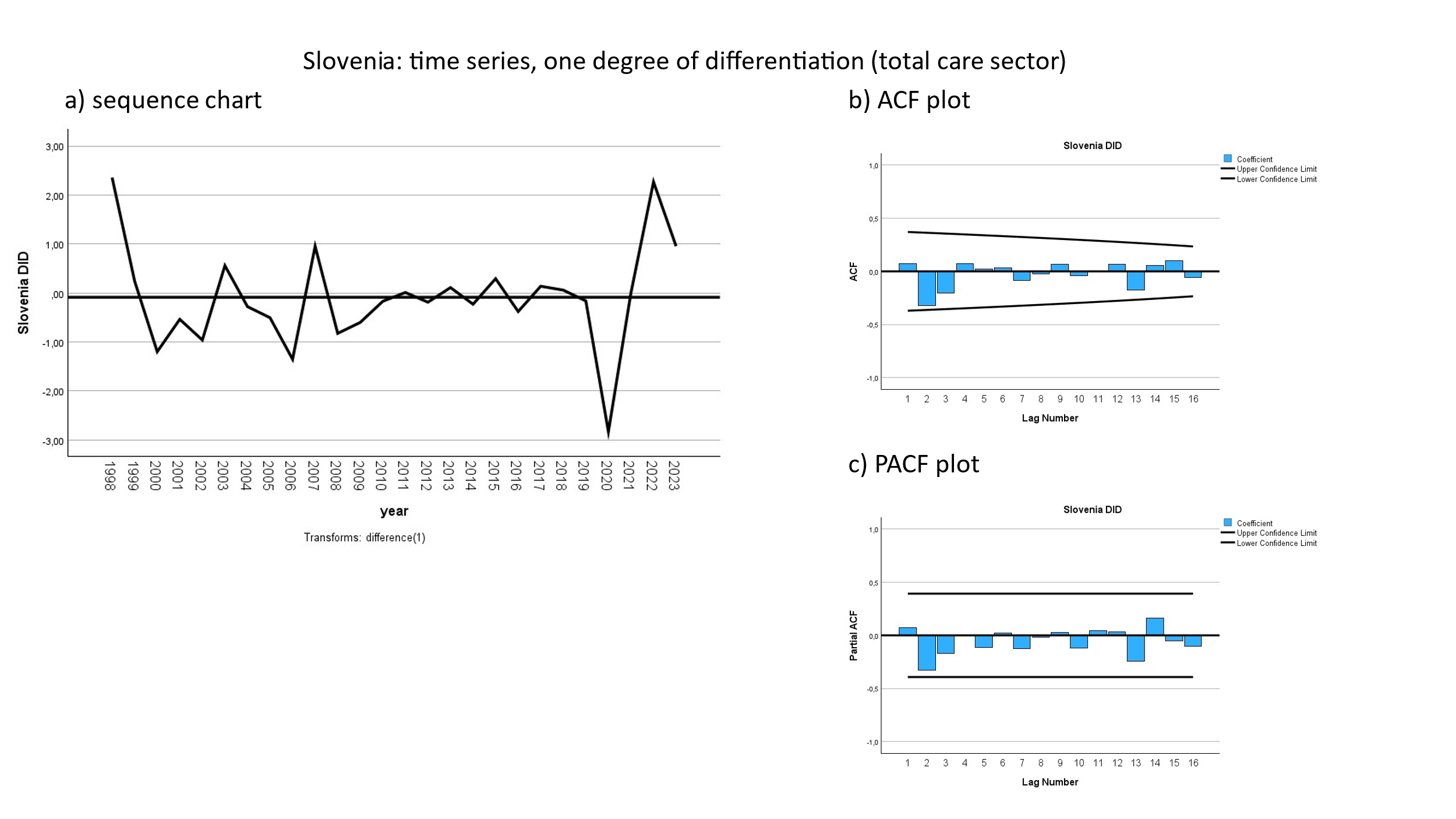


***Fig. S56:*** *Differentiated time series of ATC class J01 for Slovakia. In a) the sequence chart of consumption in DID is shown, while b) displays the ACF and c) the PACF plot of the autocorrelation. Stationarity can be seen in a roughly stable trend in both the sequence chart and the autocorrelation plots.*


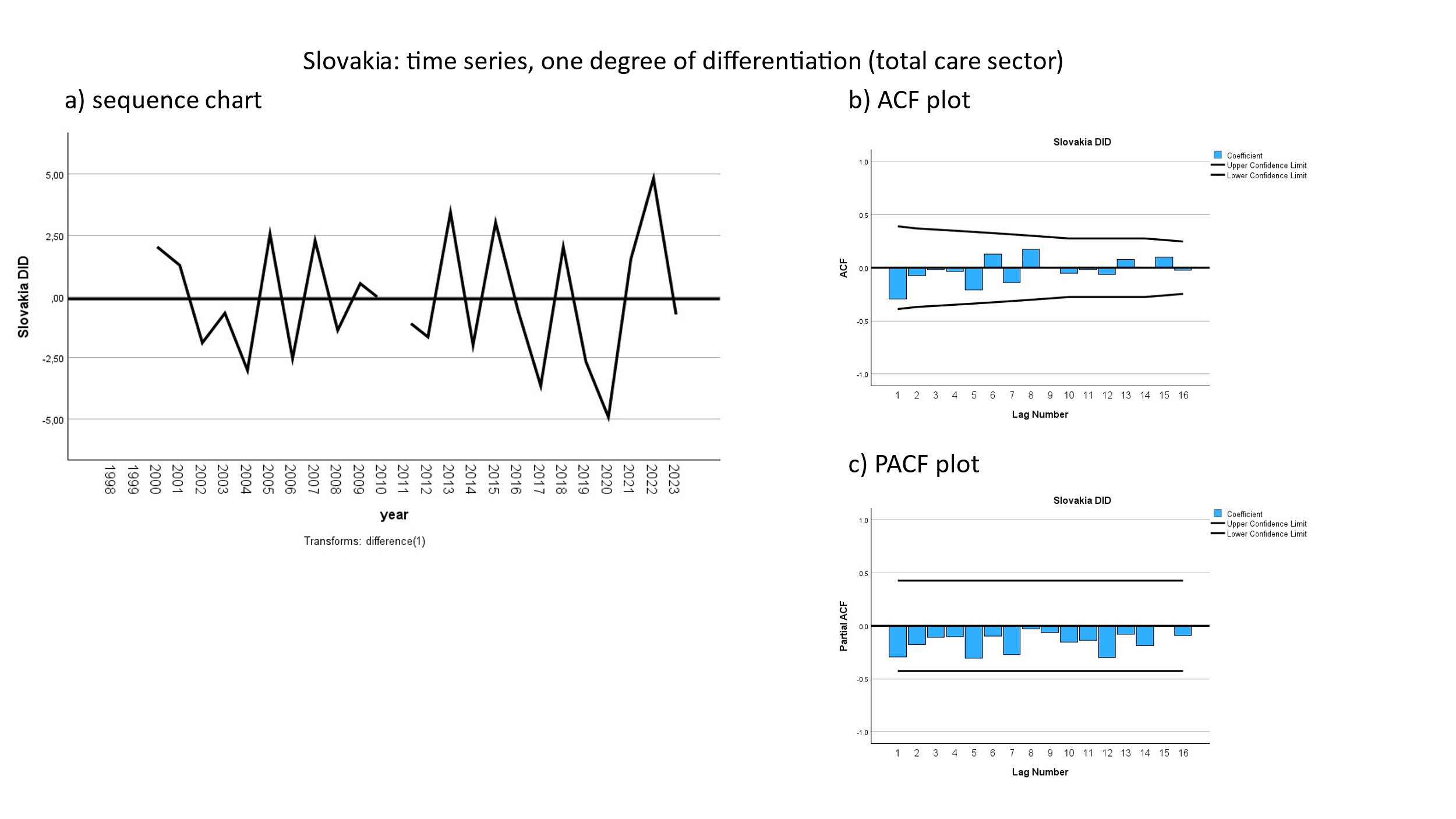


***Fig. S57:*** *Differentiated time series of ATC class J01 for Spain. In a) the sequence chart of consumption in DID is shown, while b) displays the ACF and c) the PACF plot of the autocorrelation. Stationarity can be seen in a roughly stable trend in both the sequence chart and the autocorrelation plots.*


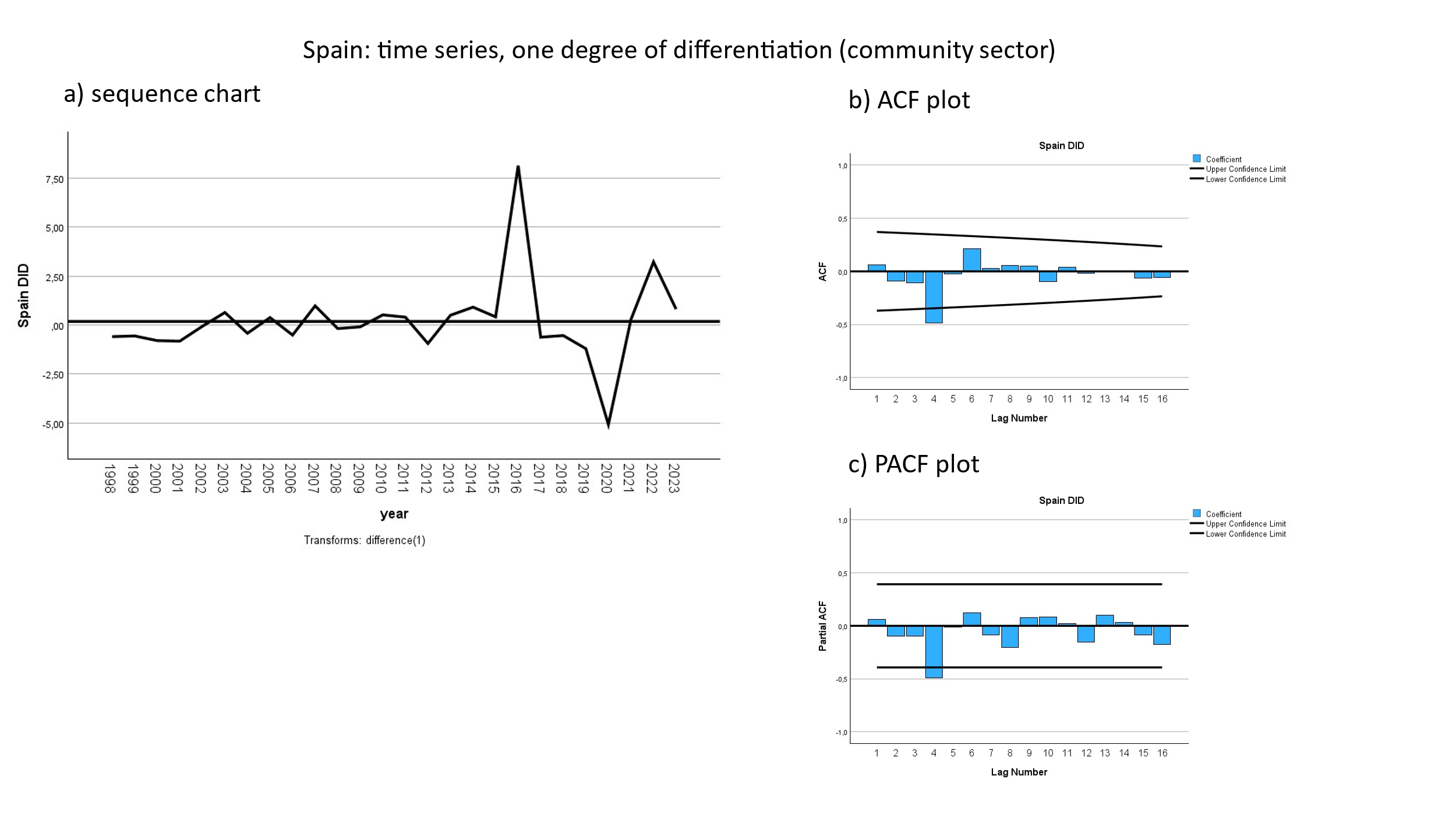


***Fig. S58:*** *Differentiated time series of ATC class J01 for Sweden. In a) the sequence chart of consumption in DID is shown, while b) displays the ACF and c) the PACF plot of the autocorrelation. Stationarity can be seen in a roughly stable trend in both the sequence chart and the autocorrelation plots.*


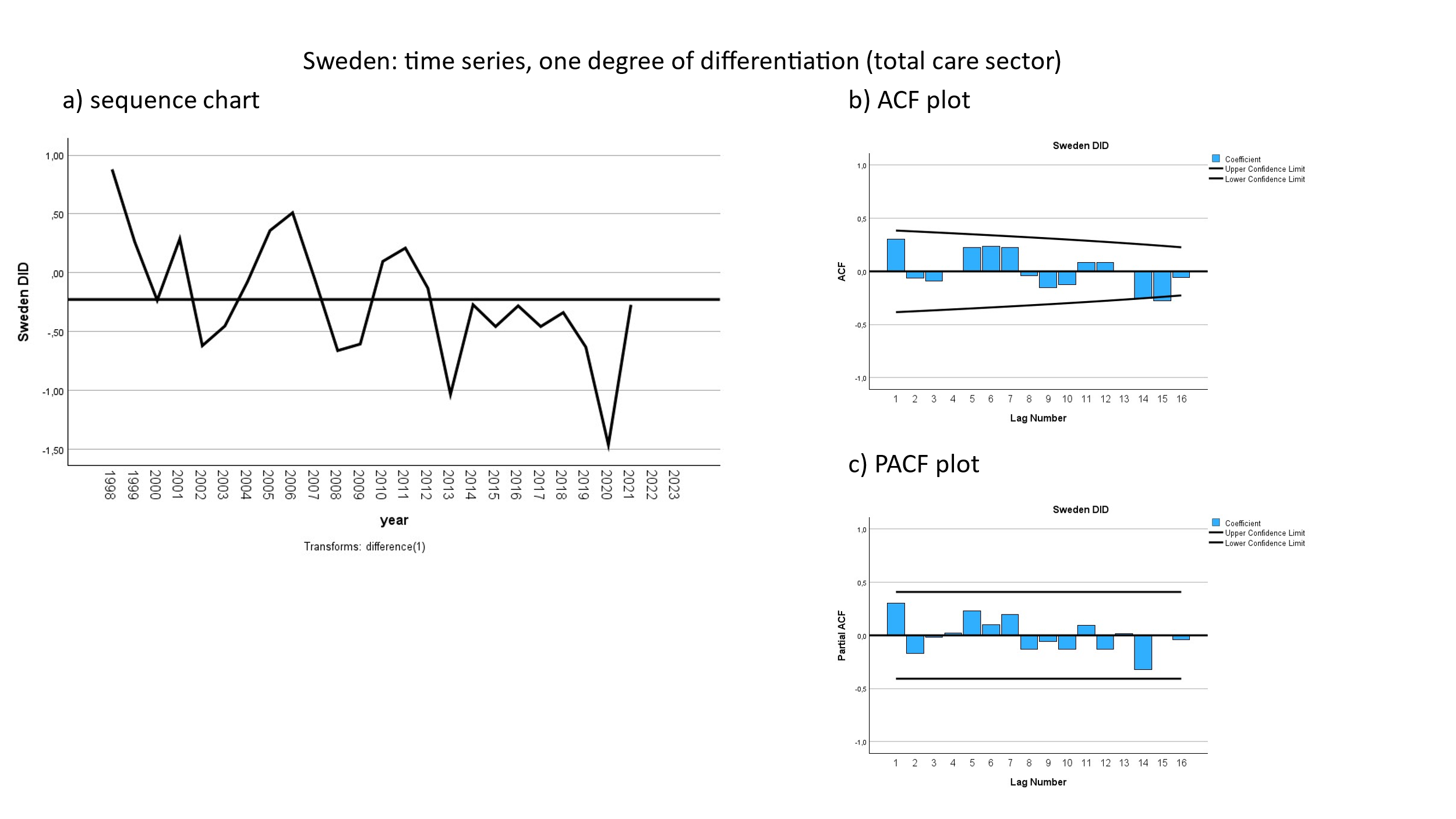


***Fig. S59:*** *Differentiated time series of ATC class J01 for the United Kingdom. In a) the sequence chart of consumption in DID is shown, while b) displays the ACF and c) the PACF plot of the autocorrelation. Stationarity can be seen in a roughly stable trend in both the sequence chart and the autocorrelation plots.*


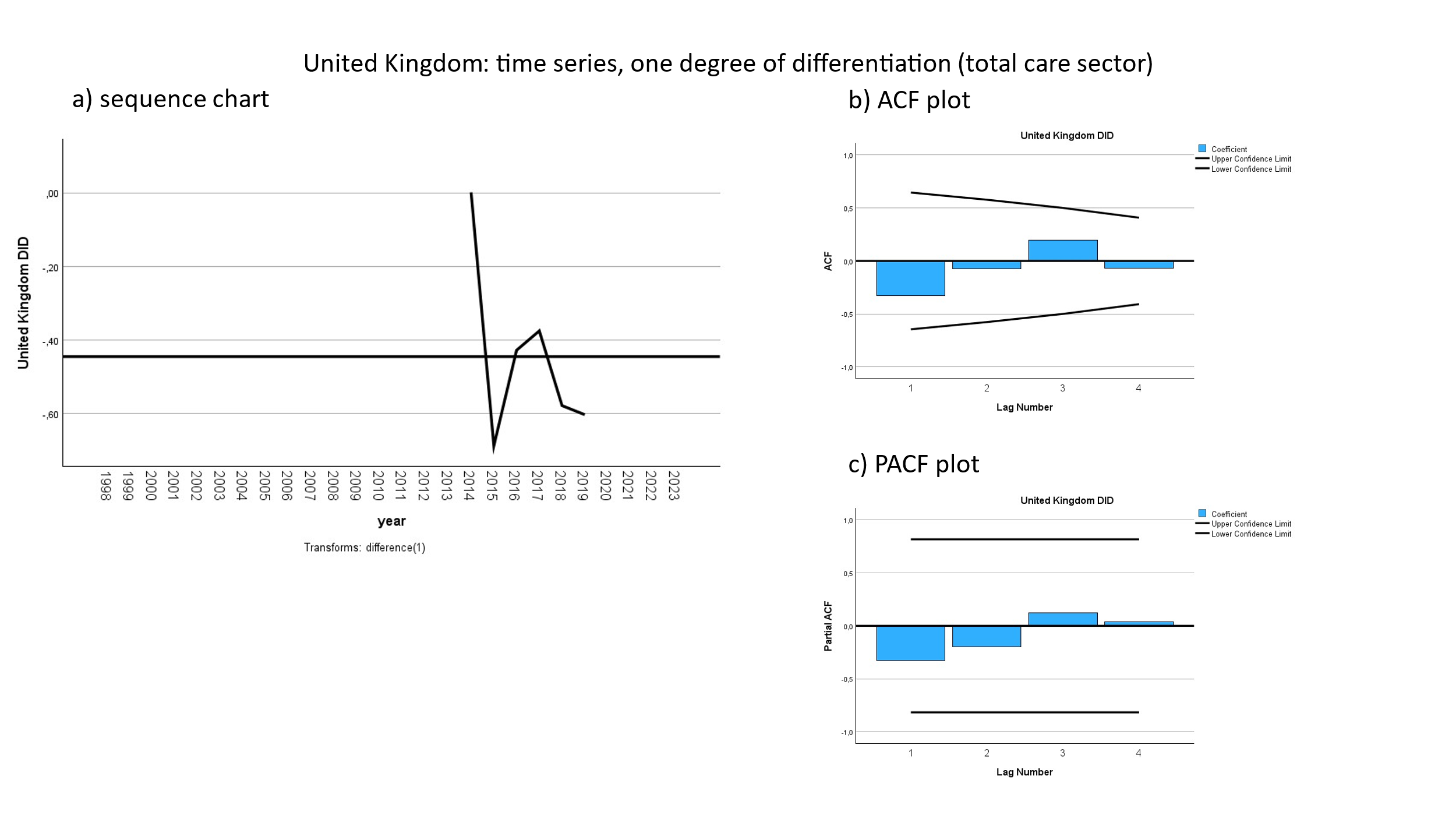


***Fig. S60:*** *Original time series of ATC class J01 for Australia. In a) the sequence chart of consumption in DID is shown, while b) displays the ACF plot of the autocorrelation. The non-stationarity can be seen in the visible trend in both the sequence chart and the ACF plot.*


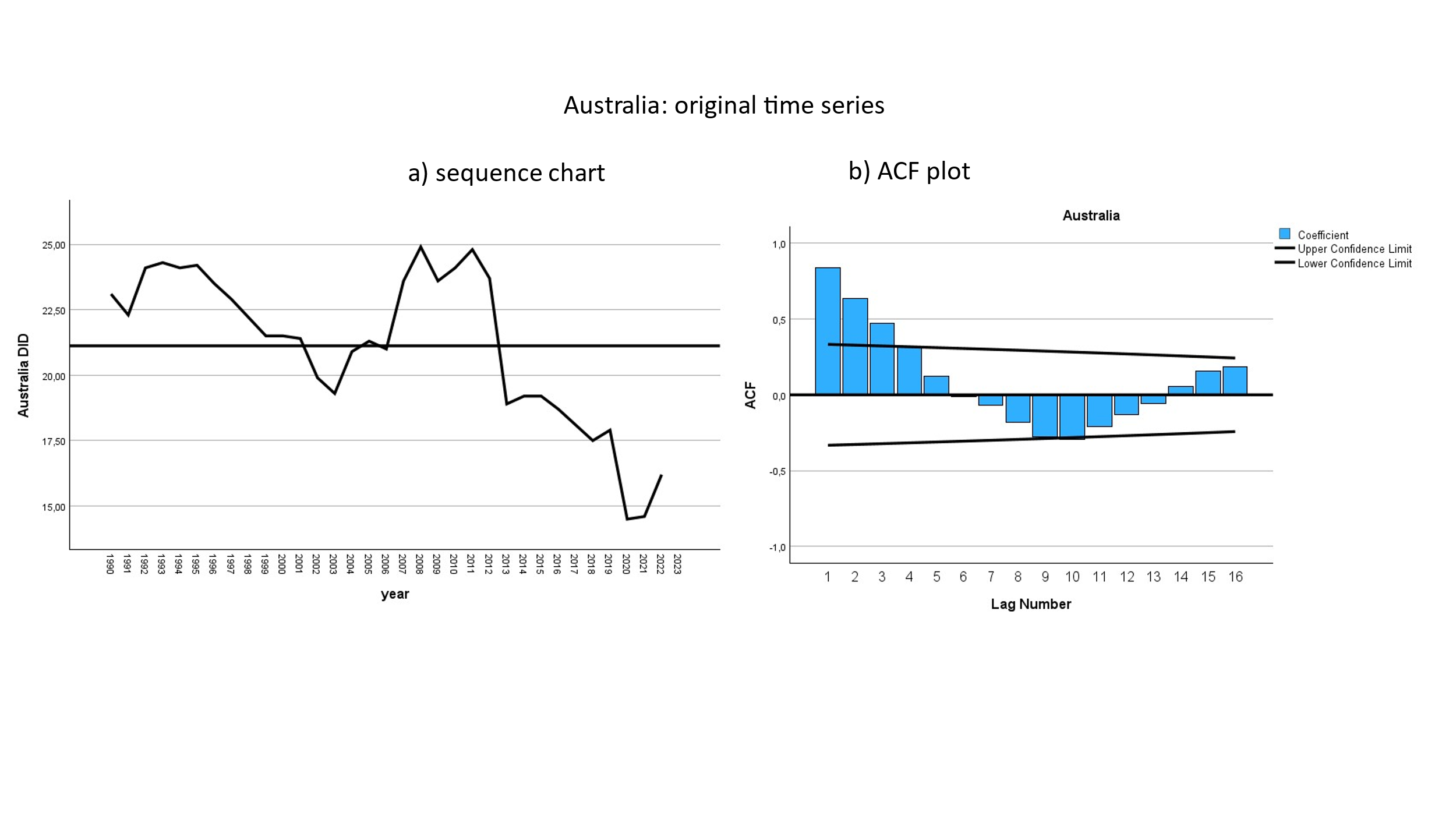


***Fig. S61:*** *Original time series of ATC class J01 for Canada. In a) the sequence chart of consumption in DID is shown, while b) displays the ACF plot of the autocorrelation. The non-stationarity can be seen in the visible trend in both the sequence chart and the ACF plot.*


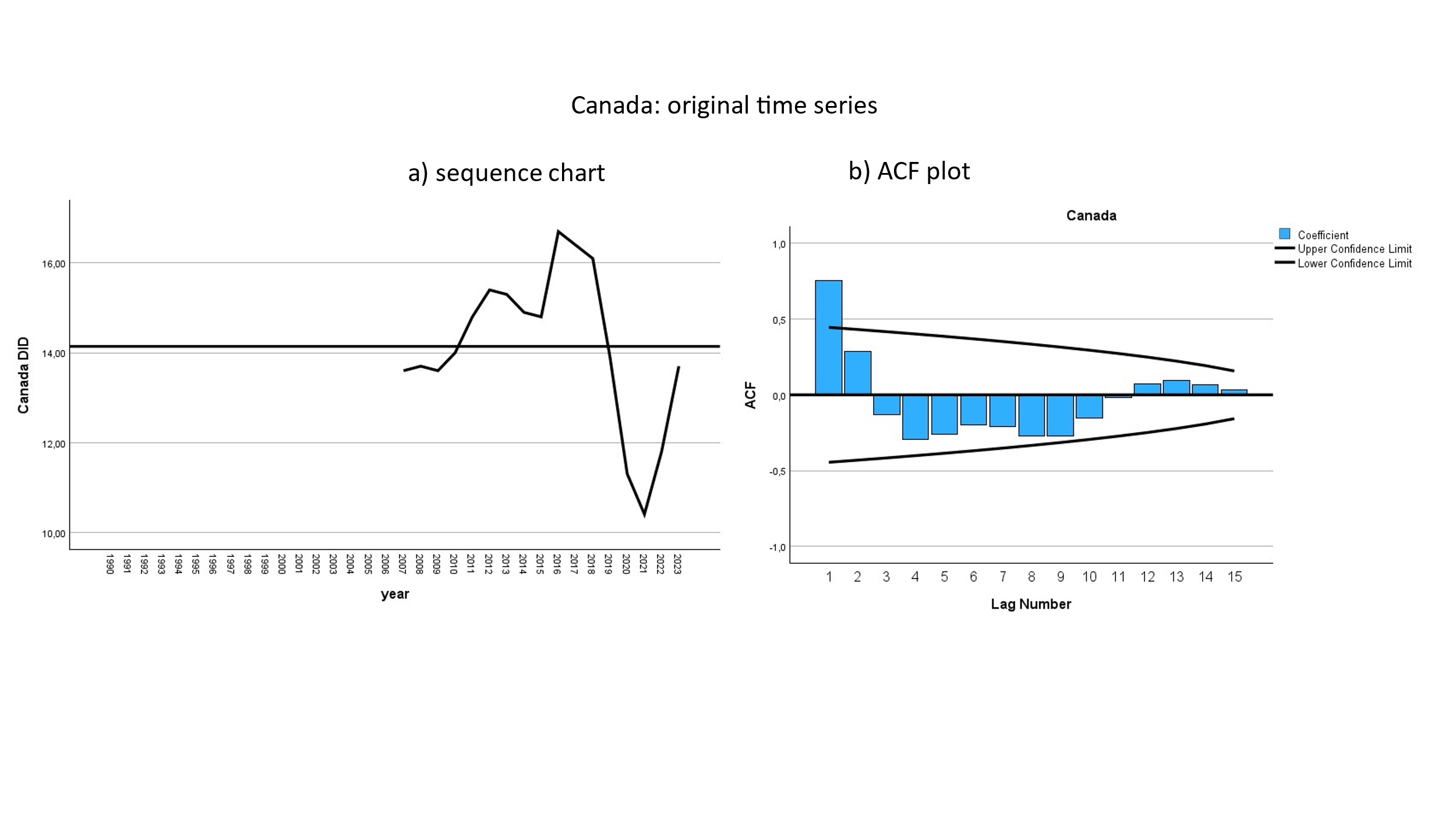


***Fig. S62:*** *Original time series of ATC class J01 for Chile. In a) the sequence chart of consumption in DID is shown, while b) displays the ACF plot of the autocorrelation. The non-stationarity can be seen in the visible trend in both the sequence chart and the ACF plot.*


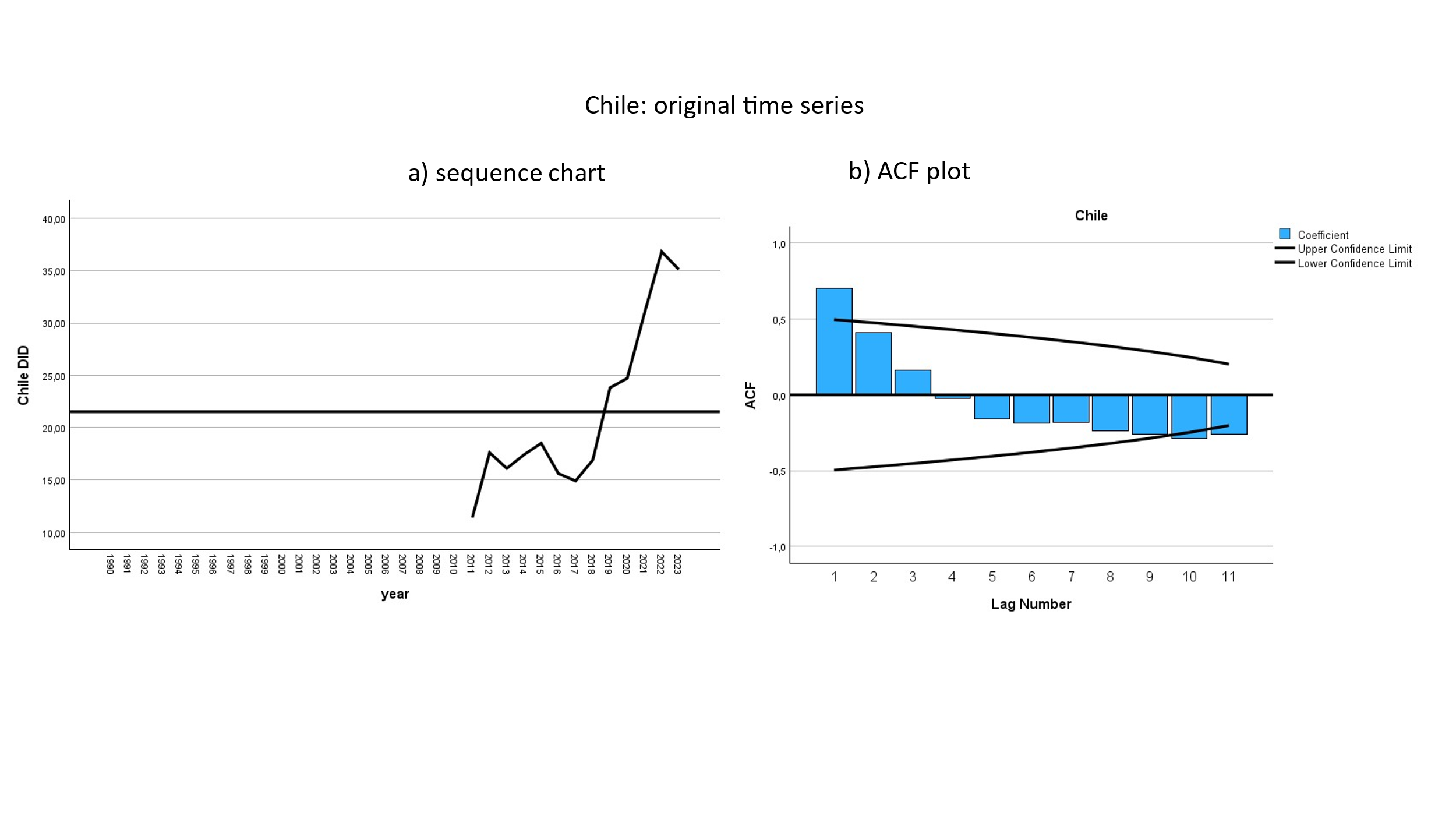


***Fig. S63:*** *Original time series of ATC class J01 for Costa Rica. In a) the sequence chart of consumption in DID is shown, while b) displays the ACF plot of the autocorrelation. The non-stationarity can be seen in the visible trend in both the sequence chart and the ACF plot.*


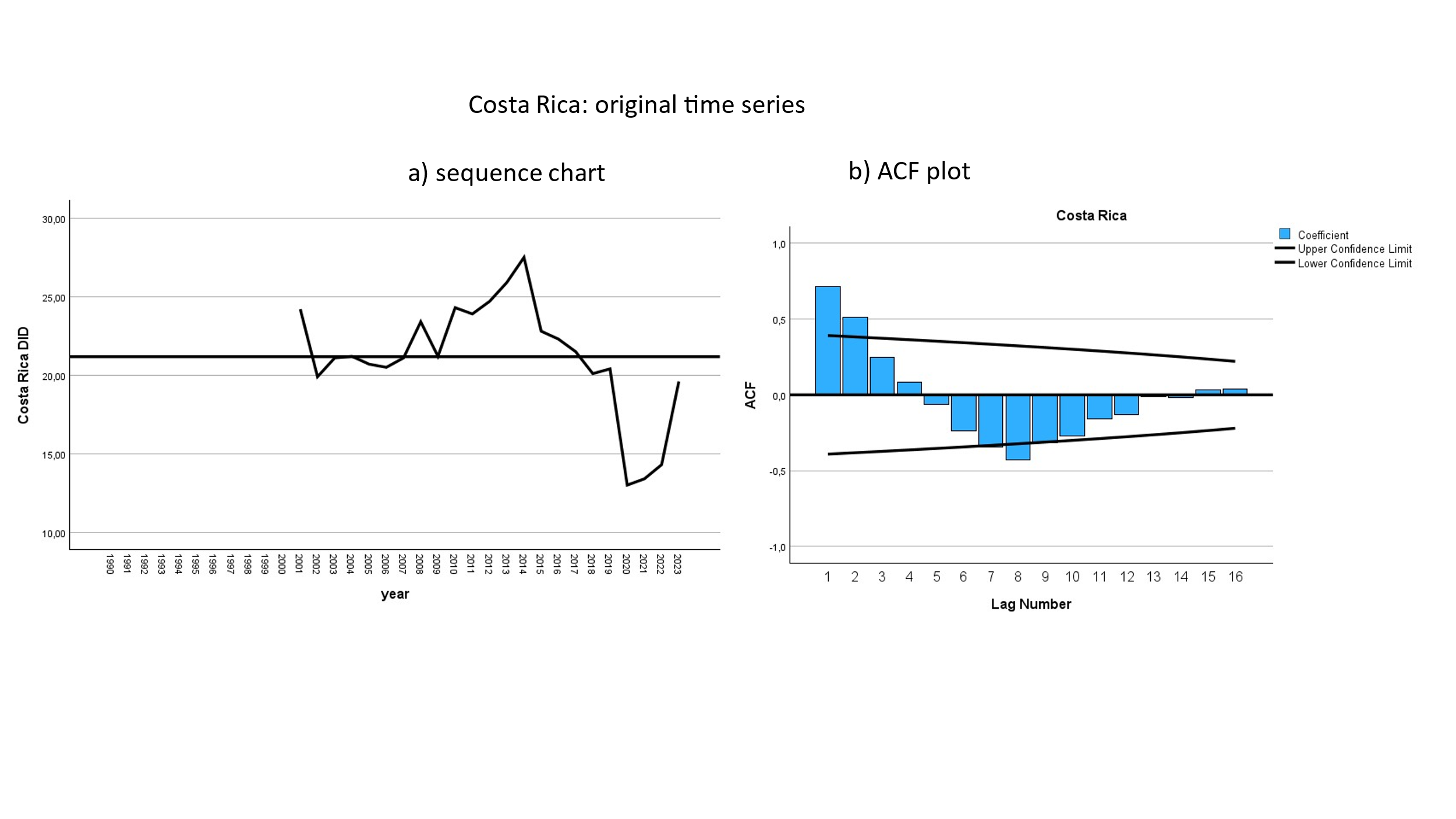


***Fig. S64:*** *Original time series of ATC class J01 for Israel. In a) the sequence chart of consumption in DID is shown, while b) displays the ACF plot of the autocorrelation. The non-stationarity can be seen in the visible trend in both the sequence chart and the ACF plot.*


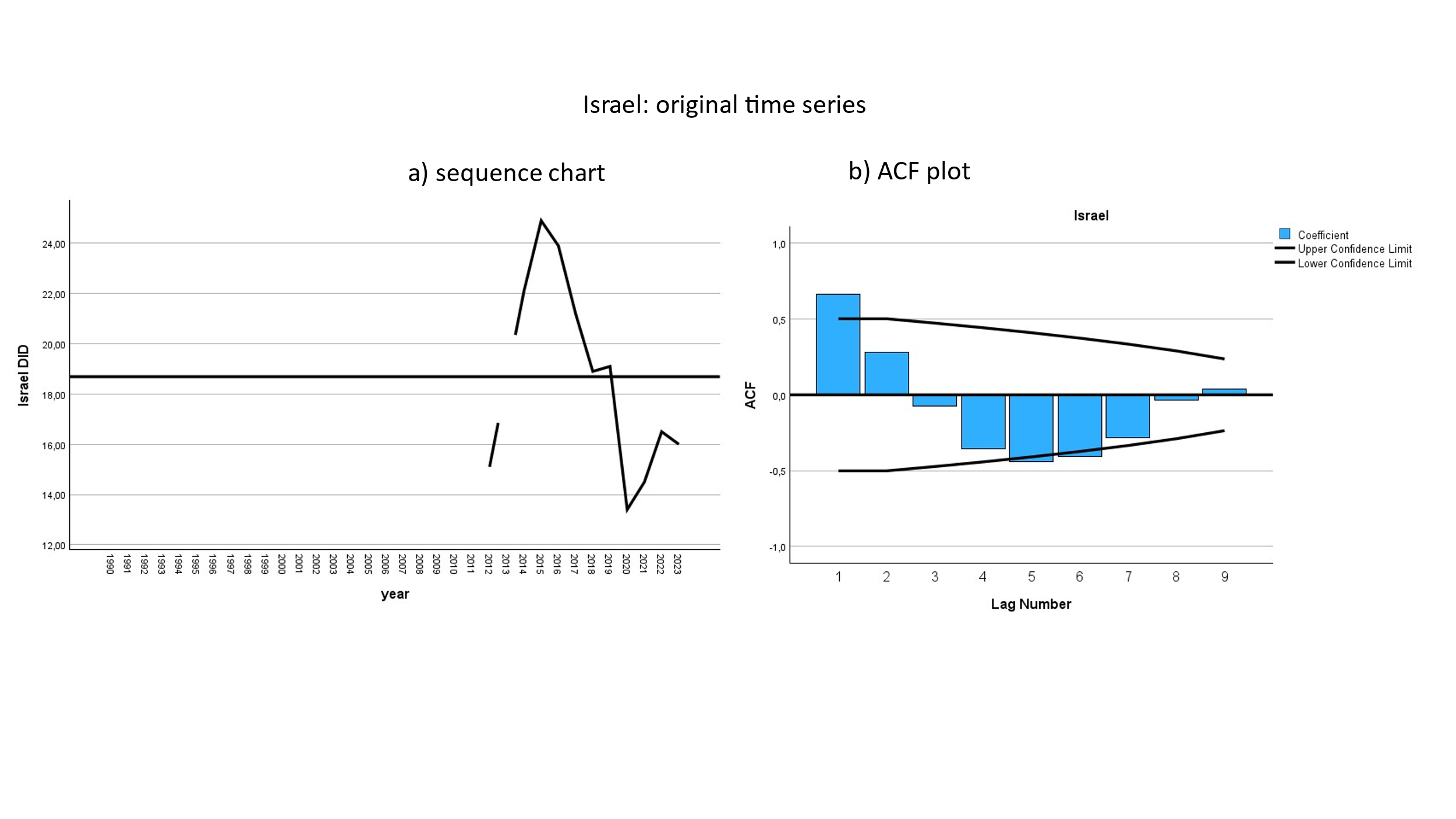


***Fig. S65:*** *Original time series of ATC class J01 for Japan. In a) the sequence chart of consumption in DID is shown, while b) displays the ACF plot of the autocorrelation. The non-stationarity can be seen in the visible trend in both the sequence chart and the ACF plot.*


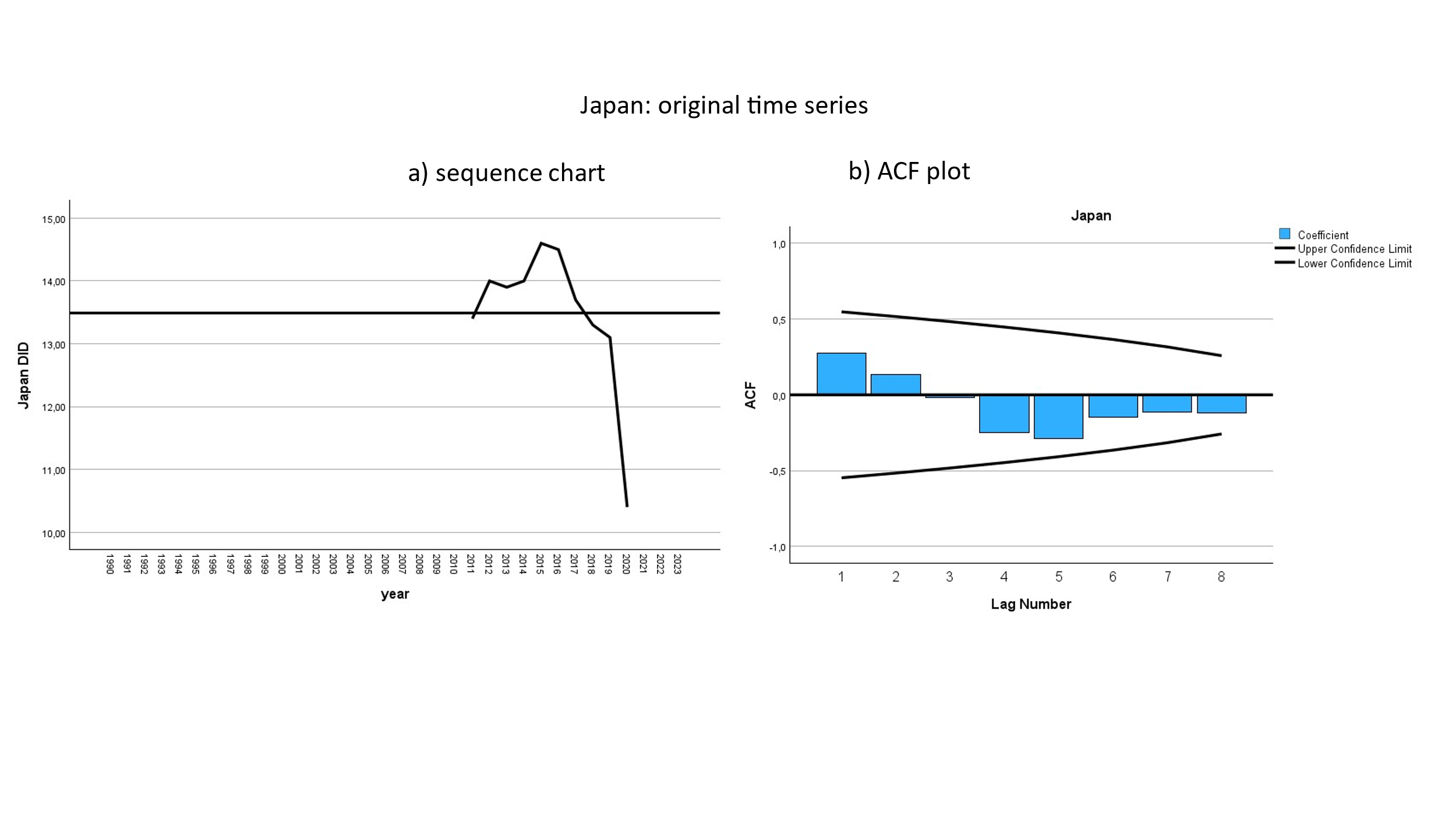


***Fig. S66:*** *Original time series of ATC class J01 for Korea. In a) the sequence chart of consumption in DID is shown, while b) displays the ACF plot of the autocorrelation. The non-stationarity can be seen in the visible trend in both the sequence chart and the ACF plot.*


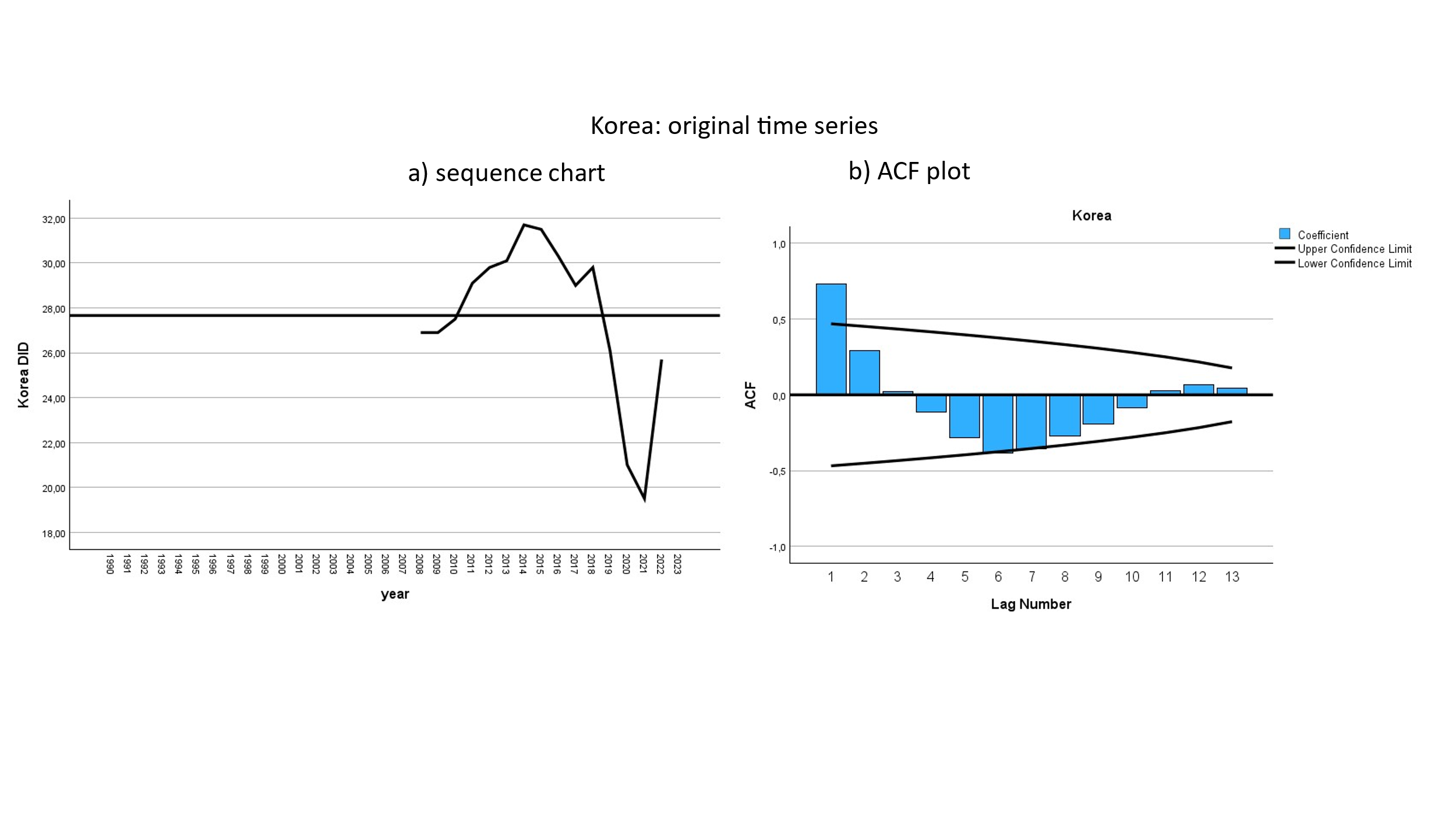


***Fig. S67:*** *Differentiated time series of ATC class J01 for Australia. In a) the sequence chart of consumption in DID is shown, while b) displays the ACF and c) the PACF plot of the autocorrelation. Stationarity can be seen in a roughly stable trend in both the sequence chart and the autocorrelation plots.*


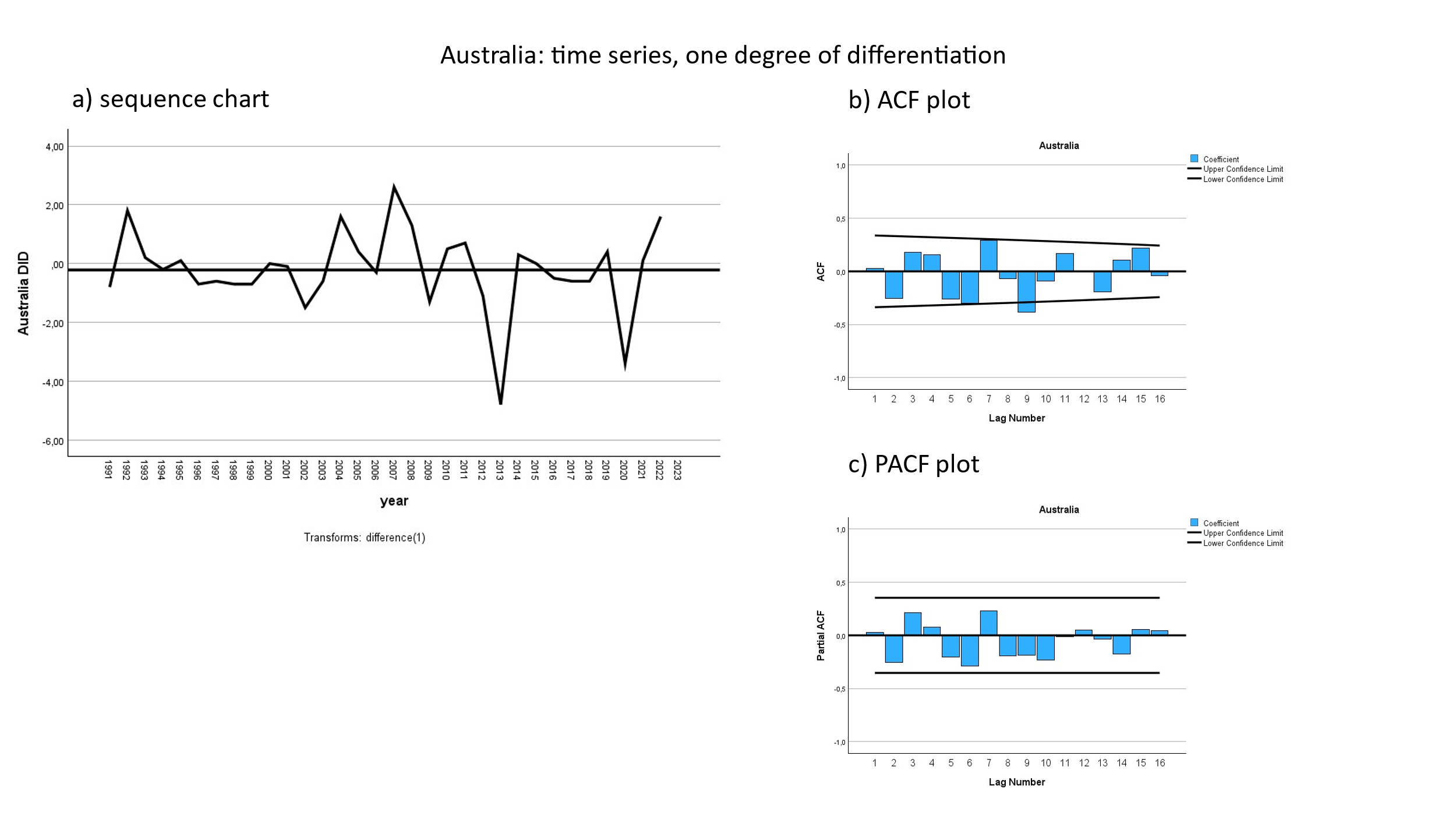


***Fig. S68:*** *Differentiated time series of ATC class J01 for Canada. In a) the sequence chart of consumption in DID is shown, while b) displays the ACF and c) the PACF plot of the autocorrelation. Stationarity can be seen in a roughly stable trend in both the sequence chart and the autocorrelation plots.*


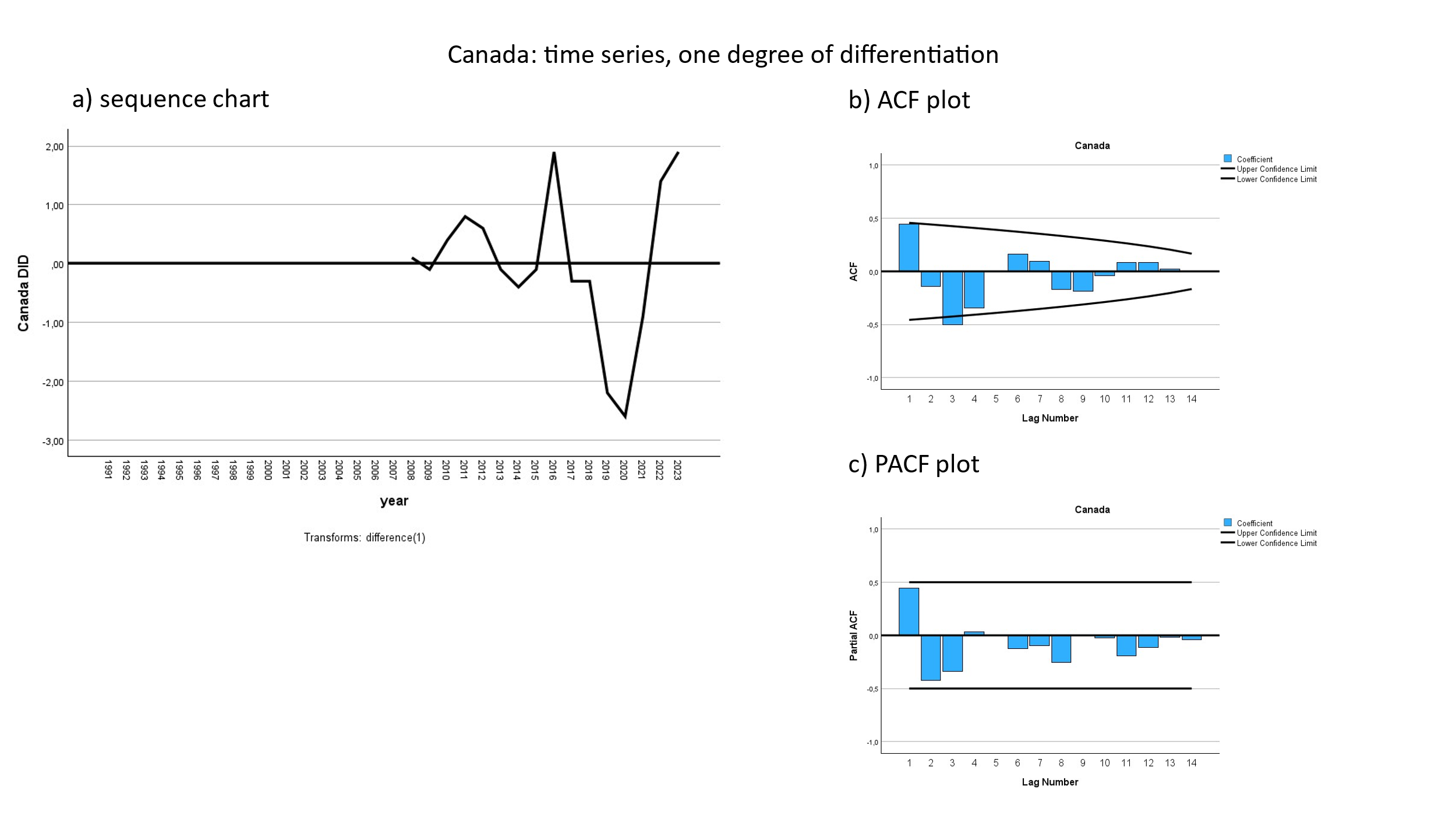


***Fig. S69:*** *Differentiated time series of ATC class J01 for Chile. In a) the sequence chart of consumption in DID is shown, while b) displays the ACF and c) the PACF plot of the autocorrelation. Stationarity can be seen in a roughly stable trend in both the sequence chart and the autocorrelation plots.*


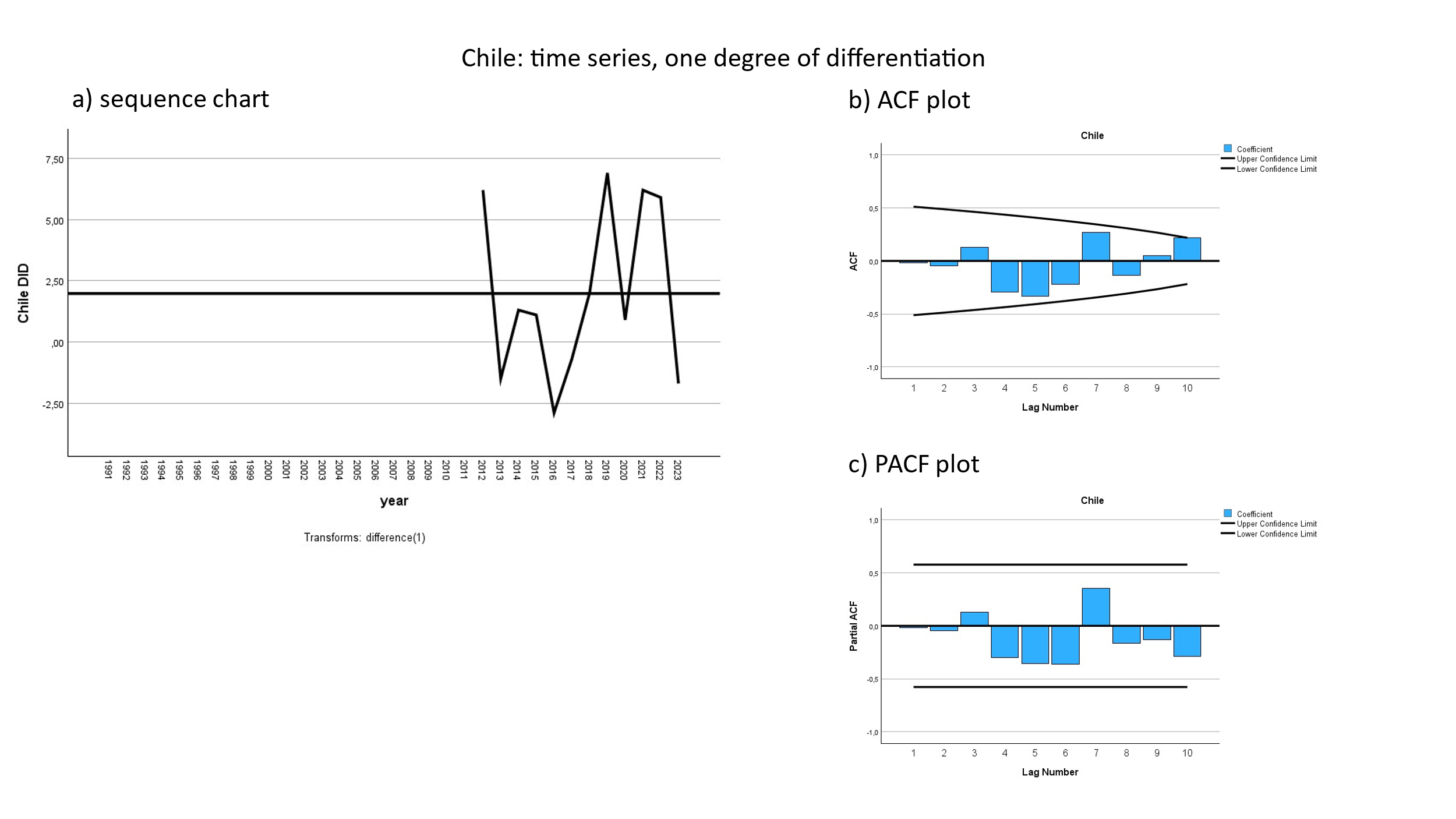


***Fig. S70:*** *Differentiated time series of ATC class J01 for Costa Rica. In a) the sequence chart of consumption in DID is shown, while b) displays the ACF and c) the PACF plot of the autocorrelation. Stationarity can be seen in a roughly stable trend in both the sequence chart and the autocorrelation plots.*


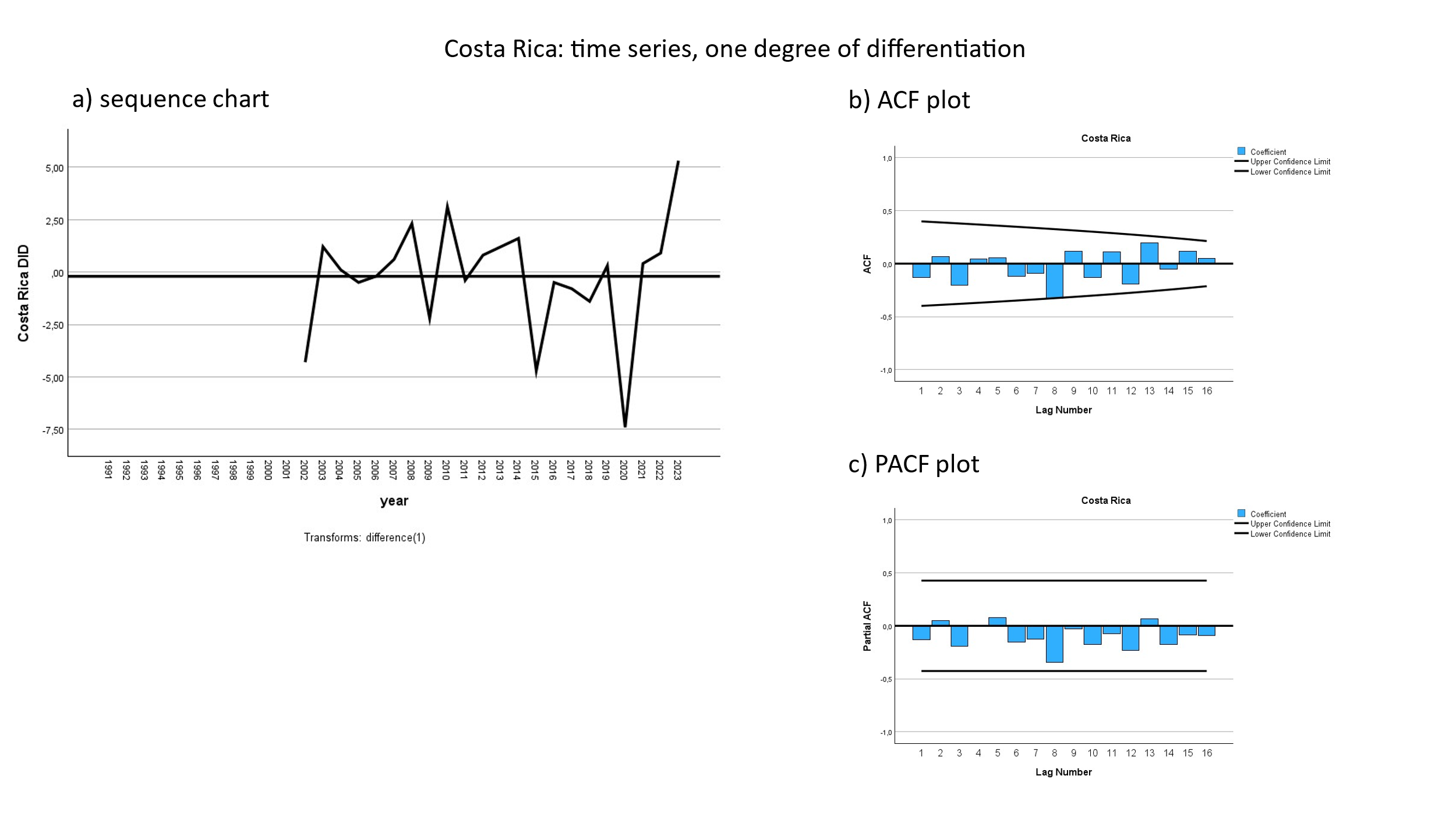


***Fig. S71:*** *Differentiated time series of ATC class J01 for Israel. In a) the sequence chart of consumption in DID is shown, while b) displays the ACF and c) the PACF plot of the autocorrelation. Stationarity can be seen in a roughly stable trend in both the sequence chart and the autocorrelation plots.*


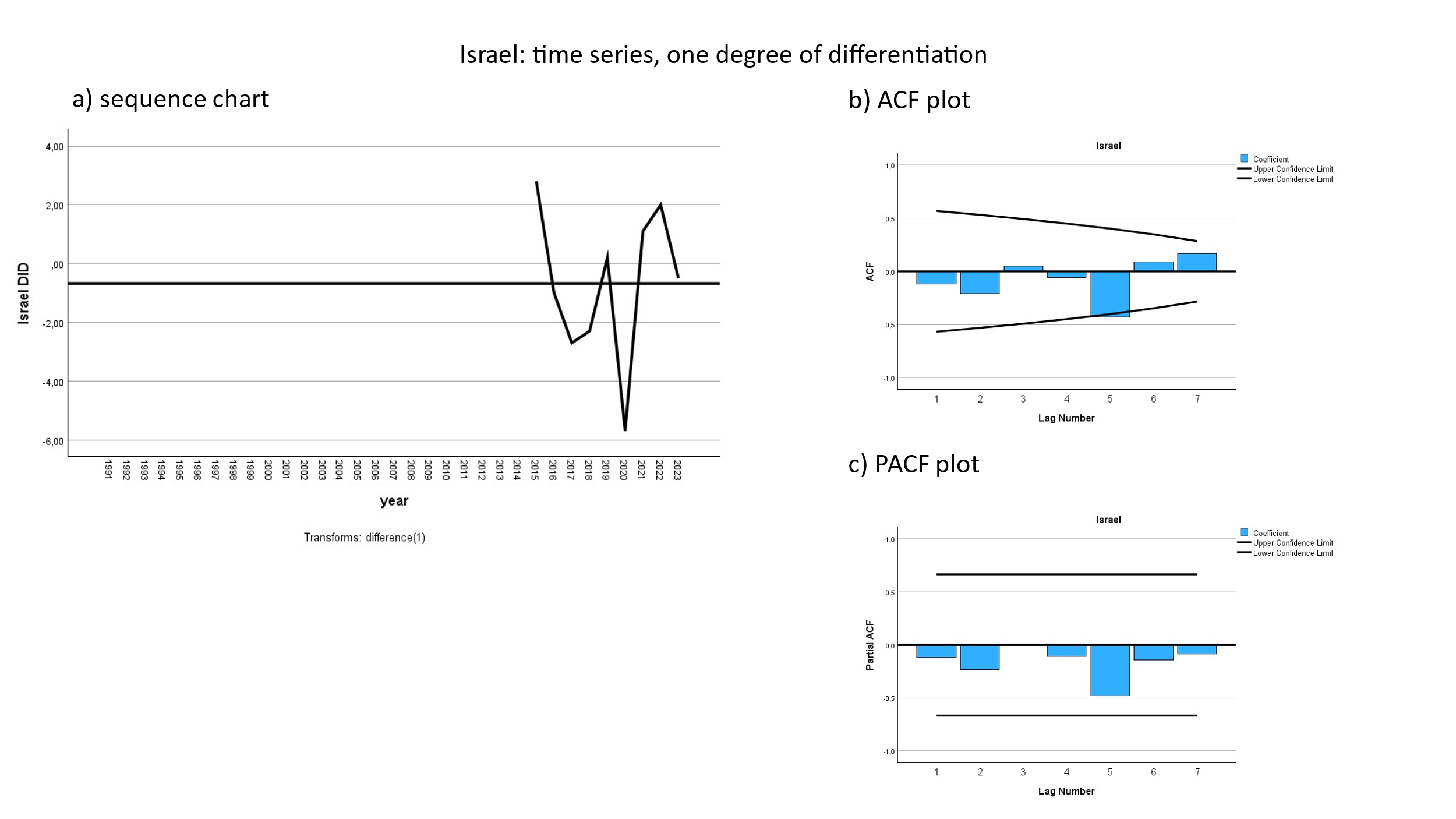


***Fig. S72:*** *Differentiated time series of ATC class J01 for Japan. In a) the sequence chart of consumption in DID is shown, while b) displays the ACF and c) the PACF plot of the autocorrelation. Stationarity can be seen in a roughly stable trend in both the sequence chart and the autocorrelation plots.*


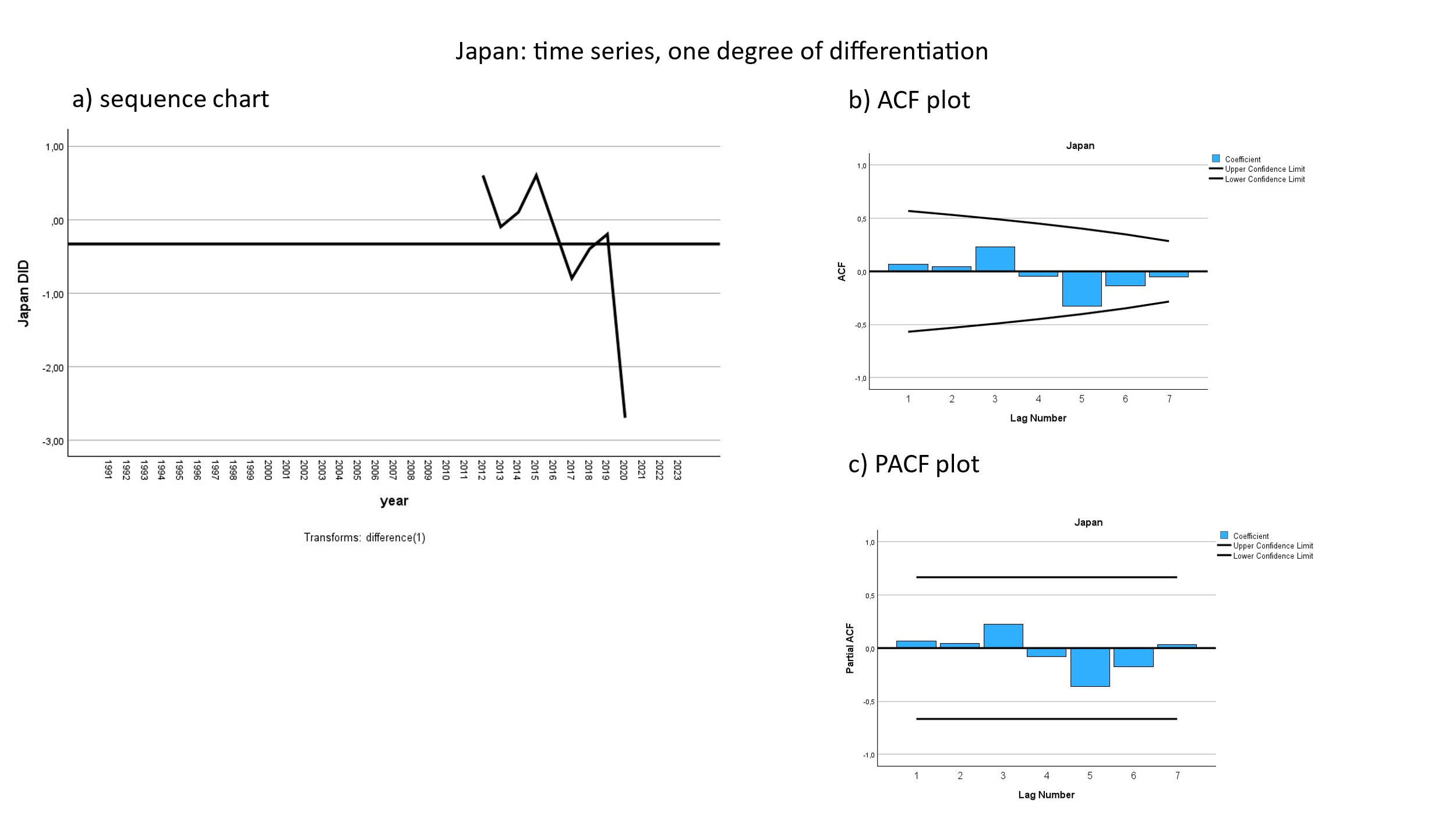


***Fig. S73:*** *Differentiated time series of ATC class J01 for Korea. In a) the sequence chart of consumption in DID is shown, while b) displays the ACF and c) the PACF plot of the autocorrelation. Stationarity can be seen in a roughly stable trend in both the sequence chart and the autocorrelation plots.*


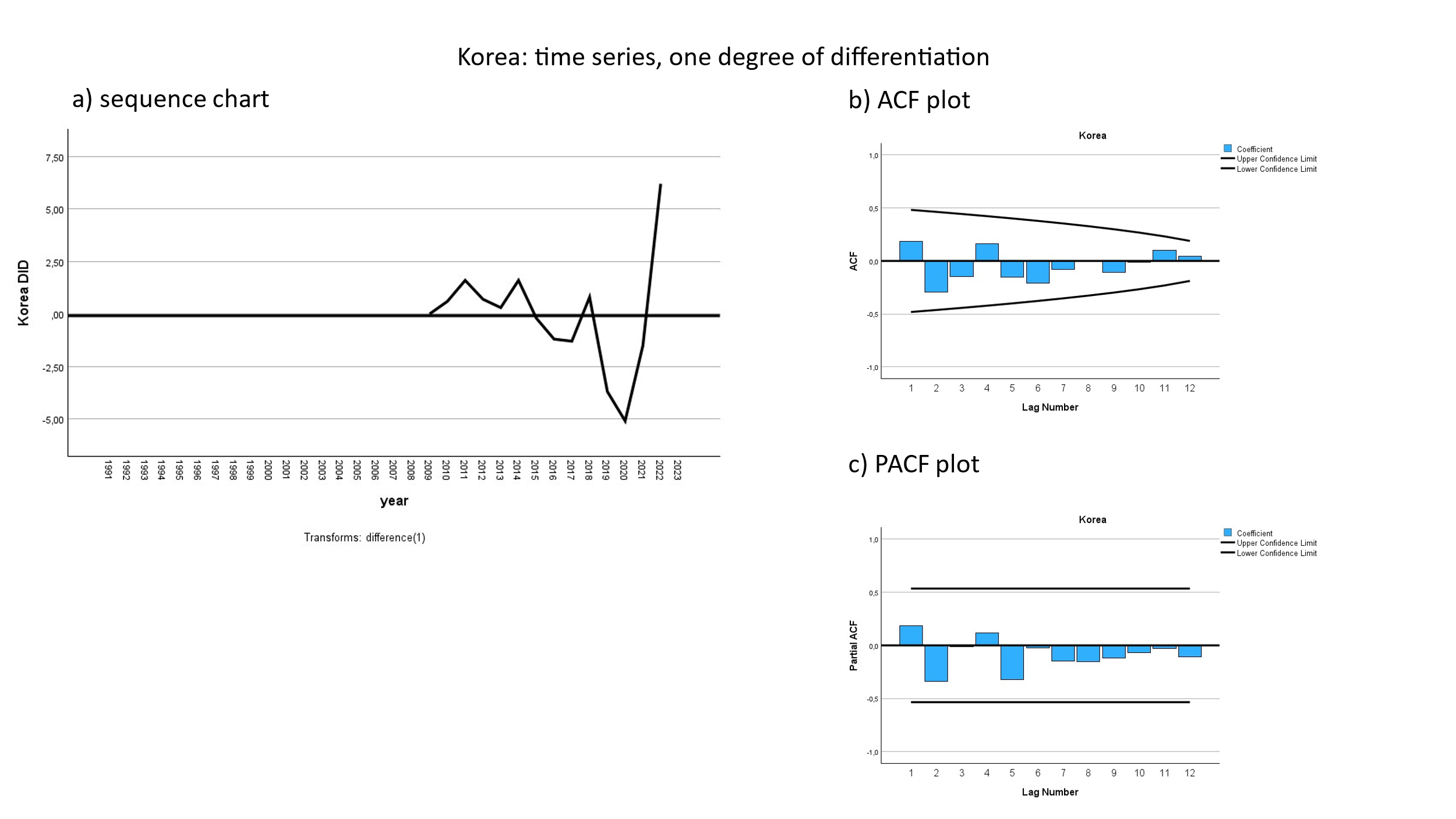

Supplement: Supplementary file 1 — Supplementary file1 (DOCX 30668 KB) [file 210_2025_3887_MOESM1_ESM.docx]
